# Comprehensive Evaluation of Usnic Acid as a Potential Drug Candidate for Triple-Negative Breast Cancer: Insights from Transcriptomic, Proteomic, and In Vivo Analyses

**DOI:** 10.3390/molecules30214281

**Published:** 2025-11-04

**Authors:** Ümmügülsüm Tanman, Mehmet Kürşat Derici, Mine Türktaş, Demet Cansaran-Duman

**Affiliations:** 1 Biotechnology Institute, Ankara University, Ankara 06135, Türkiye; 2 Department of Medical Pharmacology, Gulhane Faculty of Medicine, University of Health Sciences, Ankara 06290, Türkiye; 3 Department of Biology, Faculty of Science, Gazi University, Ankara 06560, Türkiye

**Keywords:** TNBC, usnic acid, RNA sequence, proteome, molecular mechanism

## Abstract

Background: Triple-negative breast cancer (TNBC) is an aggressive subtype of breast cancer with limited treatment options, prompting extensive research into novel therapeutics. This study presents a comprehensive molecular characterization of usnic acid in TNBC using transcriptomic, proteomic, and in vivo analyses. Results: Transcriptome profiling identified 974 differentially expressed genes (201 upregulated, 773 downregulated; *p* ≤ 0.05, FC ≥ 2) between control and usnic acid-treated MDA-MB-231 cells, while 4956 DEGs were detected between usnic acid-treated normal epithelial and TNBC cells. Proteomic analysis revealed significant changes in 372 proteins (50 upregulated and 322 downregulated). Functional enrichment analyses indicated that usnic acid modulates key oncogenic pathways, including gonadotropin, CCKR, integrin–ECM signaling, and lipid/energy metabolism. Flow cytometry confirmed increased apoptosis, evidenced by upregulation of pro-apoptotic genes and suppression of anti-apoptotic genes. In vivo xenograft models further validated the tumor-suppressive effects of usnic acid. Conclusions: In light of the findings, this study constitutes the first comprehensive integrated transcriptomic and proteomic evaluation of usnic acid in TNBC, supported by functional and in vivo validation. Collectively, the results position usnic acid as a compelling therapeutic candidate that has successfully passed key in vitro and in vivo preclinical evaluations, warranting further investigation in advanced preclinical models and potential translation toward clinical development for TNBC.

## 1. Introduction

Triple negative breast cancer (TNBC) is a subtype of breast cancer that does not express estrogen receptors (ERs), progesterone receptors (PRs), or HER2-neu. Therefore, successful results cannot be obtained using HER2-targeted or hormone therapy options. Although various treatment options are available, chemotherapeutic agents are commonly used to treat TNBC due to its heterogeneous molecular and clinical characteristics [1].

Chemotherapy drugs used in routine treatment include the DNA-intercalating agent anthracycline; the topoisomerase II inhibitor doxorubicin; alkylating agents, such as cyclophosphamide; the anti-microtubule agent taxane; and the anti-metabolite fluorouracil (5-FU) [2]. Patients with end-stage triple-negative breast cancer (TNBC) are treated with the anti-metabolites capecitabine and gemcitabine, the non-taxane microtubule inhibitor eribulin, and the DNA cross-linker platinum. Chemotherapeutic agents that target DNA and replication processes in aggressively proliferating cells are used to treat many cancer types and are not specific to TNBC. However, patient response to chemotherapeutics is short-lived, with rapid recurrence and metastasis to internal organs and the brain being common, particularly in aggressive cancer types such as TNBC. Furthermore, chemotherapy causes toxic effects and drug resistance over time [2,3]. Drug resistance is caused by the interaction of numerous factors and signaling pathways [4]. Emerging drug resistance remains a major challenge; therefore, identifying new therapeutic agents and their molecular mechanisms is vital. Recently, TNBC patients have been treated with oral drugs characterized by small molecules due to their selectivity, low toxicity, ease of administration, and ability to pass through the blood–brain barrier. Significant progress has been made in developing an increasing number of approved or investigational small-molecule drugs targeting various kinases, proteasomes, epigenetic regulatory proteins, and other targets, driven by clinical trials for the treatment of TNBC [5]. Examples of these drugs and their targets include erlotinib (Tarceva), an epidermal growth factor receptor (EGFR) inhibitor; apatinib, a vascular endothelial growth factor (VEGF) inhibitor; buparlisib (BKM120), a phosphatidylinositol-3-kinase (PI3K) inhibitor; ipatasertib (GDC-0068), an AKT inhibitor; trilaciclib (G1T28), a cyclin-dependent kinase (CDK) 4/6 inhibitor; sunitinib, a multi-kinase inhibitor; and pamiparib (BGB-290), a poly(ADP-ribose) polymerase (PARP) inhibitor [5]. Despite advances in drug research, there are still no effective treatment options for TNBC patients due to the need for prolonged treatment, side effects, and drug resistance. This has led researchers to investigate new molecules.

Pharmacotranscriptomic and proteomic analyses are essential at every stage of drug research. These analyses play a key role in determining the efficacy and safety of small molecules, elucidating their mechanisms of action, and assessing the potential of biomarkers [6]. They also allow drugs to be optimized, drug resistance to be overcome, and personalized therapeutic approaches to be developed. Consequently, such molecular analyses accelerate the drug development process and reveal more effective treatment strategies [7]. The molecular characterization of small molecules in TNBC will contribute to drug development by identifying new molecules or providing in-depth characterization of specific molecules, thereby offering a broader understanding of their therapeutic potential.

In recent years, it has been shown that lichen-specific secondary metabolites may be effective in cancer treatment, and studies investigating their effects have increased [8]. The chemical structure of usnic acid, the secondary metabolite of lichen dibenzofuran, is C_18_H_16_O_7_, and its IUPAC name is 2,6-diacetyl-7,9-dihydroxy-8,9b-dimethyl-1,3(H,9bH) dibenzofurandione [9]. Usnic acid, which is solid, yellow, and bitter-sweet at room temperature, could be found in nature in D+ or L- forms or as a racemic mixture since it contains a chiral center [9]. Usnic acid is a naturally occurring dibenzofuran derivative predominantly found in various lichen species, such as *Usnea*, *Cladonia*, and *Lecanora*. This compound has attracted considerable scientific interest due to its broad spectrum of pharmacological activities, including antimicrobial, anti-inflammatory, antioxidant, and anti-cancer properties. Previous studies have demonstrated its cytotoxic effects in several cancer cell lines, including those of the lung, colon, and melanoma, primarily through the induction of apoptosis and the disruption of mitochondrial function. The anti-cancer activity of usnic acid has been demonstrated to involve programmed cell death, DNA fragmentation, cytoplasmic condensation, nuclear pyknosis, chromatin condensation, cell digestion by macrophages, and morphological changes resulting from cytoskeleton collapse driven by membrane-dependent apoptotic changes [10,11,12]. Mayer et al. demonstrated that the usnic acid molecule suppresses the proliferation of MCF-7 and MDA-MB-231 cells [13]. Bakorova et al. investigated the cytotoxic mechanisms of parietin, atranorin, usnic acid, and gyrophoric acid on A-2780 and HT-29 cancer cell lines. Usnic acid was found to be a more potent anti-cancer compound than atranorin, parietin, and gyrophoric acid. It caused a significant loss of mitochondrial membrane potential, accompanied by caspase-3 activation in HT-29 cells and phosphatidylserine externalization in both cell lines tested [14]. Dinçsoy and Cansaran-Duman investigated the inhibitory effect of usnic acid on cancer cell growth by measuring apoptotic gene expression levels using MTT analysis, determining that it inhibits *p53* and *Bcl-2* expression [15]. In addition, a microarray study by Cansaran-Duman et al. was performed to detect miRNAs that act on usnic acid in breast cancer cells (MDA-MB-231, MCF-7, and BT-474). They concluded that usnic acid-specific microRNAs are enriched in the TGF-beta, MAPK, and apoptosis pathways [8]. However, the holistic effect of usnic acid on TNBC breast cancer remains unclear. Given the urgent need for novel therapeutics in TNBC, for which there are no targeted treatment options, usnic acid is a promising candidate for further investigation. Its ability to interfere with multiple cellular pathways relevant to cancer progression positions it as a potential multi-target agent in the context of aggressive and treatment-resistant cancers, such as TNBC.

Although the anti-cancer potential of usnic acid has been demonstrated in various cancer cell lines through apoptosis and cell cycle regulation, its comprehensive molecular effects in triple-negative breast cancer (TNBC) remain largely unknown. Previous studies have primarily focused on individual pathways or selected gene/protein targets. In contrast, the present study is the first to comprehensively characterize usnic acid in TNBC, combining transcriptomic and proteomic analyses with in vitro and in vivo functional validation. This multi-omics approach has facilitated the identification of coordinated molecular networks that extend beyond apoptosis and ECM/integrin signaling. These networks may underlie the compound’s selective cytotoxicity in TNBC cells. Consequently, our research aims to corroborate previously documented anti-cancer properties and to elucidate system-level mechanisms and potential therapeutic targets of usnic acid within the specific context of TNBC biology.

Determining usnic acid-specific TNBC treatment targets has provided a new alternative treatment option for TNBC, a cancer for which treatment options are still insufficient. This study also comprehensively examines a new candidate molecule that can be used in combination drug therapy to overcome drug resistance in routine therapy.

## 2. Results

### 2.1. XCELLigence^®^ Analysis

**Figure 1:**
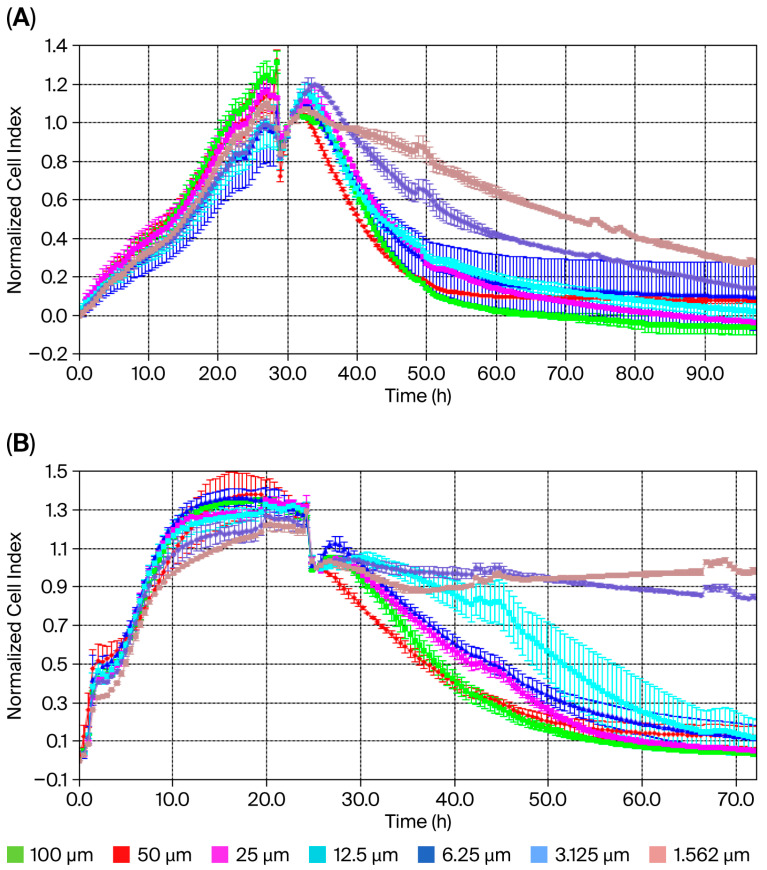
Cell index of usnic acid seeded at different concentrations, as monitored over 96 h on MDA-MB-231 (**A**) and MCF-12A (**B**) cells. Data represent the mean ± SEM (n = 3). Average change in cell index of usnic acid-exposed MDA-MB-231 cells compared to untreated cells over 96 h.

The XCELLigence^®^ RTCA S16 real-time cell analysis system enables the label-free, real-time, continuous monitoring of cell proliferation using impedance and microelectrodes. This offers a wide range of applications in cytotoxicity studies. The IC_50_ concentration for usnic acid was calculated based on the cell index (CI) at each measurement point of the study. Dose–response analysis using a four-parameter logistic model in the XCELLigence^®^ RTCA S16 software revealed IC_50_ concentrations of 22.0 µM (95% CI: 18.3–25.7 µM) for MDA-MB-231 cells and 60.0 µM (95% CI: 54.1–67.8 µM) for MCF-12A normal epithelial cells after 48 h (Figure 1A,B). These results demonstrate that TNBC cells are approximately 3-fold more sensitive to usnic acid than normal breast epithelial cells. Graphs showing cell viability as a function of CI vs. time are provided in Figure 1A,B. Cytotoxicity analysis showed that the usnic acid molecule inhibited 78% (±0.01) of MDA-MB-231 cells after 48 h (n = 3, *p* < 0.05). However, while usnic acid reduced the number of MDA-MB-231 cells, it inhibited the growth of breast epithelial cells by only 22%. In this case, usnic acid reduces breast cancer cell numbers without cytotoxic effects on normal epithelial cells. While the anti-proliferative potential of usnic acid on various cancer cells, including breast cancer cells, has been studied using dye-based methods such as MTT and WST, this is the first detailed study using XCELLigence^®^ analysis. In light of these results, usnic acid demonstrates favorable selectivity and meets the key pharmacological criteria for a TNBC drug candidate.

### 2.2. Transcriptome Analysis

Comprehensive transcriptomic analyses of MCF-12A and MDA-MB-231 cells in response to usnic acid treatment were carried out using RNA sequencing. Sequencing of the four libraries was performed using the Illumina platform, generating an average of 37 million high-quality raw reads per library. This corresponded to a sequencing depth of approximately 94.5 coverage of the expressed transcriptome. The reads were mapped to the human reference genome. The proportion of mapped reads ranged from 88% to 99% (see Table S1). A reference genome-guided transcript assembly of the aligned reads was then performed.

A total of 6997 genes were identified as differentially expressed (DEGs) in at least one library. These were then compared based on both cell type and usnic acid application (see Table 1). The general profile revealed that, although usnic acid application caused differences in both cell types compared to the control group, a significant difference was observed between MCF-12A and MDA-MB-231 cells (4007 DEGs). Notably, the highest number of DEGs was observed between usnic acid-treated MCF-12A and MDA-MB-231 breast cancer cells (4956 DEGs), indicating distinct responses to usnic acid treatment between the cell types. Another observation is that cancer cells have a slightly greater tendency for increased gene expression than MCF-12A cells. Most genes were upregulated (2283 and 2741), while fewer were downregulated (1724 and 2215) in cancer cells compared to breast epithelial cells.

One of the most striking results concerns the effects of usnic acid on both cell types. It appears that usnic acid treatment downregulated genes in both cell types rather than upregulating them. Compared to the MCF-12A library, 1050 genes were downregulated and only 513 were upregulated in the UA_MCF-12A library. Furthermore, this effect was much more pronounced in the MDA-MB-231 cells.

**Figure 2:**
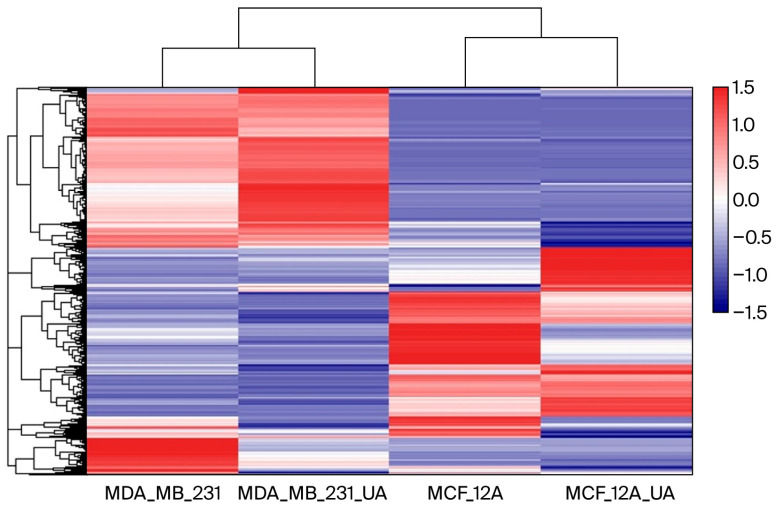
Heatmap of gene expression across samples. Color intensity represents relative expression levels, with blue indicating lower expression and red indicating higher expression.

A heatmap was generated using Log_2_-transformed FPKM values to normalize the dynamic range and visualize gene expression patterns across samples (Figure 2). As shown in the figure, the heatmap of differentially expressed genes (DEGs) clearly shows different clustering of gene expression between MDA-MB-231 cancer cells and breast epithelial MCF-12A cells. Although usnic acid treatment differentially affected gene expression in breast cancer and breast epithelial cells, it stimulated a group of genes in a similar way in both cell types, indicating a common effect of usnic acid across cell types. Notably, when the four libraries were compared, a small group of genes was found to be upregulated by usnic acid treatment in both cell types, indicating its effect regardless of cell type.

### 2.3. Functional Enrichment Analysis of the DEGs Between the Libraries

To understand the genes identified in the data, detailed annotations of their functions were performed using Gene Ontology (GO) and pathway analysis. To analyze the effect of usnic acid on MCF-12A cells, ontology and pathway analyses of 1563 differentially expressed genes (DEGs) were performed (Figure S1A). The results showed that usnic acid treatment mostly influenced genes related to transcription regulation, the apoptotic process, and cell division in MCF-12A cells. The most enriched pathways play vital roles in cancer biology, such as the Wnt signaling and gonadotropin-releasing hormone receptor pathways. CC ontology revealed significant enrichment in the nucleus, the cytosol, and the cytoplasm. When the DEGs were analyzed in detail (Figure S1B), it was observed that transcription regulator gene expression increased with usnic acid application, whereas cell division genes were the most downregulated upon treatment.

In contrast to breast epithelial cells, breast cancer cells responded differently to usnic acid application. A total of 974 DEGs were identified in MDA-MB-231 cells and their control libraries, and these were largely involved in cell division, cell adhesion processes, and transcription regulation (Figure S2A). Supporting this finding, Panther pathway analysis identified the integrin pathway as the most significant, which functions in cell–cell and cell–extracellular matrix adhesion. The enriched GO-CC annotation also suggested that the top enriched categories were membrane, extracellular exosome, extracellular space, and extracellular region. Interestingly, the upregulated genes in the usnic acid-treated MDA-MB-231 library were mainly related to the nucleus and nucleolus. Conversely, the most abundant CC terms for the downregulated genes of the MDA-MB-231 library were membrane, extracellular exosome, and extracellular space (Figure S2B). The results also showed that the integrin pathway was the most downregulated in the MDA-MB-231 library. CCKR signaling, which plays a role in the development and progression of various types of cancer, was found to be the most abundant upregulated pathway in the MDA-MB-231 library.

To understand the differences in the biological functions of DEGs between healthy and cancer cells, 4007 DEGs were analyzed (Figure S3A). As expected, the apoptotic process was found to be the most abundant GO-BP term among DEGs between breast cancer and breast epithelial cells in the absence of usnic acid treatment. Compared to MCF-12A breast epithelial cells, genes involved in the gonadotropin-releasing hormone receptor pathway were downregulated in MDA-MB-231 cells, while genes related to inflammation, chemokine and cytokine signaling, and the Wnt signaling pathway were upregulated (Figure S3B).

**Figure 3:**
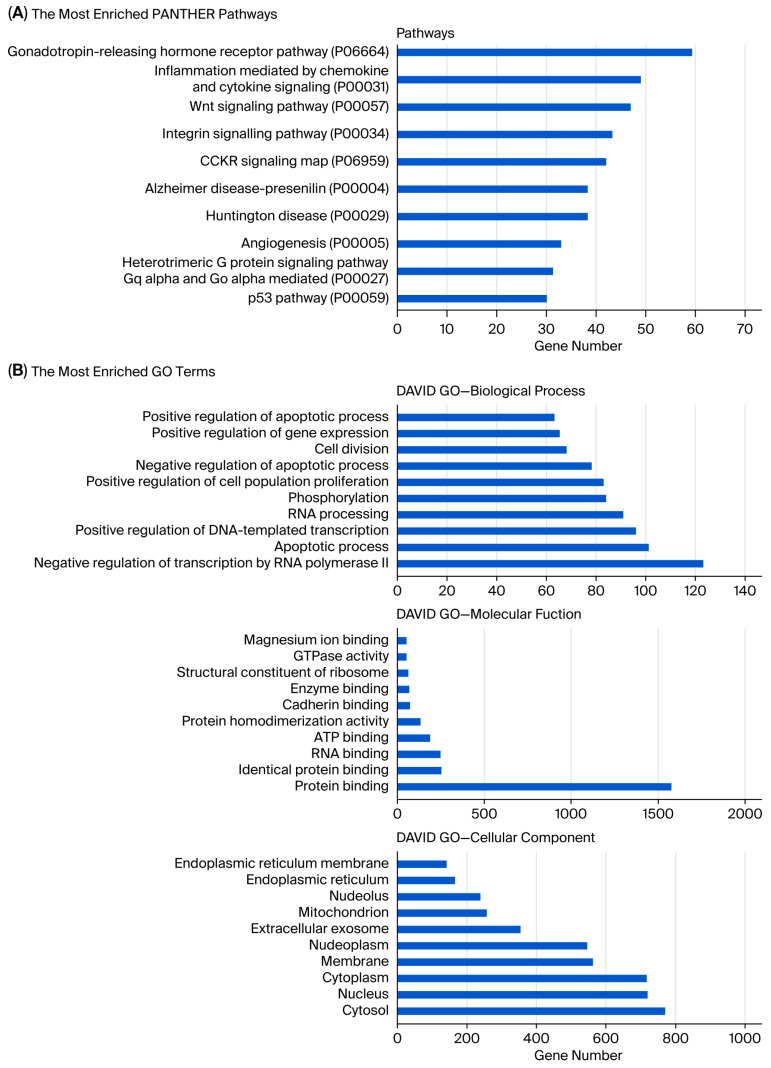

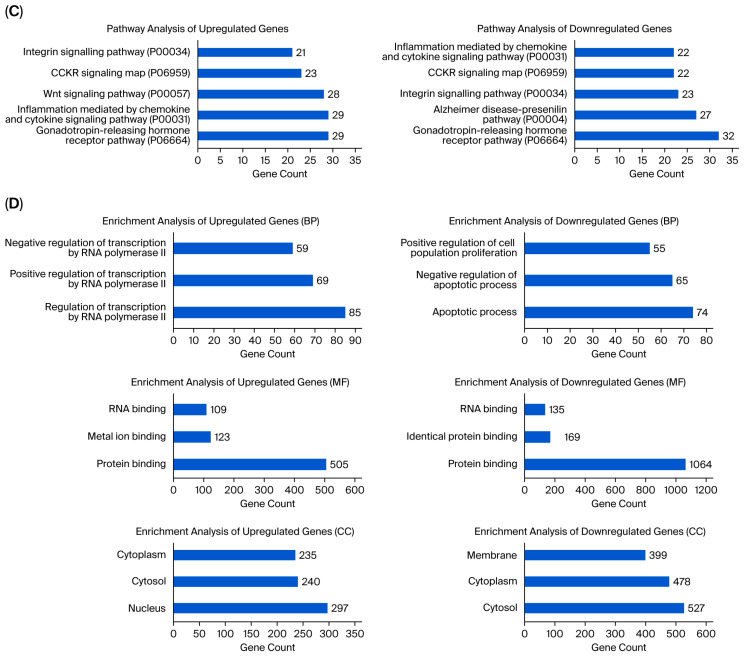
(**A**) Panther pathway and (**B**) DAVID GO enrichment analysis of DEGs between UA_MCF-12A and UA_MDA-MB-231. The ranking was created by selecting the terms with the highest gene count. (**C**) Panther pathway and (**D**) DAVID GO enrichment analysis of upregulated and downregulated DEGs between UA_MCF12A and UA_MDA-MB-231 DEGs. The ranking was created by selecting the terms with the highest gene count (GO: Gene Ontology; KEGG: Kyoto Encyclopedia of Genes and Genomes).

Analysis showed that the greatest number of gene expression differences (4956) were observed between usnic acid-treated breast cancer cells and breast epithelial cells. Furthermore, the application of usnic acid activates different processes in breast cancer cells and breast epithelial cells (Figure 3). The results indicated that the apoptotic processes were mainly suppressed in cancer cells compared to breast epithelial cells upon usnic acid treatment, while transcriptional regulations increased. Examining the DEGs between usnic acid-treated cancer cells and usnic acid-treated breast epithelial cells revealed that the downregulated genes in cancer cells are predominantly located in the cytosol, cytoplasm, and membrane. Together with data obtained from usnic acid-treated cancer cells and the control group, these results suggest that usnic acid application inhibits the membrane and extracellular matrix in cancer cells.

MicroRNAs (miRNAs) play an essential role in cancer. In a previous study, 66 miRNAs were found to be differentially expressed in response to usnic acid in MDA-MB-231 breast cancer cells [8]. An integrated analysis was performed to determine whether there is a correlation between miRNome and transcriptome studies. The analysis showed that almost all of the usnic acid-regulated, differentially expressed miRNA targets (97.4%) overlapped with the gene library analyzed in this study (Figure S4). This indicates that usnic acid controls cellular processes involving specific miRNAs in MDA-MB-231 breast cancer cells.

The expression levels of the genes were significantly altered in the MDA-MB-231 and usnic acid-treated MDA-MB-231 breast cancer cells. In both omic analyses, the expression of 23 genes was significantly altered in comparisons between MDA-MB-231 and usnic acid-treated MDA-MB-231 breast cancer cells, and all were downregulated in the control sample (Table 2). The functional annotation results indicated that the genes were involved in RNA processing and the negative regulation of cell and smooth muscle cell proliferation processes (Figure S5). Consistent with these findings, the CC term for the transcripts was nucleolus. These results highlight the importance of preventing cell proliferation processes upon usnic acid treatment in cancer cells.

### 2.4. Validation of Transcriptome Analysis Using qRT-PCR

**Figure 4:**
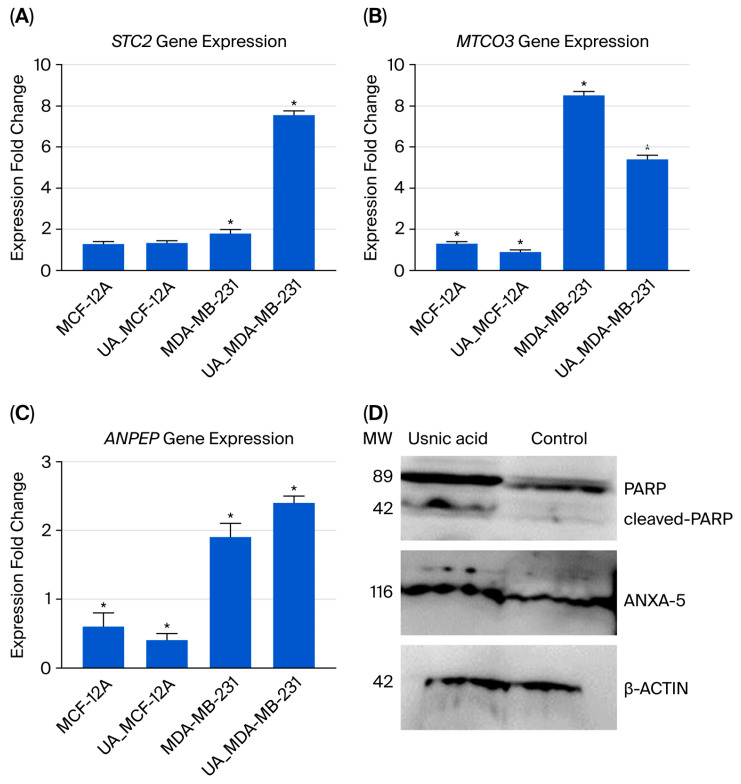
The qRT-PCR analysis results for the selected gene expressions identified in the transcriptome analysis are shown below: (**A**) *STC2* gene expression; (**B**) *MTCO3* gene expression; (**C**) *ANPEP* gene expression; (**D**) Western blot analysis of ANXA5 and PARP proteins in MDA-MB-231 cells treated with usnic acid (22 µM for 48 h). Representative blots show full-length PARP (116 kDa), cleaved PARP (89 kDa), and ANXA5 (36 kDa) protein levels. β-Actin (~42 kDa) was used as the loading control. Densitometric quantification of three independent experiments (n = 3) is presented as the mean ± SD. Treatment with usnic acid increased cleaved PARP by 2.3-fold and ANXA5 by 2.1-fold (*p* < 0.05) compared to the untreated control, indicating the induction of apoptosis in TNBC cells. (* *p* < 0.05).

Three genes were selected for qRT-PCR analysis to validate the transcriptomic data (see Figure 4A–C). The results were comparable in both analyses. Decreased expression of the STC2 gene, which has an anti-hypocalcemic effect on calcium and phosphate homeostasis, is associated with a poor prognosis in breast cancer [16,17]. In usnic acid-treated cancer cells, the STC2 gene expression level increased 3.1-fold according to the transcriptome data, and a 4.2-fold increase was confirmed by the qRT-PCR results compared to the control MDA-MB-231 cells. Decreased mitochondrial DNA gene expression and the disruption of mitochondrial genes have been associated with programmed cell death [18]. Compared to control MDA-MB-231 cells, the MT-CO3 gene expression level decreased 7.1-fold in the transcriptome results and 1.59-fold in the qRT-PCR results in usnic acid-treated MDA-MB-231 cells. The ANPEP gene regulates the epithelial–mesenchymal transition (EMT) pathway and is responsible for apoptosis in breast cancer cell lines [19,20]. In MDA-MB-231 cells treated with usnic acid, ANPEP gene expression increased 2.2-fold in the transcriptome and 1.3-fold in qRT-PCR results compared to the control cells.

### 2.5. Proteome Analysis

To further identify the regulatory network, comparative proteomic analyses were performed on usnic acid-treated breast epithelial cells (MCF-12A) and breast cancer cells (MDA-MB-231), along with their respective controls. The proteome results revealed 93 differentially expressed proteins (DEPs) when comparing usnic acid-treated MCF-12A breast epithelial cells with the control libraries. Of these, almost half were downregulated, while 42 proteins were upregulated in the control breast epithelial cells compared to the usnic acid-treated MCF-12A cells (Table 3,  Tables S2 and S3).

Conversely, usnic acid treatment was found to alter the expression of a greater number of proteins in MDA-MB-231 breast cancer cells. A total of 372 DEPs were found between the libraries of untreated and usnic acid-treated MDA-MB-231 breast cancer cells (Table 3). Among these, 50 proteins were upregulated, while a much higher number of proteins (322) were downregulated in the control MDA-MB-231 breast cancer cells compared to those treated with usnic acid (Table 3).

### 2.6. Functional Enrichment Analysis of the DEPs Between the Libraries

To analyze the effect of usnic acid on MCF-12A breast epithelial cells, ontology and pathway analyses of differentially expressed proteins (DEPs) were performed (Figure S6A). The results revealed that usnic acid treatment mainly affected processes related to chromatin structure in MCF-12A breast epithelial cells. As these processes profoundly affect transcription, the proteome results are consistent with the transcriptome analysis, which showed that usnic acid treatment affects transcription in MCF-12A breast epithelial cells. As in the transcriptome analysis, the most common cellular component (CC) term in the proteome data was nucleus. Glycolysis was found to be the most abundant pathway. A detailed analysis (Figure S6B) showed that the most prevalent biological process in upregulated control cells compared with usnic acid-treated MCF-12A breast epithelial cells was the negative regulation of the apoptotic process, while processes associated with chromatin organization were the most enriched BP terms in downregulated control cells. The most enriched CC terms of both the up- and downregulated transcripts in the control cells also support these results. The most abundant pathways in up- and downregulated control MCF-12A breast epithelial cells were glycolysis and the proteasome, compared with those in usnic acid-treated cancer cells.

**Figure 5:**
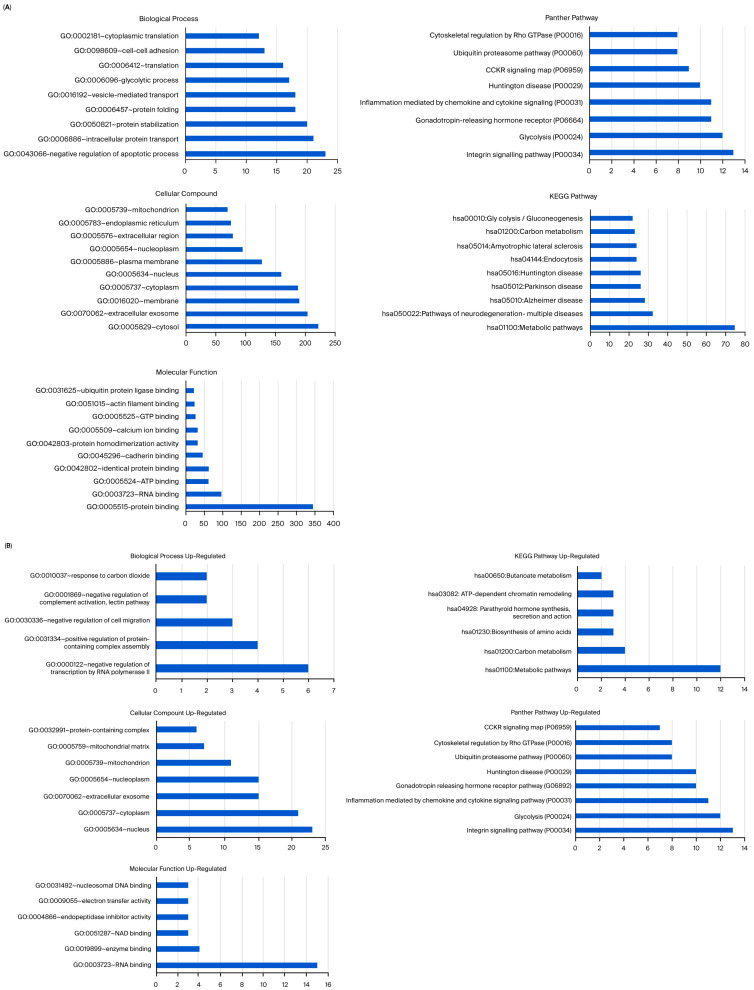

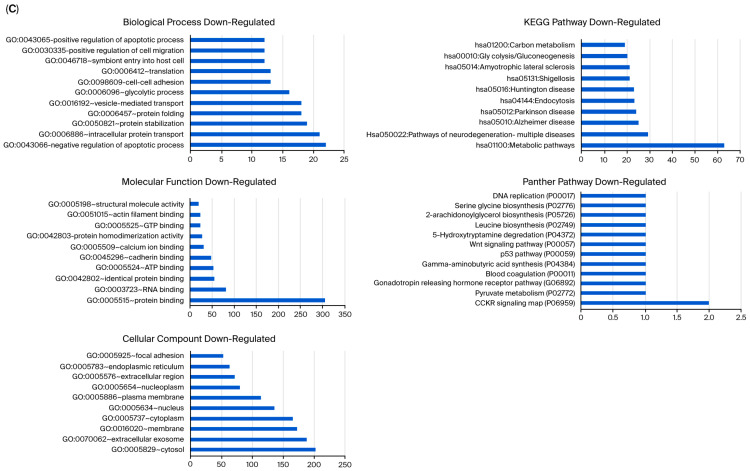
(**A**) GO and KEGG pathway enrichment analyses of DEPs between MDA-MB-231 and UA_MDA-MB-231. (**B**) GO and KEGG pathway enrichment analysis of upregulated and downregulated DEPs between MDA-MB-231 and UA_MDA-MB-231 (UA: usnic acid; GO: Gene Ontology; KEGG: Kyoto Encyclopedia of Genes and Genomes). (C) Downregulated DEPs between MDA-MB-231 and UA_MDA-MB-231 (UA: usnic acid; GO: Gene Ontology; KEGG: Kyoto Encyclopedia of Genes and Genomes).

Unlike MCF-12A breast epithelial cells, usnic acid treatment mostly affected the apoptotic process, protein transport, and protein stabilization in MDA-MB-231 cells (Figure 5A). The biological compartments involved in intercellular communication, such as the cytosol, extracellular exosome, and membrane, were the most enriched CC terms, while the integrin, glycolysis, and gonadotropin pathways were the most abundant PantherDB pathways. These results indicated the effect of usnic acid on cell adhesion and signaling in breast cancer cells, a finding that was also confirmed by transcriptome data. A detailed analysis (Figure 5B) revealed that the most abundant biological process in the upregulated control cells, compared with usnic acid-treated MDA-MB-231 cancer cells, was the negative regulation of the transcription process. Meanwhile, the most enriched biological processes in the downregulated control cells were the negative regulation of apoptosis, intracellular protein transport, and protein stabilization. Consistent with the BP results, the most enriched CC terms were nucleus and cytoplasm for the upregulated MDA-MB-231 cancer cells and cytosol and extracellular exosome for the downregulated control cells. The top two pathways in the upregulated control MDA-MB-231 cells were integrin signaling and glycolysis, whereas the downregulated proteins in the control MDA-MB-231 cancer cells did not show a significant number of pathways.

The STRING tool was used to construct the PPI network of DEPs (Figure S7A). The resulting DEP network for the MDA-MB-231 cancer cells and the control libraries consisted of 370 nodes and 4.353 edges. The PPI network was separated into seven k-means clusters, the descriptions of which are given in Figure S7B. The proteins in the largest cluster (164 genes) were involved in focal adhesion.

### 2.7. Western Blot Analysis

Increased ANXA5 expression has been associated with apoptosis in cells [21,22]. PARP is a protein that plays an active role in DNA repair. Studies have shown that inhibiting PARP is an effective therapeutic strategy for treating triple-negative breast cancer [23]. To validate the proteomic findings, the levels of ANXA5 and PARP proteins were evaluated using Western blot analysis. β-Actin was used as a loading control, and molecular weight markers were included in each blot. Densitometric quantification was performed using ImageJ software (1.54) and normalized to β-actin. Following treatment with usnic acid (22 µM for 48 h), a significant increase in cleaved PARP (~89 kDa fragment) was observed, alongside a corresponding decrease in full-length PARP (~116 kDa), compared to untreated control cells (*p* < 0.05). Densitometric analysis showed that the cleaved PARP/full-length PARP ratio increased 2-fold, indicating activation of PARP cleavage during apoptosis. Consistently, ANXA5 expression increased 2-fold (*p* < 0.05) in treated MDA-MB-231 cells (Figure 4D). These findings confirm that usnic acid triggers apoptotic signaling by promoting PARP proteolytic cleavage and ANXA5 upregulation, in line with our proteomic data and previous studies on PARP-mediated apoptosis in TNBC.

### 2.8. Functional Analysis of Usnic Acid Targets

The caspase gene family is involved in both the intrinsic and extrinsic pathways of apoptosis. The sequential activation of caspases is central to the apoptosis mechanism. The study revealed a significant increase in the expression levels of the *Casp3* and *Casp9* genes (3.2- and 3.5-fold, respectively; *p* < 0.05). Significant increases in the expression levels of *Bax*, another apoptosis-associated gene, were also observed (2.6-fold). Members of the Bcl2 family are involved in the intrinsic pathway and mitochondrial dysfunction. *Bcl2* showed downregulation of gene expression (*p* < 0.05) (Figure S8A).

Flow cytometry analysis revealed that usnic acid induces apoptosis in MDA-MB-231 cells in a concentration-dependent manner. Cells treated with the IC_50_ concentration (22 µM) of usnic acid for 30 h showed a significant shift from the viable (Annexin V^−^/PI^−^) population towards the apoptotic quadrants. The percentage of live cells decreased from 86.2% ± 2.1% in the control group to 66.2% ± 1.9% in the treated group (*p* < 0.05). The percentage of early apoptotic cells increased from 8.3 ± 1.1% to 35.7 ± 2.3%, while the percentage of late apoptotic cells increased from 5.5 ± 0.8% to 16.6 ± 1.7% (*p* < 0.05). Representative dot plots and a gating strategy are shown in Figure S8B. The data represent the mean ± SD of three independent experiments (n = 3). These results demonstrate that usnic acid triggers cell death by activating apoptotic mechanisms in MDA-MB-231 cells in a concentration-dependent manner.

### 2.9. Human Breast Cancer Xenograft Model

**Figure 6:**
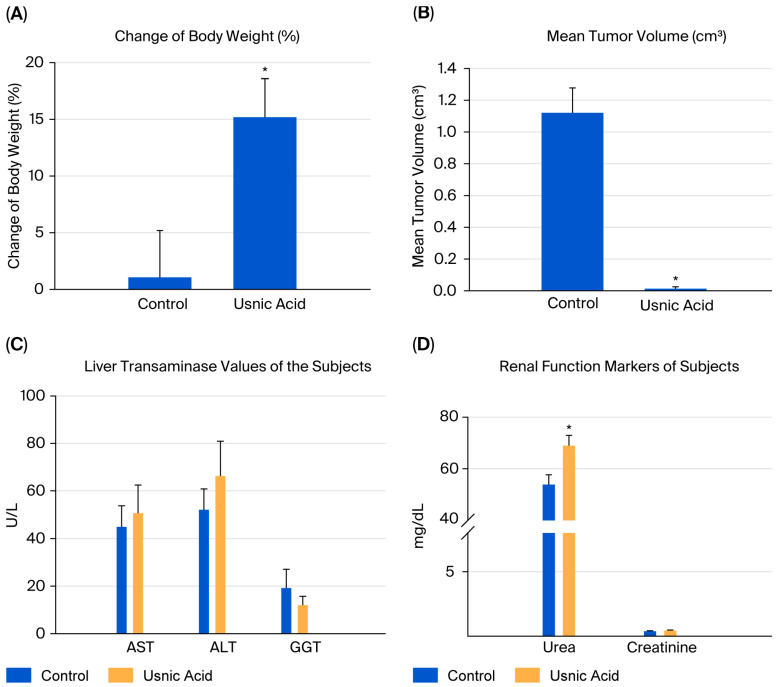
The change in body weight of subjects (n = 6). When the body weights of the experimental groups were compared, it was observed that the trend of an increase was suppressed as the development of breast cancer progressed. (**A**) The difference in body weights was statistically higher in mice in the UA group (15.19% vs. control 1.06%) (*p* = 0.025). (**B**) The mean tumor volume of subjects (n = 6). A statistically significant decrease in tumor growth was detected in athymic nude CD1 mice treated with UA (0.0127 cm^3^) compared to the control group (1.1208 cm^3^) with xenograft breast cancer development (*p* < 0.05). (**C**) The biochemical markers of liver function of the subjects (n = 6). Liver function tests, measured using the serum samples obtained via the centrifugation of blood taken by intracardiac puncture. No difference was found between the control and UA groups in serum aspartate aminotransferase (AST, 44.83 U/L vs. 50.60 U/L), alanine aminotransferase (ALT, 52.00 U/L vs. 66.25 U/L), and gamma-glutamyl transferase (GGT, 19.16 U/L vs. 12.00 U/L) levels, which are markers of liver function (*p* > 0.05). (**D**) The biochemical markers of the renal function of subjects (n = 6). Although there was no difference in serum creatinine levels between the groups, the UA group had significantly higher serum urea levels than the control group (*p* = 0.020). The methods used in the in vivo analyses and all results obtained are presented in Figure S10. (* *p* < 0.05).

**Figure 7:**
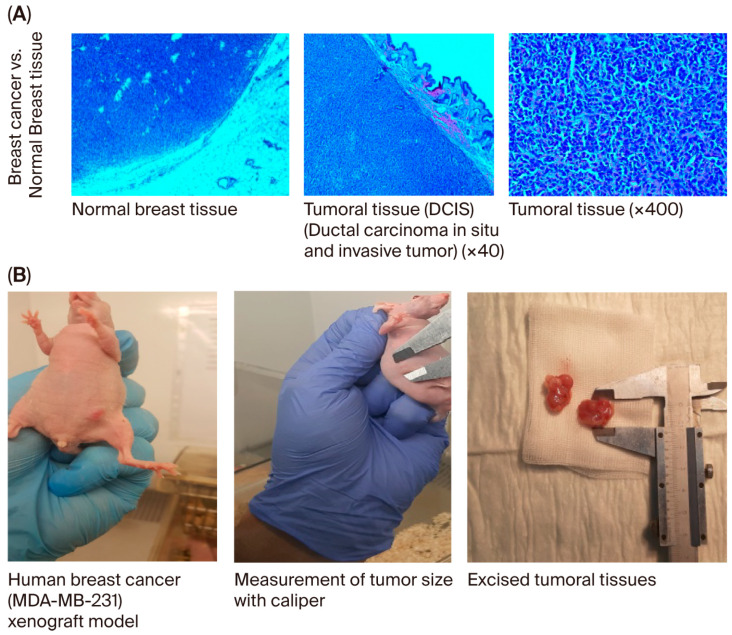
(**A**) Histopathological examination of breast and other tissues. It was detected in pathological sections after the implantation of MDA-MB-231 cancer cells in normal breast tissue. The authorized pathologist evaluated these appearances as ductal carcinoma in situ and invasive ductal carcinoma. (**B**) Evaluation of the growth and metastasis of tumors in the breast cancer group. In the evaluations performed by autopsy, it was found that breast cancer remained limited only in the breast lodge in the abdominal area; in some cases, it progressed under the skin with penetration of subcutaneous muscle and fat tissue but did not cause any distant metastasis.

When the body weights of the experimental groups were compared, it was observed that the rate of increase decreased as breast cancer developed. The difference in body weights was statistically significant in mice in the usnic acid group (*p* = 0.025; Figure 6A). After tumor cells were injected, tumor sizes were measured using digital calipers during the follow-up period, and tumor volume was calculated using the formula volume (cm^3^) = (L (length in cm) × W (width in cm)^2^) × 0.5. A statistically significant decrease in tumor growth was detected in athymic nude CD1 mice treated with usnic acid compared to the control group with xenograft breast cancer development (*p* < 0.05) (Figure 6B and Figure S9, Tables S5–S7). Liver function tests were performed on serum samples obtained by centrifuging blood taken by intracardiac puncture. No difference was found between the control and usnic acid groups in serum aspartate aminotransferase (AST), alanine aminotransferase (ALT), or gamma-glutamyl transferase (GGT) levels, which are markers of liver function (Figure 6C). Although there was no difference in serum creatinine levels between the groups, serum urea levels in the usnic acid group were significantly higher than in the control group (*p* = 0.020) (Figure 6D). Following autopsy, the growth of MDA-MB-231 cancer cells in normal breast tissue was examined by an authorized pathologist using histopathological staining and was evaluated as ductal carcinoma in situ and invasive ductal carcinoma. Additionally, breast cancer was found to remain limited to the breast in the abdominal area. In some cases, it progressed beneath the skin, penetrating subcutaneous muscle and fat, but did not cause distant metastasis (Figure 7A,B). When the body weights of the experimental groups were compared, it was observed that the trend of increase was suppressed as the development of breast cancer progressed. The difference in body weights was statistically higher in mice in the usnic acid group (*p* = 0.025) (Figure 6A). After injection of tumor cells, tumor sizes were measured using digital calipers in the follow-up period, and tumor volume was calculated using the formula (Volume (cm^3^) = (L (length cm) × W (width cm)2) × 0.5, in cm). A statistically significant decrease in tumor growth was detected in athymic nude CD1 mice treated with usnic acid compared to the control group with xenograft breast cancer development (*p* < 0.05) (Figure 6B and Figure S9; Tables S5–S7). Liver function was tested using serum samples obtained by centrifugation of blood taken by intracardiac puncture. No difference was found between the control and usnic acid groups in serum aspartate aminotransferase (AST), alanine aminotransferase (ALT), and gamma-glutamyl transferase (GGT) levels, which are markers of liver function (Figure 6C). Although there was no difference in serum creatinine levels between the groups, the serum urea levels measured in the usnic acid group were significantly higher than in the control group (*p* = 0.020) (Figure 6D). After autopsy, the development of MDA-MB-231 cancer cells settled in normal breast tissue was examined using histopathological staining by the authorized pathologist and evaluated as ductal carcinoma in situ and invasive ductal carcinoma. Additionally, it was found that breast cancer remained limited only in the breast lodge in the abdominal area; in some cases, it progressed under the skin with penetration of subcutaneous muscle and fat tissue but did not cause any distant metastasis (Figure 7A,B).

## 3. Discussion

This study investigated the therapeutic potential of usnic acid on the TNBC cell line MDA-MB-231 using RNA sequencing and proteomic analysis methods. Natural products, which have evolved adaptively in living systems, are well known as potential anti-cancer molecules that often provide greater biological benefits than synthetic molecules. In this study, the therapeutic effect of usnic acid on TNBC was therefore examined in detail at gene and protein expression levels using high-throughput technologies. This study is the most comprehensive in the literature to elucidate the anti-tumor effects of usnic acid on TNBC through transcriptomic and proteomic approaches. The data obtained demonstrate that usnic acid suppresses extracellular matrix remodeling and targets critical cancer-related pathways, including integrin, Rho-GTPase, and angiogenesis. Furthermore, differential gene and protein expression analysis revealed that usnic acid has molecular mechanisms that could potentially inhibit TNBC cell proliferation.

Previous studies have determined that usnic acid exhibits anti-proliferative and apoptotic effects on various cancer cell lines. Studies involving usnic acid have included methods such as MTT assays to assess cytotoxicity, cell proliferation and cell cycle transitions, microRNA microarray analyses, and the determination of apoptotic effects [14,24,25,26,27,28,29].

The effects of usnic acid on human hepatocellular carcinoma (HepG2) and melanoma cells have been investigated, and it has been shown to induce cell death by activating the mitochondrial pathway. However, the molecular mechanisms underlying the therapeutic effects of lichen secondary metabolites on cells are not yet fully understood [30]. For this study, we first analyzed the anti-proliferative effect of usnic acid using the XCELLigence^®^ Real-Time Cell Analysis (RTCA) system. We determined the IC_50_ concentration for the MDA-MB-231 cell line to be 22 µM. Studies in the literature on the anti-cancer effects of EGFR inhibitor drugs related to breast cancer on MDA-MB-231 cells have reported IC_50_ concentrations of 20.7 µM for gefitinib, 42.6 µM for erlotinib, 15.4 µM for carboplatin, 59.6 nM for doxorubicin, and 3.0 nM for docetaxel [31]. As the IC_50_ concentration of usnic acid on the MDA-MB-231 cell line exhibits similar cytotoxic effects to those of the aforementioned chemotherapeutic drugs, it can be concluded that the usnic acid molecule warrants further investigation for therapeutic purposes.

Usnic acid was found to suppress the gonadotropin signaling pathway in MCF-12A normal breast epithelial cells. A molecule’s ability to affect normal breast cells through the gonadotropin pathway suggests it may interfere with cellular mechanisms related to reproductive hormones, thereby altering the hormonal regulation of breast cells. Gonadotropin signaling is associated with cell division and growth. Therefore, if a molecule activates the gonadotropin pathway in normal breast cells, it could lead to faster cell division. However, after usnic acid was applied to normal breast cells, the molecule suppressed gonadotropin signaling, slowing cell growth and proliferation. Unlike conventional colorimetric assays, which provide a static snapshot of viability, XCELLigence^®^ enables the real-time detection of early cytotoxic events and recovery phases. This offers deeper insight into the temporal dynamics of usnic acid-induced cell death. These findings provide significant evidence that usnic acid could potentially halt the process of carcinogenesis.

Transcriptomic and proteomic analyses were conducted to determine the mechanism of action of usnic acid on TNBC cells. The results showed that genes related to integrin and apoptosis processes were suppressed. Suppressing the integrin pathway in MDA-MB-231 cells with the drug candidate molecule may reduce the metastatic potential of cancer cells, disrupt the tumor microenvironment, inhibit cancer growth, induce apoptosis in cancer cells, and enhance treatment efficacy when combined with chemotherapy. Therefore, usnic acid, which suppresses integrin, strongly holds promise as a potential drug candidate in the treatment of aggressive TNBC. The ability of usnic acid to inhibit cell migration and metastasis can be evaluated in advanced preclinical and clinical studies, making it a novel and powerful target in TNBC therapy.

The CCKR (cholecystokinin receptor) pathway is a mechanism that regulates cellular signal transduction through CCK receptors [32]. CCK receptors are predominantly found in the digestive and nervous systems, as well as in certain cancer cells. The modulation of the CCKR pathway affects cell growth, differentiation, metabolism, and hormone secretion [33]. The usnic acid molecule modulates the CCKR pathway in TNBC cells while suppressing tumor growth and metastasis. This indicates that the CCKR pathway suppresses TNBC.

The CCKR pathway shares features with the integrin and apoptosis pathways in triple-negative breast cancer (TNBC). These three pathways have the potential to influence TNBC cell growth, metastasis, and response to treatment [34,35]. The commonality among these pathways lies in their critical roles in TNBC cell survival, migration, invasion, and apoptotic resistance. In light of the data obtained, the usnic acid drug candidate molecule was found to suppress CCKR and integrin signaling while activating the apoptosis pathway. This inhibits the growth and metastasis of TNBC cells. Therefore, usnic acid could be a promising treatment option for TNBC, one of the most aggressive types of breast cancer for which there are currently no effective treatments.

Following the application of usnic acid to TNBC cells, transcriptomic and proteomic analysis identified 16 commonly expressed genes. These genes form interconnected networks that are involved in processes such as proliferation, metastasis, inflammation, and apoptotic resistance in TNBC cells. The insulin-like growth factor (IGF) pathway (*IGFBP3*, *RBM38*, and *KLF10*) is involved in cell growth and proliferation; the NF-κB and inflammation pathway (*TNFAIP3*, *IGFBP3*, and *SNORA23*) is involved in immune evasion and the tumor microenvironment; the integrin–extracellular matrix (ECM) pathway (*SCNN1G*, *IGFBP3*, and *SLC30A1*) is involved in metastasis and invasion; the apoptosis pathway (*RBM38*, *KLF10*, and *TNFAIP3*) is involved in cell death signals and chemotherapy sensitivity; hypoxia and metabolism (*SLC30A1*, *IGFBP3*, and *KLF10*) are involved in tumor growth and resistance mechanisms; and RNA processing and protein production (*SNORA/SNORD* genes) are related to ribosomal adaptation processes for rapid cell proliferation. The expression levels of these genes were observed to be suppressed following treatment with usnic acid, which significantly altered TNBC cell growth, metastasis, and treatment response. The network generated by the Phyton analysis tool illustrates the relationships among these genes and their interactions with critical biological processes, including IGF, NF-κB, apoptosis, integrin–ECM, hypoxia, and RNA processing pathways, following usnic acid treatment.

In our previous laboratory study, we determined the miRNA profile following usnic acid treatment across different breast cancer subtypes (BT-474, MCF-7, MDA-MB-231) [8]. The genes (*ADGRE5*, *ANXA6*, *APMAP*, *CALU*, *CD44*, *CKAP4*, *HSD17B11*, *ICAM1*, *ITGA3*, *ITGB1*, *MBOAT7*, *NCEH1*, and *PCYOX1*) identified in the transcriptomic and proteomic data after the application of usnic acid to TNBC cells are of particular interest when considering the relationship with miRNAs (hsa-miR-8485, hsa-miR-6807-5p, hsa-miR-3171, hsa-miR-6805-5p, etc.) identified in our previous study. Understanding the relationship between these genes and miRNAs will help us explore how post-transcriptional gene regulation occurs. MiRNAs play a role in post-transcriptional regulation by binding to specific gene mRNAs and either inhibiting gene expression or leading to post-transcriptional modifications.

Certain microRNAs (miRNAs) can bind to the mRNAs of specific genes, thereby preventing protein production. For example, hsa-mir-21-5p regulates genes associated with cancer and cell adhesion. miRNAs such as hsa-mir-196a-5p and hsa-mir-29a-5p regulate the expression of cell adhesion molecules, including CD44, ICAM1, and ITGA3, and may therefore be associated with cell migration and cancer metastasis [36,37]. Following the application of usnic acid to MDA-MB-231 cells, hsa-mir-21-5p targets genes that promote cell proliferation and inhibits apoptosis pathways. In contrast, hsa-mir-26a-5p and hsa-mir-26b-5p generally target proteins that control the cell cycle and regulate apoptosis pathways [38]. Cell–matrix interactions are usually regulated by cell adhesion molecules and integrins. The expression of genes such as *CD44*, *ITGA3*, *ITGB1*, and *ICAM1* plays an important role in these processes. MiRNAs control the expression of these genes, thereby altering cell–matrix interactions [39]. MiRNAs such as hsa-mir-196a-5p and hsa-mir-1275 can regulate the expression of genes involved in cell–matrix interactions and cell motility. These miRNAs typically target genes that influence cell migration and metastasis [40]. Some of these genes, especially *HSD17B11*, *MBOAT7*, *PCYOX1*, and *NCEH1*, are involved in lipid metabolism and steroid biosynthesis. The regulation of these pathways affects metabolic diseases and lipid homeostasis. MiRNAs such as hsa-mir-374a-5p, hsa-mir-1290, and hsa-mir-484 influence genes that regulate lipid metabolism and energy homeostasis. Genes such as *ICAM1*, *ADGRE5*, and *CD44* regulate immune responses and cell adhesion. miRNAs such as hsa-mir-19b-3p and hsa-mir-21-5p, however, inhibit the expression of these genes. *CD44*, *ITGA3*, and *ITGB1* regulate cell–matrix interactions [41]. MicroRNAs (miRNAs) such as hsa-mir-196a-5p and hsa-mir-196b-5p can influence the expression of these genes, while hsa-mir-8485 targets the *ADGRE5* gene to regulate interactions with immune cells, hsa-mir-26b-5p targets the *ICAM1* gene to affect cell adhesion and immune responses, and hsa-mir-6807-5p targets the *CD44* gene to potentially influence cancer development and cellular mobility.

Following the application of usnic acid to TNBC cells, transcriptomic and proteomic analyses revealed pathways associated with CCKR, integrin signaling, and apoptosis. To validate invasion and metastasis potential, the gene expression levels of *MMP9*, *TWIST1*, *ITGB1*, and *ICAM1* were analyzed using qRT-PCR. The results showed that usnic acid significantly downregulated the expression of these genes in TNBC cells, thereby inhibiting their invasion and metastasis capabilities. Similarly, the expression profiles of apoptosis-associated genes such as *Bcl-2*, *Caspase-3*, *Caspase-9*, and *Bax* were analyzed, and apoptosis was assessed using flow cytometry. The results confirmed that usnic acid induced apoptosis at a high rate in TNBC cells by downregulating anti-apoptotic genes and upregulating pro-apoptotic genes. One of the proteins selected for Western blot analysis was Annexin 5 (ANXA5), which was included in the proteome data. ANXA5, a phospholipase A2 and protein kinase C inhibitor with calcium channel activity, is a cell membrane protein involved in the detection of apoptotic cells [42,43]. The results from our LC/MS-MS proteomic data and Western blot analysis showed that ANXA-5 protein expression, an indicator of cell apoptosis, increased with usnic acid treatment. Another protein identified in the Western blot study was PARP, an important apoptotic marker in MDA-MB-231 cells. Existing studies have determined that PARP inhibitor molecules have a therapeutic effect in MDA-MB-231 cells [44,45]. According to the results of the Western blot analysis, PARP cleavage increased 2.3-fold in cells treated with usnic acid. PARP plays an active role in important biological processes, such as transcription, cell cycle regulation, response to DNA damage, apoptosis, and maintenance of genome integrity. The presence of cleaved PARP is one of the most widely used biomarkers for detecting apoptosis [44,45]. It has been determined that PARP proteolysis following usnic acid treatment creates a response to an apoptotic stimulus. In a study by Jakimov et al. on the MDA-MB-231 cell line, decreased PARP protein expression due to degradation was reported to induce apoptosis independently of caspases [44,45,46]. The results show that usnic acid induces apoptosis by stimulating PARP and inhibiting DNA repair.

This study, which was conducted on athymic nude CD1 female mice created as a xenograft mammalian cancer model, is an exemplary study in which micro-exposure was applied at the cellular level. The issues of poor aqueous solubility and low bioavailability of usnic acid, as well as concerns about its hepatotoxicity, have been frequently noted in the literature. Several cases of drug-induced liver injury in humans have been attributed to usnic acid, with proposed mechanisms involving uncoupling of mitochondrial oxidative phosphorylation and subsequent hepatocyte damage [47]. In vitro studies on mouse hepatocytes demonstrate that usnic acid can induce necrosis, oxidative stress, and mitochondrial dysfunction [48]. Reviews emphasize that, despite its promising bioactivities, its practical application is hindered by its very limited water solubility and the difficulty of achieving systemic exposure [49,50]. Various formulation strategies have been proposed to overcome these limitations, such as nanoencapsulation, phospholipid complexes, and the conversion of usnic acid into more soluble salts or nanosuspensions, to improve absorption and reduce toxicity [51]. Guided by this information, our main goal in this study was to prove the direct cellular effect of usnic acid on target cells (e.g., a cancer cell line or another cell type) under controlled conditions rather than modeling full in vivo pharmacokinetics or systemic exposure. Using a direct dosing paradigm (e.g., adding usnic acid dissolved in a compatible solvent, potentially with minimal excipients) allowed us to bypass absorption barriers and first-pass metabolism, thereby maximizing the chance of observing a biological effect within the constraints of an in vitro setting.

The planned experimental procedure involved fully exposing breast cancer cells to the IC_50_ concentration of usnic acid and then monitoring changes in tumor development. Usnic acid appears to exhibit significant tumor-suppressing properties at these concentrations compared to the control group. These findings were evaluated alongside studies on the potential of usnic acid and its derivatives in different cancer types in the existing literature. Xenograft models have been utilized in many preclinical assays to evaluate the efficacy of various anti-cancer agents [52], investigate metabolic transformation in breast cancer, and identify potential therapeutic interventions [53]. A study by Pyrczak-Felczykowska et al. (2022) shows that isoxazole derivatives of usnic acid trigger the endoplasmic reticulum (ER) stress response in MCF-7 breast cancer cells, leading to paraptosis-like cell death [54]. This suggests that usnic acid may destroy cancer cells via alternative cell death pathways. However, Ebrahim et al. (2017) reported that benzylidene analogues of usnic acid exhibited potent anti-cancer properties by inhibiting the mammalian target of the rapamycin (mTOR) pathway in breast cancer cells [55]. This mechanism suggests that usnic acid may be effective in suppressing cellular growth and proliferation processes. Similarly, Song et al. (2012) showed that usnic acid suppresses tumor angiogenesis and growth by inhibiting the VEGFR2-mediated AKT and ERK1/2 signaling pathways [56]. These results suggest that usnic acid supports the anti-cancer effect by inhibiting the formation of vessels that nourish cancer cells. The promising effects of usnic acid on cancer development have been demonstrated in studies of not only breast cancer, but also hepatocellular [57], pancreatic [58], lung [59], colorectal [60], and gastric [61] cancers.

In our study, the levels of AST, ALT, and GGT, as well as serum creatinine, remained within the normal range across all groups. This indicates that there was no evidence of hepatic or renal dysfunction associated with exposure to usnic acid. Unfortunately, serum electrolytes (Na^+^, K^+^, and Cl^−^) were not measured, which is a limitation of the study. Despite the beneficial effects observed with usnic acid treatment, serum urea levels increased. These levels can be influenced by various preanalytical and physiological factors, such as fasting duration, time of blood sampling, dehydration status, and protein catabolism [62,63]. In our experiment, all blood samples were collected at a consistent time point under non-fasting conditions to minimize circadian or fasting-related variability. In the absence of corresponding changes in creatinine or liver enzyme levels, the isolated elevation in serum urea is most likely an incidental or transient metabolic finding rather than an indication of systemic toxicity. This interpretation is consistent with the existing toxicological literature. Chronic oral or dietary exposure to usnic acid has been associated with hepatotoxicity and, in some reports, mild renal biochemical alterations due to systemic absorption [64]. However, in topical or localized applications, usnic acid is primarily associated with local tissue responses (e.g., modulation of inflammation and wound healing) rather than significant systemic organ toxicity [65,66]. Therefore, given the non-systemic route of administration in our study, the elevation in transient urea likely reflects physiological rather than toxicological variation. Further studies should investigate whether the increase in urea levels is related to usnic acid.

A limitation of our study is that the breast cancer cells were exposed to usnic acid prior to being implanted. In such a context, issues of poor solubility and bioavailability that would otherwise limit in vivo efficacy are less significant because the exposure is direct and localized. In other words, our experimental setup enables us to ‘force’ exposure to an effective concentration to test our hypotheses and cellular responsiveness. As our study is not intended to be a final in vivo therapeutic formulation, we consider this to be a justified first step. If the observed responses are robust, they may then guide the development of an optimized formulation for in vivo translation.

Our study provided evidence that the development of triple-negative breast cancer with MDA-MB-231 cancer cells in a xenograft breast cancer model can be suppressed by usnic acid. Based on the existing literature, promising future development strategies include nanoencapsulation (e.g., PLGA-, liposome-, or polymeric nanoparticle systems) to enhance solubility, stability, and controlled release, and potentially reduce off-target hepatic exposure [50]. Converting usnic acid into more soluble derivatives or salts (e.g., usnate salts) has been proposed to increase aqueous solubility and bioavailability, potentially reducing hepatotoxicity [51]. Designing prodrugs or co-formulations with permeation enhancers, micellar systems, or lipid-based carriers could help to further overcome absorption barriers. This study contributes to ongoing efforts to develop novel and effective treatments for breast cancer based on natural compounds such as usnic acid.

Although several studies have linked usnic acid to mitochondrial damage and apoptosis induction across various cancer types, this study is the first to combine transcriptomic and proteomic data to identify molecular responses specific to TNBC. Notably, our findings reveal that usnic acid impacts gonadotropin, CCKR, and lipid/energy metabolism pathways. These pathways had not previously been associated with usnic acid’s mechanism of action. Consequently, our research sheds new light on the molecular basis of usnic acid’s tumor-suppressive effects in TNBC, highlighting potential therapeutic targets that could inform the development of new drugs or combination therapies.

In conclusion, our study provided evidence that the development of triple-negative breast cancer using MDA-MB-231 cancer cells in a xenograft breast cancer model can be suppressed by usnic acid. This study contributes to ongoing efforts to develop novel, effective breast cancer treatments based on natural compounds such as usnic acid. However, further research is needed to fully elucidate the mechanism of action, optimize dosing strategies, assess potential toxicity profiles, and explore efficacy across diverse TNBC models, including patient-derived xenografts and metastatic settings. Additionally, combination studies with existing chemotherapeutics or targeted agents could offer valuable insights into its potential for translation into clinical practice. Overall, our results provide a strong foundation for the continued preclinical development of usnic acid towards future clinical evaluation.

## 4. Materials and Methods

### 4.1. Cell Culture

The MDA-MB-231 breast cancer cells and the MCF-12A normal epithelial breast cells were obtained from the American Type Culture Collection (ATCC). The MDA-MB-231 cells were cultured in Dulbecco’s Modified Eagle’s Medium (DMEM) (Sigma, Kanagawa, Japan) containing 1% L-glutamine (1 mM), 10% fetal bovine serum (FBS) (Biological Industries), and 1% penicillin/streptomycin (Biowest, Bradenton, FL, USA). The MCF-12A cells were cultured in DMEM/F12 (Sigma) supplemented with 10% FBS (Biological Industries), 1% penicillin/streptomycin (Biowest), 10 µg/mL insulin (Sigma), 20 ng/mL EGF (Goquick), and 0.5 mg/mL hydrocortisone (PubChem). The cells were cultured at 37 °C in a 5% CO_2_ incubator.

### 4.2. The Determination of Anti-Proliferative Effect of Usnic Acid by XCELLigence^®^ Analysis

Commercial usnic acid (Sigma-Aldrich, St. Louis, MI, USA) was used. A 100 mM stock solution of usnic acid was prepared in DMEM (Biowest) containing 0.05% DMSO (Sigma-Aldrich), and the anti-proliferative effect was determined by preparing usnic acid at different concentrations (100, 50, 25, 12.5, 6.25, 3.125, and 1.562 µM).

The effect of different concentrations of usnic acid on cells at various time intervals was monitored using the XCELLigence^®^ RTCA S16 real-time cell analysis system (Roche Applied Science, Acea Biosciences, San Diego, CA, USA). The device determines the cell index (CI), which expresses the number or morphological changes in the cells, by taking measurements on plates containing microelectronic sensor arrays that measure the impedance between cells and sensors. Background measurements were taken by adding 100 µL of DMEM to the e-plate (Acea Biosciences, RTCA, Roche, Penzberg, Germany). A total of 1 × 10^5^ cells were seeded on the e-plate and incubated for 30 min at room temperature. Cell proliferation was then monitored for 24 h, after which the usnic acid concentrations (100 µM, 50 µM, 25 µM, 12.5 µM, 6.25 µM, 3.125 µM, and 1.562 µM) were applied to the wells. The time- and dose-dependent effects of usnic acid were monitored by measuring the change in electrical impedance in the wells over 120 h.

The cell index (CI) graph and IC_50_ values were calculated using the XCELLigence^®^ RTCA software program (RTCA, ACEA Biosciences, Roche, Germany). The 50% inhibitory concentration (IC_50_) was calculated after 48 h using nonlinear regression and a four-parameter logistic (4PL) sigmoidal dose–response model (least squares fit) in the RTCA software. IC_50_ concentrations are expressed as the mean ± standard deviation (SD) with 95% confidence intervals (CI 95%). Goodness-of-fit (R^2^) values were >0.97 for all analyses. Statistical comparisons between groups were performed using two-way ANOVA, followed by Tukey’s post hoc test (GraphPad Prism 9), with *p* < 0.05 being considered significant.

### 4.3. Transcriptome Analyses

#### 4.3.1. Total RNA Extraction

Both cell lines were seeded in 6-well plates at a concentration of 5 × 10^5^ cells per well. Usnic acid was then applied to the cells at the IC_50_ concentration for 48 h. Cells without usnic acid were used as a control for each cell line. Total RNA was isolated from the cells in accordance with the TRIZOL method. Protein and DNA residues were then removed from the RNA samples using an SV RNA Isolation Kit (Promega, Madison, WI, USA). The quality of the isolated RNA was measured using a Nanodrop ND-1000 Spectrophotometer (Thermo Fisher Scientific, Waltham, MA, USA) and visualized using 1.3% agarose gel electrophoresis. For each transcriptome study sample, three biological replicates were pooled into a single tube by combining equal amounts of RNA, which was then prepared for sequencing.

#### 4.3.2. RNA Sequence Analysis

RNA samples (non-treated and treated with usnic acid; MCF-12A and MDA-MB-231) were sequenced using the Illumina HiSeq 2000 platform according to the manufacturer’s protocol. The RNA samples were used at a concentration of 300 ng per library, and the mRNA was purified using poly-T oligo-linked magnetic beads. NEBNext First Strand Synthesis Reaction Buffer (5×) was used for fragmentation. First-strand cDNA was synthesized using M-MuLV Reverse Transcriptase (RNase H-) and random hexamer primers. DNA Polymerase RNase H was then used to synthesize the second strand of cDNA. The remaining overhangs were converted into blunt ends using exonuclease and polymerase activities. The 3′ end DNA fragments were then adenylated, after which NEBNext adapters were ligated for hybridization. The cDNA fragments, 150–200 bp in length, were purified using the AMPure XP system. The adapter-linked cDNAs were then treated with 3 μL of USER enzyme at 37 °C for 15 min, followed by heating to 95 °C for 5 min prior to the polymerase chain reaction (PCR). Phusion High-Fidelity DNA Polymerase, Universal PCR Primers, and Index (X) Primer were used for the PCR. The PCR products were then purified using the AMPure XP system. Index-coded samples were clustered on a cBot Clustering System with the TruSeq PE Cluster Kit v3-cBot-HS (Illumina). The prepared sample libraries were sequenced on the Illumina HiSeq 2000 platform. This generated paired-end reads of 150 bp, with an average of 30 million reads obtained per sample.

#### 4.3.3. Transcriptome Data Analysis

The RNA sequence reads were analyzed using FastQC [16,17] for quality control. The adapter sequences were trimmed using the FASTX-Toolkit [67]. After quality control, reads with a Phred quality score of at least 30 and a minimum length of 75 bp were retained for downstream analysis. The filtered reads were then aligned to the human reference genome (Homo sapiens.GRCh38.dna) using the BWA-MEM algorithm. Multi-mapped reads were discarded, and uniquely mapped reads were used for quantification [68]. Multi-mapped reads were discarded, and uniquely mapped reads were used for quantification. The aligned reads were then analyzed for differential gene expression using StringTie [69]. As the biological replicates were pooled prior to sequencing, the differential expression was reported as ‘descriptive’ and assessed based on log_2_-fold change. Transcripts showing log_2_FC ≥ 1.5 or ≤−1.5 were considered differentially expressed genes (DEGs). For heatmap visualization, hierarchical clustering was performed using the complete linkage method and the Euclidean distance metric, as implemented in the pheatmap R package 1.0.13 [70]. Gene expression values (log_2_-transformed FPKM) were normalized across samples by calculating the z-score for each gene. Specifically, the mean expression across all samples was subtracted from each gene, and the result was divided by the standard deviation so that the mean was 0 and the standard deviation was 1 for each gene.

The resulting z-scores were visualized in heatmaps, with hierarchical clustering performed using the Euclidean distance metric and the complete linkage method. The transcriptome data were deposited in GeneBank under accession number PRJNA1114661. The SRA accession numbers for the libraries belonging to the BioProject are as follows: SRR29125092 for MCF-12A; SRR29125091 for MCF-12A-UA; SRR291125090 for MDA-MB-231; and SRR29125089 for MDA-MB-231-UA.

#### 4.3.4. Comparison of miRNA Microarray Data and Transcriptome Data

Cansaran-Duman et al. [8] used microarray analysis to determine the miRNA profile formed after usnic acid application in MDA-MB-231 triple-negative breast cancer cells. A total of 66 miRNAs and target genes were identified in usnic acid-treated MDA-MB-231 breast cancer cells. The miRWalk2.0 web-based analysis tool was used to identify the target genes of the miRNAs [71]. KEGG, GOBP, PANTHER Pathway, and Wiki-VTM data analyses on the miRWalk2.0 website revealed pathway and target gene analysis results for 24 of the 66 usnic acid-specific miRNAs, with the miRNA target genes matching the transcriptome library genes.

#### 4.3.5. qRT-PCR Analysis

A total of three genes, selected from the transcriptome assay, were used for validation. The primer list can be found in Table S4, and qRT-PCR analysis was performed using the SYBR Green I (5X Hot Firepol Eva Green qPCR Mix Plus) protocol. The glyceraldehyde-3-phosphate dehydrogenase (GAPDH) gene was used as the standard. The reaction conditions for analysis were as follows: 95 °C for 12 min, followed by 15 cycles of 95 °C for 15 s and 60 °C for 60 s, and then 40 cycles of 95 °C for 15 s and 60 °C for 60 s, completed with the LightCycler 480 Real-Time PCR Instrument (Roche, Germany). Gene expression was analyzed using the 2^ΔΔCt^ method [72].

### 4.4. Proteome Analysis

#### 4.4.1. Protein Isolation

For both cell lines (MDA-MB-231 and MCF-12A), 5 × 10^5^ cells per well were seeded in 6-well plates. The cells were then treated with usnic acid at the IC_50_ concentration for 48 h, with untreated cells serving as controls. Following incubation, the cells were washed with cold PBS (Gibco) and collected by centrifugation at 12,000 rpm for 14 min at 4 °C. The supernatant was then discarded, and the cell pellet was washed twice with PBS before being lysed using 300 µL of 1× Cell Lysis Buffer (Cell Signaling Technology, Danvers, MA, USA), which contained protease and phosphatase inhibitors. The lysates were then vortexed and centrifuged at 14,000× *g* for 10 min at 4 °C. The resulting supernatants were stored at −20 °C until required.

#### 4.4.2. LC-MS/MS Analysis

Whole-cell lysates from control and usnic acid-treated cells were analyzed using shotgun LC–MS/MS proteomics. Rather than in-gel digestion, protein digestion was performed using the filter-aided sample preparation (FASP) method. In brief, the proteins were reduced and alkylated with 50 mM iodoacetamide in the dark for 20 min, then digested overnight with sequencing-grade trypsin at a ratio of 1:100 at 37 °C. The resulting peptides were desalted and reconstituted in 0.1% formic acid prior to LC–MS/MS analysis.

LC–MS/MS measurements were performed on an ACQUITY UPLC M-Class system coupled to a SYNAPT G2-XS QT of mass spectrometer (Waters, Milford, MA, USA). The peptides were first trapped on a Symmetry C18 trap column (5 µm, 180 µm × 20 mm) and then separated on an analytical CSH C18 column (1.7 µm, 75 µm × 250 mm) using a 3–40% acetonitrile gradient containing 0.1% formic acid at a flow rate of 300 nL/min. The instrument was operated in positive ion mode using SONAR data-independent acquisition (DIA), with a quadrupole transmission window of 24 Da, dynamic exclusion for 20 s, and an m/z range of 50–1950.

Raw data were processed using Progenesis QI for Proteomics (v2.0, Waters) and the UniProt Homo sapiens database (release 2022_01). Carbamidomethylation (C) was set as the fixed modification, and oxidation (M) and deamidation (N/Q) were set as the variable modifications. Protein and peptide identification were filtered at a false discovery rate (FDR) of 1%. Label-free quantification (LFQ) was performed using normalized total ion intensity. Proteins were considered to be differentially expressed if they met the following criteria: q < 0.05, ≥2 unique peptides, and a fold change of ≥1.5.

#### 4.4.3. Proteome Data Analysis

Statistical evaluation of differential expression was performed in Progenesis QI. Proteins with log2 FC ≥ 1.5 or log2 FC ≤ −1.5 and an adjusted *p*-value (q-value) < 0.05 were classified as differentially expressed proteins (DEPs), where the q-value represents the FDR-adjusted *p*-value (Tables S2 and S3). PANTHERdb and DAVID Bioinformatics Resources were used for pathway and enrichment analyses, with a *p*-value of < 0.05. The STRING database was used to predict protein–protein associations [73]. The interaction score was set to ≥0.4, and protein–protein interaction networks were produced.

#### 4.4.4. Western Blot

To confirm the proteome results, MDA-MB-231 cells were treated with the IC50 concentration of usnic acid and without usnic acid and then analyzed by Western blot. The protein lysates were boiled in a dry block heater (Major Science, Saratoga, CA, USA) with a loading buffer for 5 min. A total of 30 micrograms of protein from each sample was separated by electrophoresis at 100 V for 90 min using a 12% sodium dodecyl sulphate polyacrylamide gel (SDS-PAGE). A semi-dry transfer system (Cleaver, UK) was then used to transfer the resolved proteins onto polyvinylidene fluoride (PVDF) membranes (Thermo Fisher Scientific, USA). A blocking buffer containing 5% *w*/*v* non-fat dry milk was prepared in Tris-buffered saline containing 0.1% *v*/*v* Tween-20 (TBST). The membranes were then incubated in the blocking buffer for 2 h at 37 °C. The membranes were then incubated with ANXA5 and PARP protein antibodies (1:2000) (Cell Signaling Technology, MA, USA) overnight at 4 °C. Mouse anti-β-actin secondary antibodies (1:1000) (Cell Signaling Technology, MA, USA) were used as an internal control. The membranes were washed four times for 10 min in TBST at room temperature. The washed membranes were then incubated with a horseradish peroxidase (HRP)-conjugated anti-mouse IgG antibody (1:2000) in PBS for 90 min (Cell Signaling Technology, USA). The membranes were washed four times for 10 min at room temperature after the incubation. The membranes were then treated with a chemiluminescence reagent (SuperSignal West Pico Chemiluminescent Substrate, Thermo Fisher Scientific, USA) according to the manufacturer’s protocol. The Licor Imaging System (LI-COR Odyssey Fc 2800, USA) was used to capture the protein blotting images. The relative expression rate of the antibodies was determined by calculating the ratio of the integrated optical density (IOD) of the primary antibody to that of β-Actin.

### 4.5. Functional Analysis

Validation of the transcriptome analysis and the apoptosis pathway by gene expression analysis.

RNA was isolated from usnic acid-treated and control samples using the TRIZOL method, and cDNA was isolated from the RNA to determine the expression levels of the *STC2*, *MT-CO3*, and *ANPEP* genes. GAPDH was used as the housekeeping gene. The primer sequences are given in Table S4.

### 4.6. Flow Cytometry Analysis

A total of 5 × 10^5^ cells were seeded on a 6-well plate. For apoptosis analysis, the cells were incubated for 24 h. The incubated cells were then treated with the IC_50_ concentration of usnic acid. The cells were then incubated for a further 30 h, after which the MDA-MB-231 breast cancer cells were collected and washed with PBS. The cells were stained with 5 µL of Apotracker Green (BioLegend, San Diego, CA, USA), followed by 10 µL of propidium iodide (PI) (BioLegend, USA), and then incubated for 20 min at room temperature in the dark. Apoptotic cells after the incubation period were determined using a CytoFLEX flow cytometer (Roche, Germany). At least 10,000 total events were recorded for each sample. The gating strategy involved first excluding debris using forward scatter (FSC) and side scatter (SSC) and then separating the live (Annexin V^−^/PI^−^), early apoptotic (Annexin V^+^/PI^−^), late apoptotic (Annexin V^+^/PI^+^), and necrotic (Annexin V^−^/PI^+^) populations. Unstained, Apotracker Green-only and PI-only single-stained controls were used to set compensation and define quadrant boundaries. The data were analyzed using CytExpert software (v2.3). The percentages of live (Apotracker^−^/PI^−^), early apoptotic (Apotracker^+^/PI^−^), late apoptotic (Apotracker^+^/PI^+^), and necrotic (Apotracker^−^/PI^+^) cells were calculated. All experiments were performed in biological triplicate (n = 3). Statistical analyses were performed using one-way ANOVA followed by Tukey’s post hoc test, and the results are presented as mean ± SD. Differences with *p* < 0.05 were considered statistically significant.

### 4.7. Human Breast Cancer Xenograft Model

Five-week-old CD1 athymic/nude female mice (from Charles River Laboratories) were used in a study of a human breast cancer xenograft model. The mice were kept in specific-pathogen-free (SPF) cages of five per cage, under a 12 h light/dark cycle, and were fed ad libitum. The mice were randomly divided into two groups of six experimental animals (Figure S10: Graphical abstract of in vivo assay).

MDA-MB-231 cells (2 × 10^4^) in 0.5 mL control solvent (DMSO (14 μM)) (Group I), or test solutions containing 5 μg/mL Matrigel (BD Biosciences) (Group II) (usnic acid (22 µM)), were injected subcutaneously into the abdominal mammary fat pad (n = 6 mice). Follow-up was performed twice weekly, and body weight changes were expressed as a percentage of the difference between the initial and final measurements. Tumor sizes were measured using digital calipers, and tumor volume (V) was calculated using the following formula: Volume (mm^3^) = (length (mm) × width (mm)^2^) × 0.5.

The study was completed at the end of the 30-day period. The subjects were euthanized using the intracardiac blood collection method under anesthesia, and the tumors were excised at the same time. Blood samples were centrifuged and stored for biochemical studies. In addition to tumor tissues, heart, liver, lung, and kidney tissues were fixed in 10% buffered formalin phosphate solution and prepared for pathological examination. The tissues were properly prepared by pathologists, then embedded in paraffin and sectioned. The tissues were stained with hematoxylin and eosin and examined for metastasis under a light microscope.

The SigmaPlot 11 (GMBH, Germany) program was used to analyze the data. The significance level was set at *p* < 0.05. The data distribution was tested using the Shapiro–Wilk test. Student’s *t*-test was used for the pairwise comparison of normally distributed groups; otherwise, the Mann–Whitney U-test was used for the others.

## 5. Conclusions

This study is the first to provide a comprehensive understanding of the molecular mechanism of usnic acid in TNBC cells through transcriptomic and proteomic analyses and the identification of potential targets. Usnic acid suppresses extracellular matrix remodeling and targets critical cancer-related pathways, including the integrin, gonadotropin, CCKR, Rho-GTPase, and angiogenesis pathways. It has the potential to reduce the metastatic capacity of cancer cells, disrupt the tumor microenvironment, inhibit cancer growth, induce cancer cell apoptosis, and enhance the efficacy of chemotherapy. Therefore, usnic acid, which suppresses integrins, strongly holds promise as a potential drug candidate in the treatment of aggressive TNBC. Although usnic acid showed significant tumor-suppressive potential compared to the control at the concentrations used in the in vivo experiment, this design does not model systemic pharmacokinetics or chronic exposure. Testing the ability of usnic acid to inhibit cell migration and metastasis in vitro and in vivo can evaluate its potential as a novel, powerful target in TNBC therapy through advanced preclinical and clinical studies.

## Figures and Tables

**Table 1:** Number of genes differentially expressed in MDA-MB-231 and MCF-12A cells formed by usnic acid application in four different libraries after RNA sequencing analysis (UA: usnic acid; DEG: differentially expressed genes).

Library	DEG	Genes (Upregulated)	Genes (Downregulated)
MCF-12A vs. UA_MCF-12A	1563	513	1050
MDA-MB-231 vs. UA_MDA-MB-231	974	201	773
MCF12A vs. MDA-MB-231	4007	2283	1724
UA_MCF-12A vs. UA_MDA-MB-231	4956	2741	2215

**Table 2:** Downregulated DEGs in MDA-MB-231 compared to usnic acid (UA)-treated MDA-MB-231.

	MCF-12A/UA_MCF-12A	MCF-12A/MDA-MB-231	MDA-MB-231/UA_MDA-MB-231	UA_MCF-12A/UA_MDA-MB-231
AC055811.2	−1	−0.4	−1.8	−1.2
AL136038.4	0.1	0.8	−1.6	−1
AL357520.1	−1	0.2	−1.7	−0.4
AL590369.1	−0.6	0.2	−1.8	−1.1
HIST1H2BD	−0.3	0.7	−2.2	−1.3
IGFBP3	−1.2	0.1	−2.1	−0.8
IKZF5	−1.2	0.4	−1.6	0
KLF10	−1.1	−0.4	−1.7	−0.9
MED4-AS1	−1	0	−2.1	−1.1
NTF3	−1.4	0.7	−1.9	0.2
PIP4P1	−1.4	−0.7	−1.6	−0.8
RBM38	−0.9	0.5	−1.5	−0.1
SCNN1G	−0.9	0.2	−1.5	−0.5
SLC30A1	−0.8	1.2	−1.5	0.6
SNORA16A	0.3	1.3	−2	−1.1
SNORA19	−0.4	0.2	−1.6	−1
SNORA23	−0.5	0	−1.5	−1
SNORD14D	0.2	1.1	−1.9	−0.9
SNORD43	−0.1	0.6	−1.9	−1.1
SNORD57	−0.6	0.8	−1.6	−0.2
SNORD94	0.4	0.8	−1.7	−1.3
SNORD97	−0.6	0	−1.7	−1.1
TNFAIP3	−0.9	1.2	−2	0.1

**Table 3:** Peptide and protein numbers obtained using LC-MS/MS analysis (DEP: differently expressed protein; UA: usnic acid).

LC-MS/MS Analysis	MCF-12A vs. UA_MCF-12A Library	MDA-MB-231 vs. UA_MDA-MB-231 Library
Peptide numbers	17,307	11,897
Protein numbers	1625	1369
DEPs (*p* ≤ 0.05, log2 FC ≥ 1.5, log2 FC ≤ −1.5	93	372
Number of upregulated proteins (UA-non-treated vs. UA-treated cells)	42	50
Number of downregulated proteins (UA-non-treated vs. UA-treated cells)	51	322

## Data Availability

The transcriptome data was available in GeneBank with the accession number PRJNA1114661. The processed proteomic data required to reproduce the results are fully available within the article and its Supplementary Materials. Additional details can be obtained from the corresponding authors upon reasonable request.

## Supplementary Materials

The following supporting information can be downloaded at https://www.mdpi.com/article/10.3390/molecules30214281/s1, Figure S1: (A) GO and KEGG pathway enrichment analyses of DEGs between MCF-12A and UA_MCF-12A. The ranking was created by selecting the terms with the highest gene count. (B) GO and KEGG pathway enrichment analyses of upregulated and downregulated DEGs between MCF-12A and UA_MCF-12A. The ranking was created by selecting the terms with the highest gene count. DAVID GO terms (UA: usnic acid; GO: Gene Ontology, KEGG: Kyoto Encyclopedia of Genes and Genomes); Figure S2: (A) GO and KEGG pathway enrichment analysis of DEGs between MDA-MB-231 and UA_MDA-MB-231. The ranking was created by selecting the terms with the highest gene count. (B) GO and KEGG pathway enrichment analyses of upregulated and downregulated DEGs between MDA-MB-231 and UA_MDA-MB-231. The ranking was created by selecting the terms with the highest gene count (UA: usnic acid; GO: Gene Ontology, KEGG: Kyoto Encyclopedia of Genes and Genomes); Figure S3: (A) GO and KEGG pathway enrichment analysis of DEGs between MCF12A and MDA-MB-231. The ranking was created by selecting the terms with the highest gene count. (B) GO and KEGG pathway enrichment analyses of upregulated or downregulated DEGs between MCF-12A and MDA-MB-231. The ranking was created by selecting the terms with the highest gene count (UA: usnic acid; GO: Gene Ontology, KEGG: Kyoto Encyclopedia of Genes and Genomes); Figure S4: Numbers of transcripts and miRNA targets. A total of 61.058 transcripts were identified in transcriptome analysis, while 3.596 miRNA targets were identified in miRome analysis. A total of 3.505 transcripts overlapped between transcripts and miRNA targets; Figure S5: Functional annotation of the DEGs obtained from comparison of MDA-MB-231 and usnic acid-treated MDA-MB-231 breast cancer cells; Figure S6: GO and KEGG pathway enrichment analysis for MCF-12A and UA_MCF-12A proteins (UA: usnic acid; GO: Gene Ontology, KEGG: Kyoto Encyclopedia of Genes and Genomes); Figure S7: (A) STRING interaction analysis result DEPs between MCF-12A and UA_MCF-12A libraries. (B) K-means descriptions; Figure S8: Flow cytometry analysis of apoptosis induced by usnic acid in MDA-MB-231 cells. MDA-MB-231 cells were treated with the IC_50_ concentration of usnic acid (22 µM) for 30 h, followed by staining with Apotracker Green and propidium iodide (PI). Representative dot plots are shown for (A) unstained control, (B) single-stained controls (Apotracker Green or PI only), (C) untreated control, and (D) usnic acid-treated cells. Quadrant distribution indicates live cells (lower left, Apotracker^−^/PI^−^), early apoptotic cells (lower right, Apotracker^+^/PI^−^), and late apoptotic/necrotic cells (upper right, Apotracker^+^ late apoptotic/necrotic cells (uApotracker^−^/PI^+^). Gating was performed using unstained and single-stained controls for compensation, and a minimum of 10,000 events per sample was acquired. (E) Quantification of live, early apoptotic, and late apoptotic cell populations is shown as mean ± SD from three independent experiments (n = 3). Statistical significance was determined by one-way ANOVA, followed by Tukey’s post hoc test (* *p* < 0.05, ** *p* < 0.01). Usnic acid significantly reduced the viable cell population and increased both early and late apoptotic fractions in a concentration-dependent manner; Figure S9: The average for tumor growth in the control and UA groups over time per mouse with mean ± SEM. (n = 6); Figure S10: Graphical abstract of in vivo assay. Table S1: RNA sequencing data summary; Table S2: Proteom data of MCF-12A vs. MCF-12A-UA; Table S3: Proteom data of MDA-MB-231 vs. MDA-MB-231-UA; Table S4: Primer sequence information of identified genes for real-time PCR analysis; Table S5: Tumor sizes were measured using digital calipers, and tumor volume (v) was calculated using the following formula: volume (cm^3^) = (L(length cm) × W(width cm)^2^) × 0.5; Table S6: Tumor sizes were measured using digital calipers, and the raw individual tumor length and width measurements for each mouse are presented according to time (C: control; U: usnic acid); Table S7: Tumor sizes were measured using digital calipers, and tumor volume (v) was calculated using the following formula: volume (cm^3^) = (L(length cm) × W(width cm)^2^) × 0.5.
