# Supplementary material for: Comprehensive Evaluation of Usnic Acid as a Potential Drug Candidate for Triple-Negative Breast Cancer: Insights from Transcriptomic, Proteomic, and In Vivo Analyses
Source: Molecules. 2025 Nov 4;30(21):4281. doi: 10.3390/molecules30214281 (PMC12610760; doi:10.3390/molecules30214281)
Supplement: Supplementary file 1 [file molecules-30-04281-s001.zip › molecules-3903326-supplementary.pdf]

Supplementary Figures

Figure S1

A

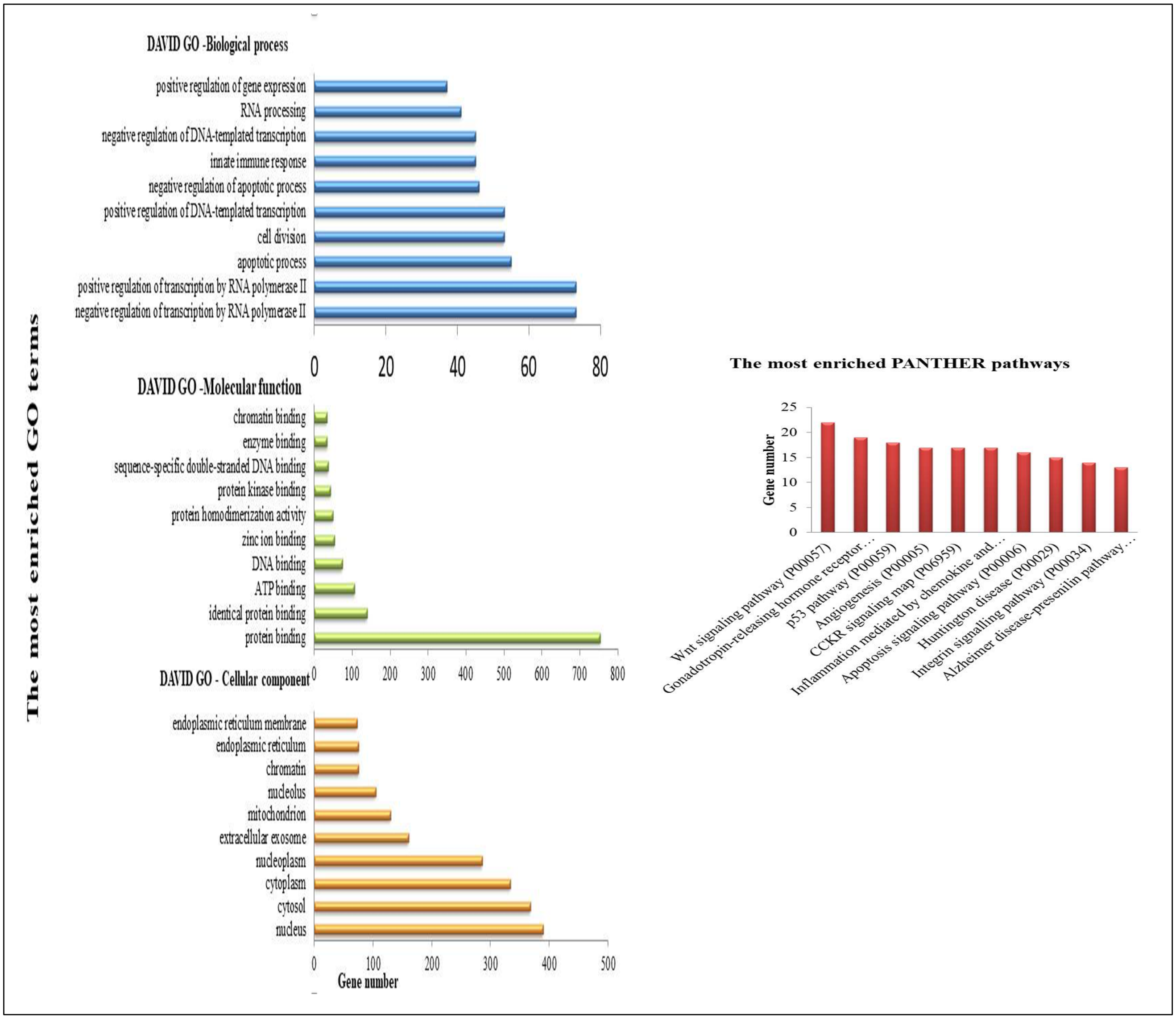

Figure S1.

B

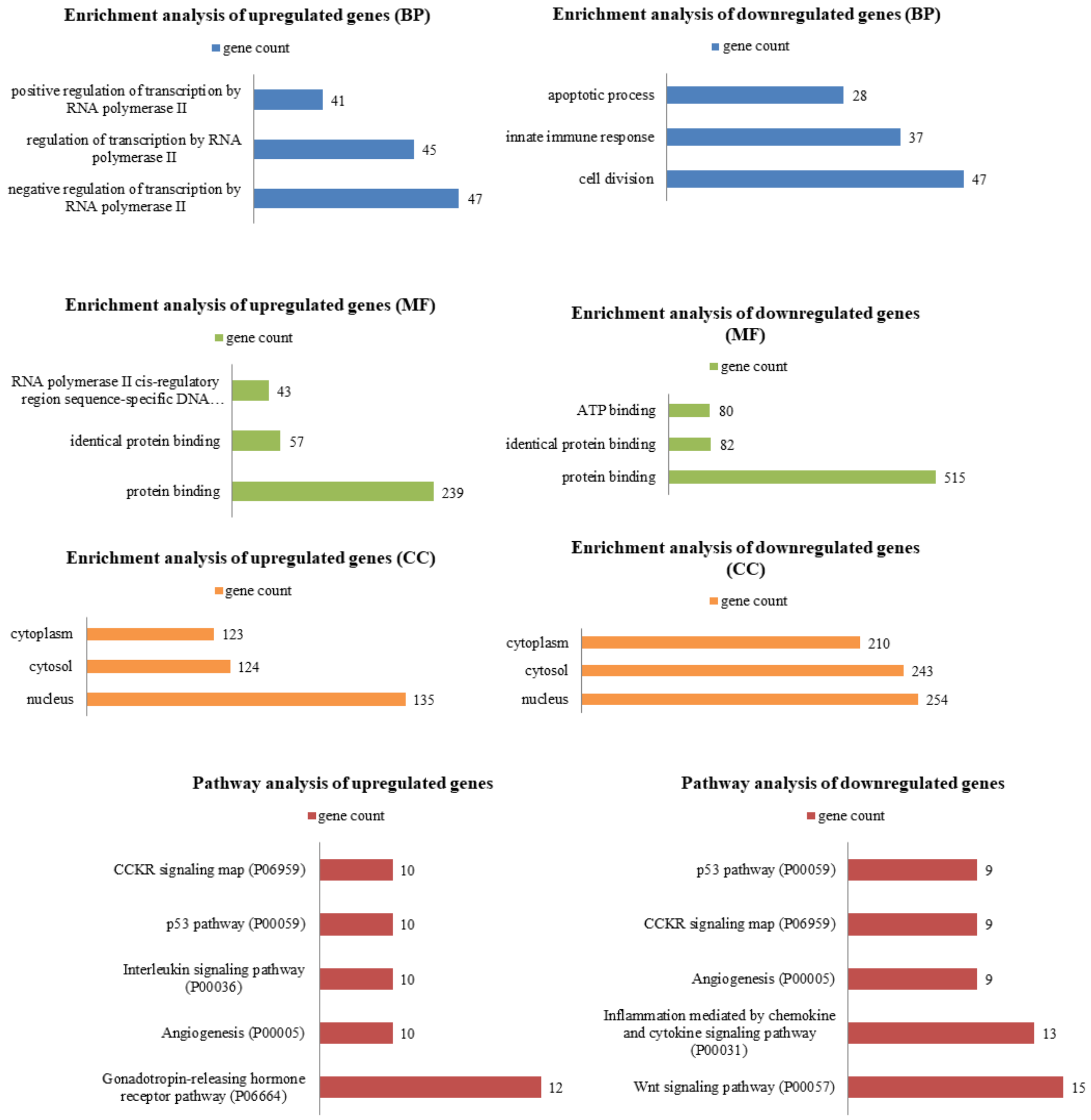

Enrichment analysis of upregulated genes (BP)
gene count
positive regulation of transcription by RNA polymerase II
41
regulation of transcription by RNA polymerase II
45
negative regulation of transcription by RNA polymerase II
47
Enrichment analysis of downregulated genes (BP)
gene count
apoptotic process
28
innate immune response
37
cell division
47
Enrichment analysis of upregulated genes (MF)
gene count
RNA polymerase II cis-regulatory region sequence-specific DNA…
43
identical protein binding
57
protein binding
239
Enrichment analysis of downregulated genes (MF)
gene count
ATP binding
80
identical protein binding
82
protein binding
515
Enrichment analysis of upregulated genes (CC)
gene count
cytoplasm
123
cytosol
124
nucleus
135
Enrichment analysis of downregulated genes (CC)
gene count
cytoplasm
210
cytosol
243
nucleus
254
Pathway analysis of upregulated genes
gene count
CCKR signaling map (P06959)
10
p53 pathway (P00059)
10
Interleukin signaling pathway (P00036)
10
Angiogenesis (P00005)
10
Gonadotropin-releasing hormone receptor pathway (P06664)
12
Pathway analysis of downregulated genes
gene count
p53 pathway (P00059)
9
CCKR signaling map (P06959)
9
Angiogenesis (P00005)
9
Inflammation mediated by chemokine and cytokine signaling pathway (P00031)
13
Wnt signaling pathway (P00057)
15

Figure S2.

A

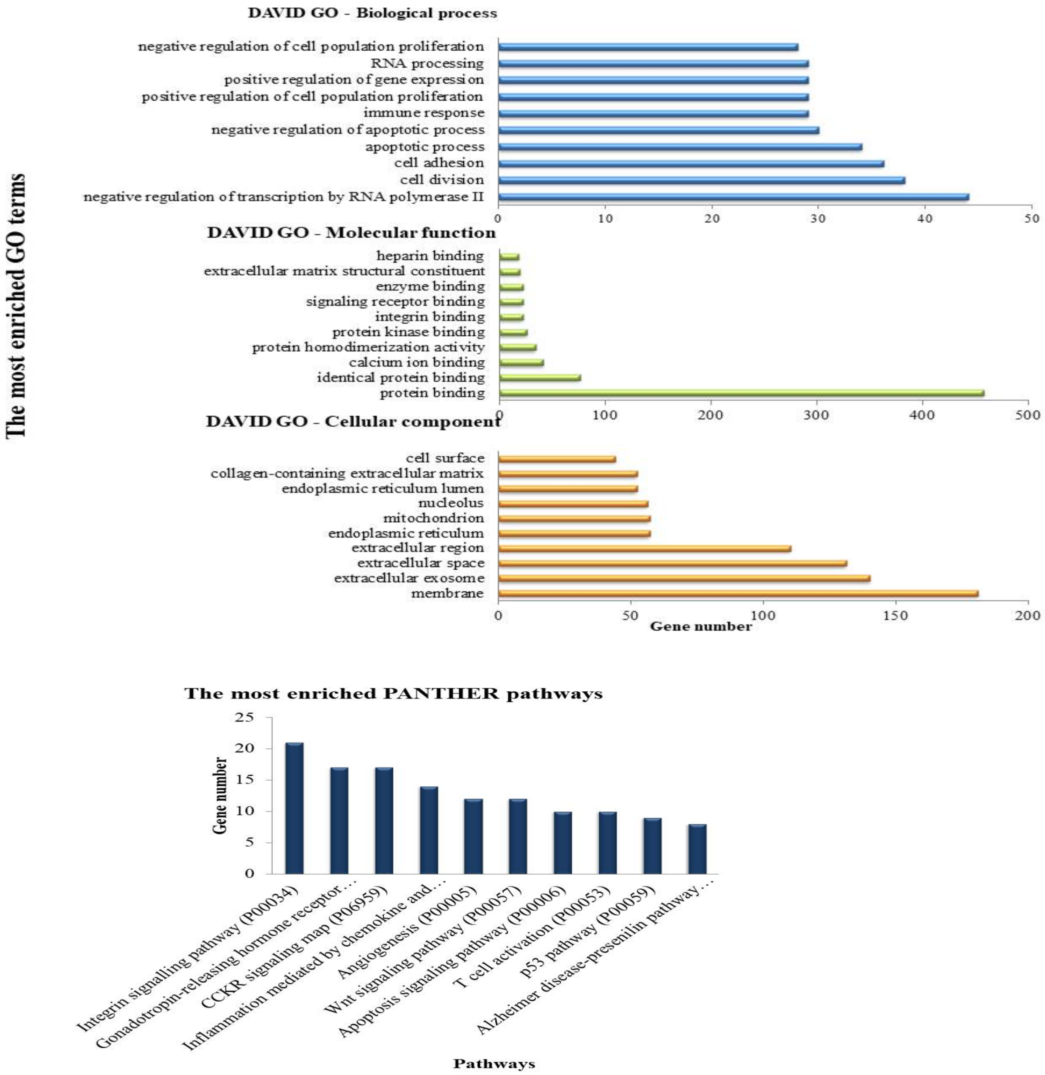
DAVID GO - Biological process
negative regulation of cell population proliferation
RNA processing
positive regulation of gene expression
positive regulation of cell population proliferation
immune response
negative regulation of apoptotic process
apoptotic process
cell adhesion
cell division
negative regulation of transcription by RNA polymerase II
0 10 20 30 40 50
DAVID GO - Molecular function
heparin binding
extracellular matrix structural constituent
enzyme binding
signaling receptor binding
integrin binding
protein kinase binding
protein homodimerization activity
calcium ion binding
identical protein binding
protein binding
0 100 200 300 400 500
DAVID GO - Cellular component
cell surface
collagen-containing extracellular matrix
endoplasmic reticulum lumen
nucleolus
mitochondrion
endoplasmic reticulum
extracellular region
extracellular space
extracellular exosome
membrane
0 50 100 150 200
Gene number
The most enriched GO terms
The most enriched PANTHER pathways
0 5 10 15 20 25
Gene number
Integrin signalling pathway (P00034)
Gonadotropin-releasing hormone receptor…
CCKR signaling map (P06959)
Inflammation mediated by chemokine and…
Angiogenesis (P00005)
Wnt signaling pathway (P00057)
Apoptosis signaling pathway (P00006)
T cell activation (P00053)
p53 pathway (P00059)
Alzheimer disease-presenilin pathway…
Pathways

Figure S2.

B

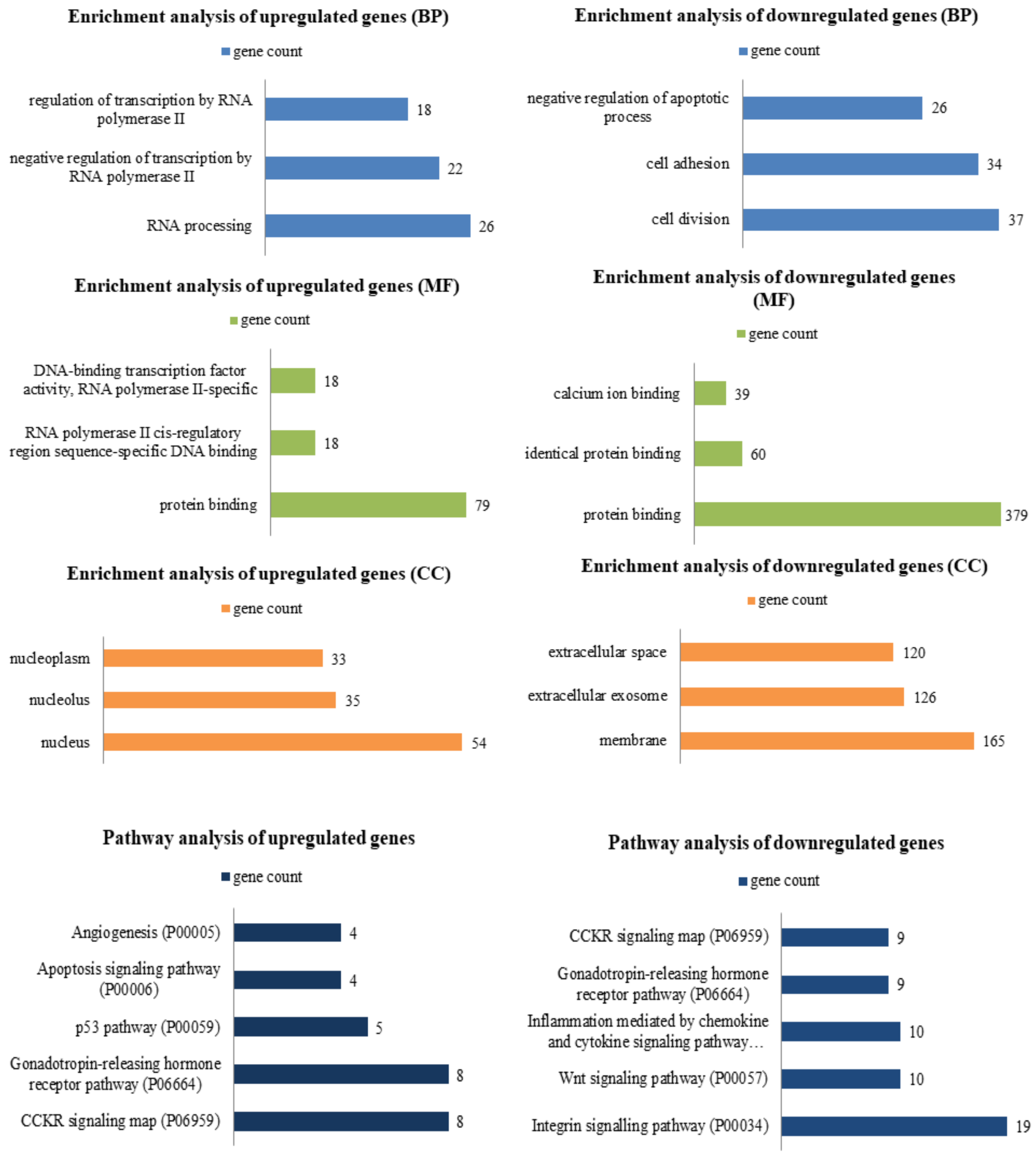
Enrichment analysis of upregulated genes (BP)
gene count
regulation of transcription by RNA polymerase II
18
negative regulation of transcription by RNA polymerase II
22
RNA processing
26
Enrichment analysis of downregulated genes (BP)
gene count
negative regulation of apoptotic process
26
cell adhesion
34
cell division
37
Enrichment analysis of upregulated genes (MF)
gene count
DNA-binding transcription factor activity, RNA polymerase II-specific
18
RNA polymerase II cis-regulatory region sequence-specific DNA binding
18
protein binding
79
Enrichment analysis of downregulated genes (MF)
gene count
calcium ion binding
39
identical protein binding
60
protein binding
379
Enrichment analysis of upregulated genes (CC)
gene count
nucleoplasm
33
nucleolus
35
nucleus
54
Enrichment analysis of downregulated genes (CC)
gene count
extracellular space
120
extracellular exosome
126
membrane
165
Pathway analysis of upregulated genes
gene count
Angiogenesis (P00005)
4
Apoptosis signaling pathway (P00006)
4
p53 pathway (P00059)
5
Gonadotropin-releasing hormone receptor pathway (P06664)
8
CCKR signaling map (P06959)
8
Pathway analysis of downregulated genes
gene count
CCKR signaling map (P06959)
9
Gonadotropin-releasing hormone receptor pathway (P06664)
9
Inflammation mediated by chemokine and cytokine signaling pathway…
10
Wnt signaling pathway (P00057)
10
Integrin signalling pathway (P00034)
19

Figure S3.

A

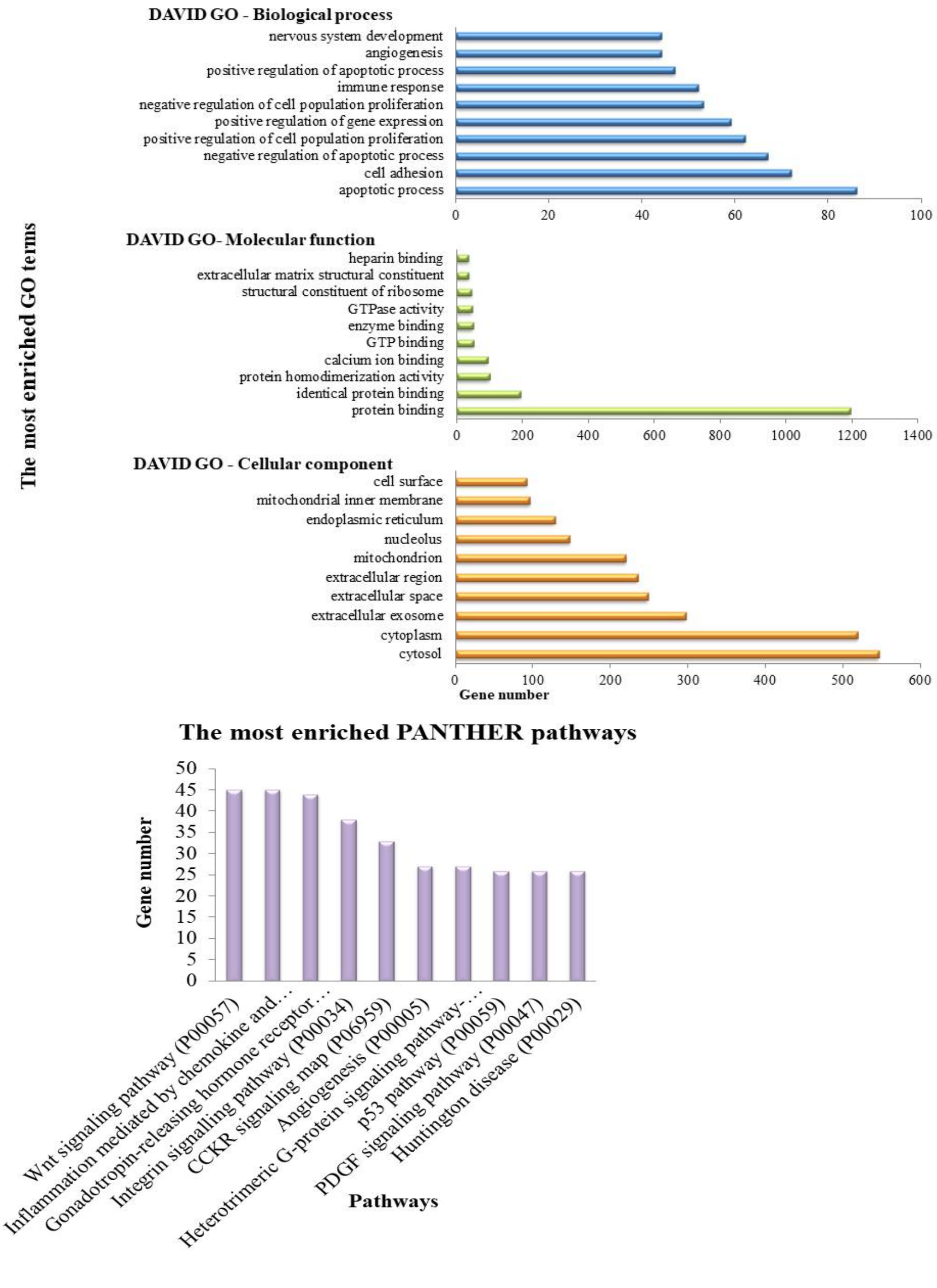

DAVID GO - Biological process
nervous system development
angiogenesis
positive regulation of apoptotic process
immune response
negative regulation of cell population proliferation
positive regulation of gene expression
positive regulation of cell population proliferation
negative regulation of apoptotic process
cell adhesion
apoptotic process
0 20 40 60 80 100
DAVID GO- Molecular function
heparin binding
extracellular matrix structural constituent
structural constituent of ribosome
GTPase activity
enzyme binding
GTP binding
calcium ion binding
protein homodimerization activity
identical protein binding
protein binding
0 200 400 600 800 1000 1200 1400
DAVID GO - Cellular component
cell surface
mitochondrial inner membrane
endoplasmic reticulum
nucleolus
mitochondrion
extracellular region
extracellular space
extracellular exosome
cytoplasm
cytosol
0 100 200 300 400 500 600
Gene number
The most enriched GO terms
The most enriched PANTHER pathways
Gene number
50 45 40 35 30 25 20 15 10 5 0
Wnt signaling pathway (P00057)
Inflammation mediated by chemokine and…
Gonadotropin-releasing hormone receptor…
Integrin signalling pathway (P00034)
CCKR signaling map (P06959)
Angiogenesis (P00005)
Heterotrimeric G-protein signaling pathway-…
p53 pathway (P00059)
PDGF signaling pathway (P00047)
Huntington disease (P00029)
Pathways

Figure S3.

B

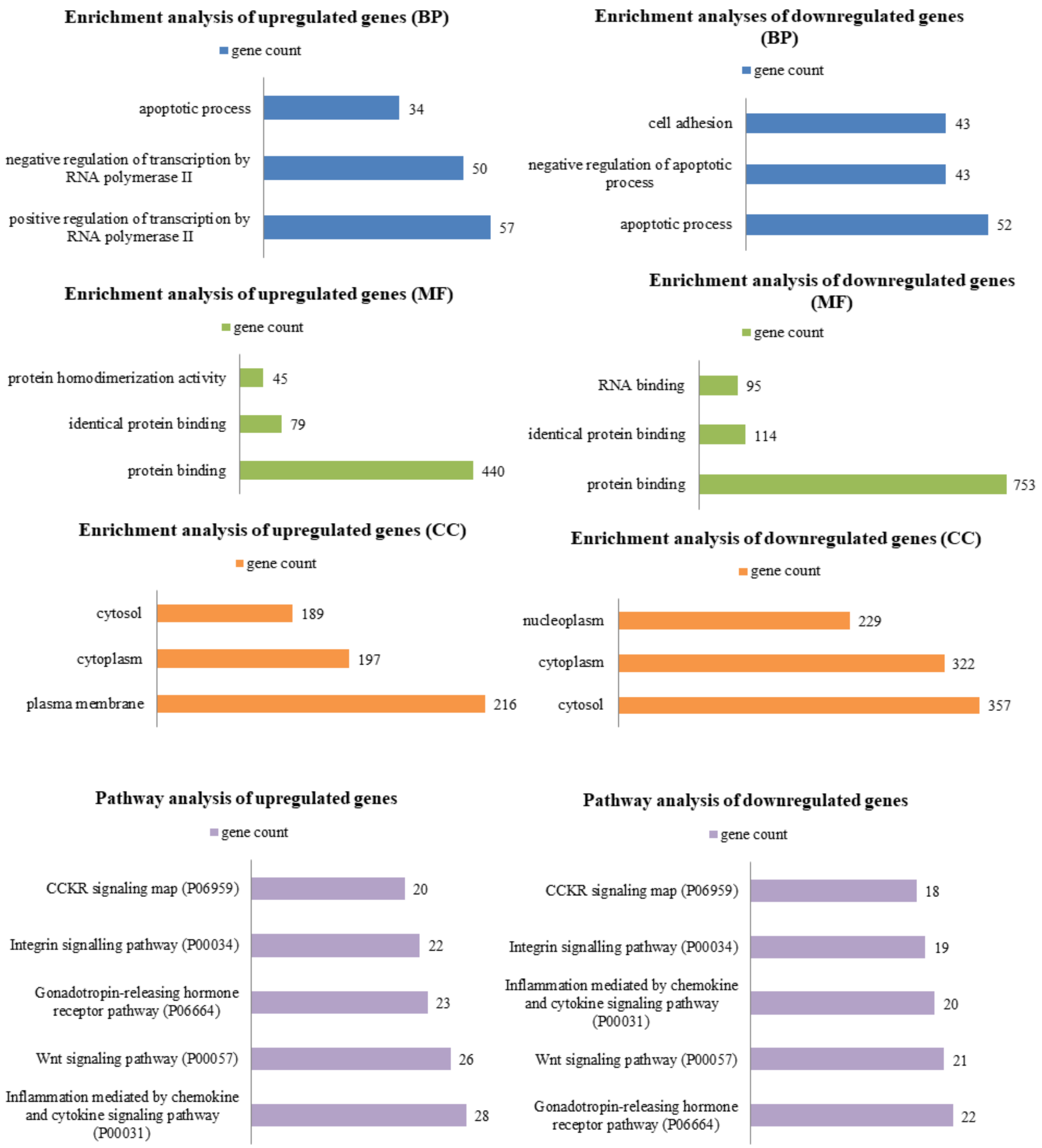
Enrichment analysis of upregulated genes (BP)
gene count
apoptotic process
34
negative regulation of transcription by RNA polymerase II
50
positive regulation of transcription by RNA polymerase II
57
Enrichment analyses of downregulated genes (BP)
gene count
cell adhesion
43
negative regulation of apoptotic process
43
apoptotic process
52
Enrichment analysis of upregulated genes (MF)
gene count
protein homodimerization activity
45
identical protein binding
79
protein binding
440
Enrichment analysis of downregulated genes (MF)
gene count
RNA binding
95
identical protein binding
114
protein binding
753
Enrichment analysis of upregulated genes (CC)
gene count
cytosol
189
cytoplasm
197
plasma membrane
216
Enrichment analysis of downregulated genes (CC)
gene count
nucleoplasm
229
cytoplasm
322
cytosol
357
Pathway analysis of upregulated genes
gene count
CCKR signaling map (P06959)
20
Integrin signalling pathway (P00034)
22
Gonadotropin-releasing hormone receptor pathway (P06664)
23
Wnt signaling pathway (P00057)
26
Inflammation mediated by chemokine and cytokine signaling pathway (P00031)
28
Pathway analysis of downregulated genes
gene count
CCKR signaling map (P06959)
18
Integrin signalling pathway (P00034)
19
Inflammation mediated by chemokine and cytokine signaling pathway (P00031)
20
Wnt signaling pathway (P00057)
21
Gonadotropin-releasing hormone receptor pathway (P06664)
22

Figure S4.

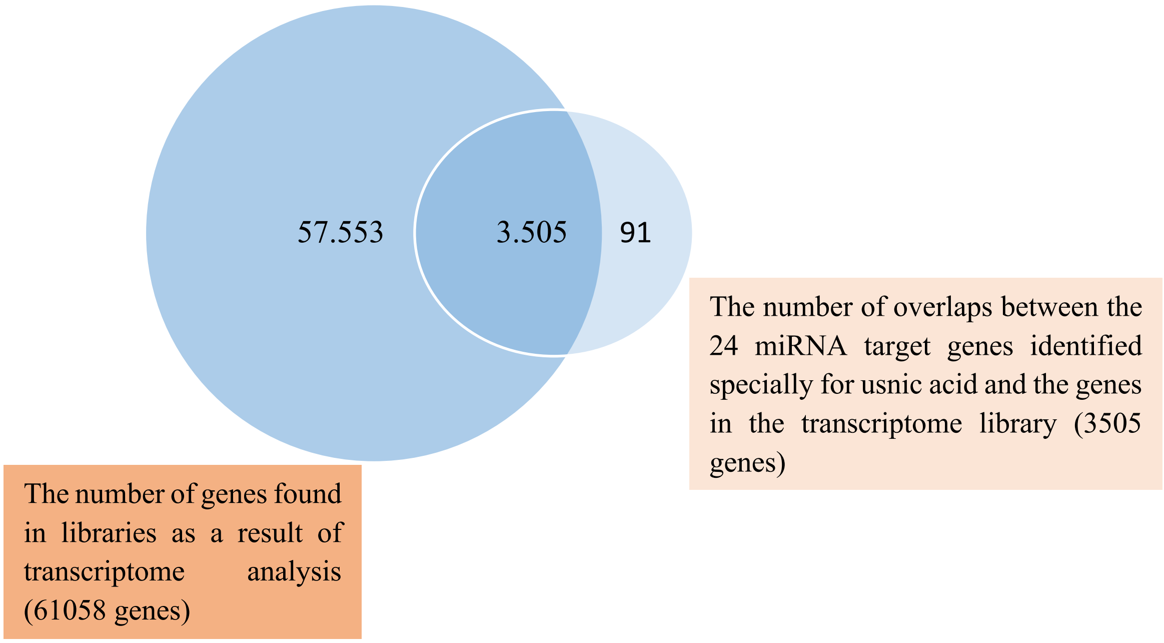
57.553
3.505
91
The number of overlaps between the 24 miRNA target genes identified specially for usnic acid and the genes in the transcriptome library (3505 genes)
The number of genes found in libraries as a result of transcriptome analysis (61058 genes)

Figure S5.

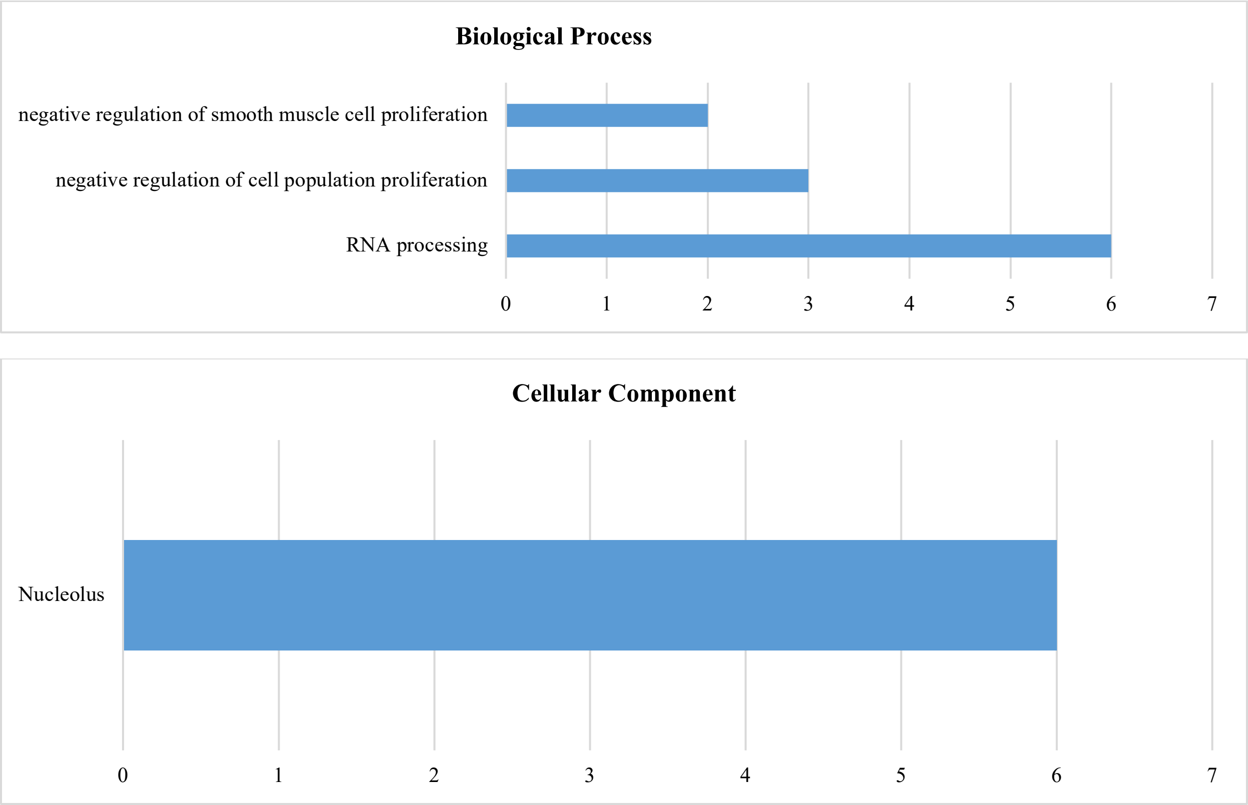

Biological Process
negative regulation of smooth muscle cell proliferation
negative regulation of cell population proliferation
RNA processing
0
1
2
3
4
5
6
7
Cellular Component
Nucleolus
0
1
2
3
4
5
6
7

Figure S6.

A

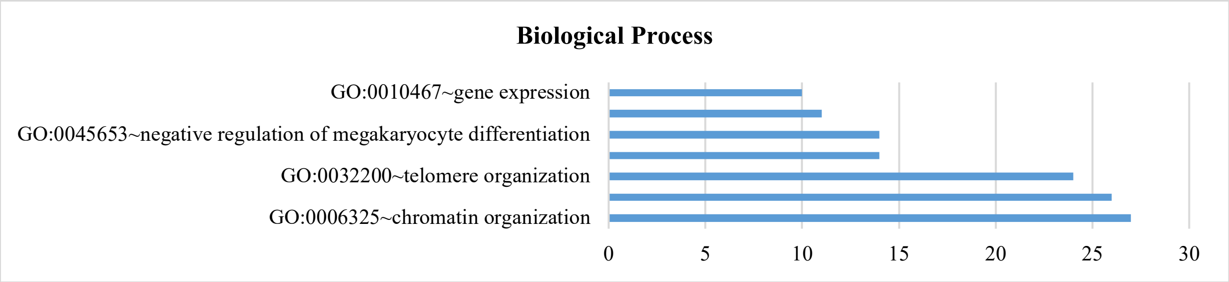

Biological Process
GO:0010467~gene expression
GO:0045653~negative regulation of megakaryocyte differentiation
GO:0032200~telomere organization
GO:0006325~chromatin organization
0
5
10
15
20
25
30

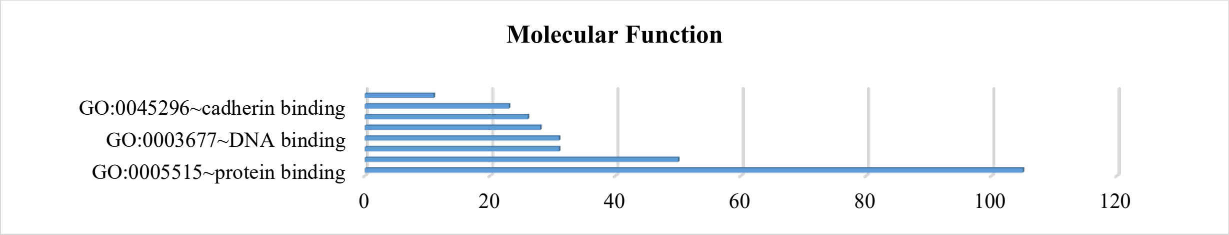

Molecular Function
GO:0045296~cadherin binding
GO:0003677~DNA binding
GO:0005515~protein binding
0
20
40
60
80
100
120

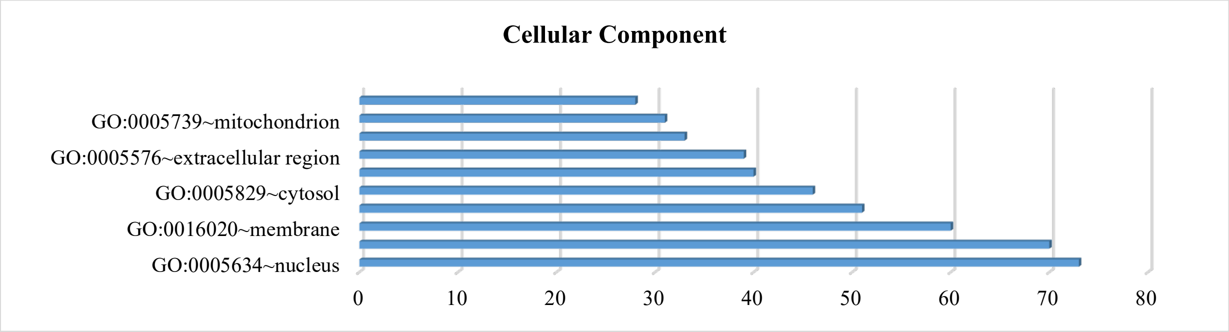

Cellular Component
GO:0005739~mitochondrion
GO:0005576~extracellular region
GO:0005829~cytosol
GO:0016020~membrane
GO:0005634~nucleus
0
10
20
30
40
50
60
70
80

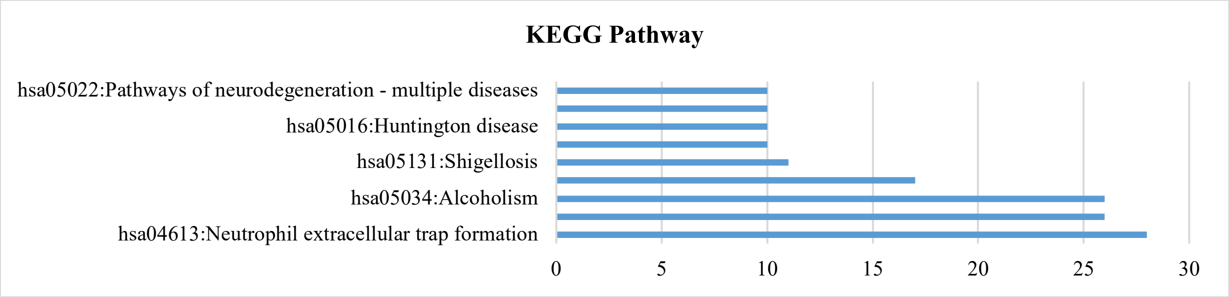

KEGG Pathway
hsa05022:Pathways of neurodegeneration - multiple diseases
hsa05016:Huntington disease
hsa05131:Shigellosis
hsa05034:Alcoholism
hsa04613:Neutrophil extracellular trap formation
0
5
10
15
20
25
30

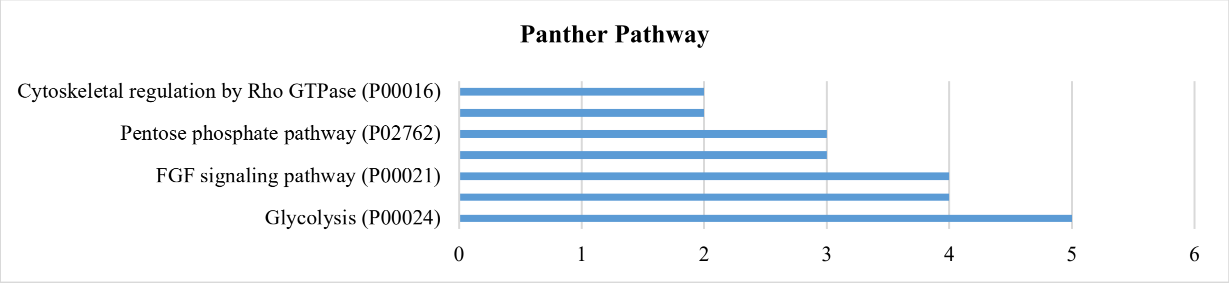

Panther Pathway
Cytoskeletal regulation by Rho GTPase (P00016)
Pentose phosphate pathway (P02762)
FGF signaling pathway (P00021)
Glycolysis (P00024)
0
1
2
3
4
5
6

Figure S6

B

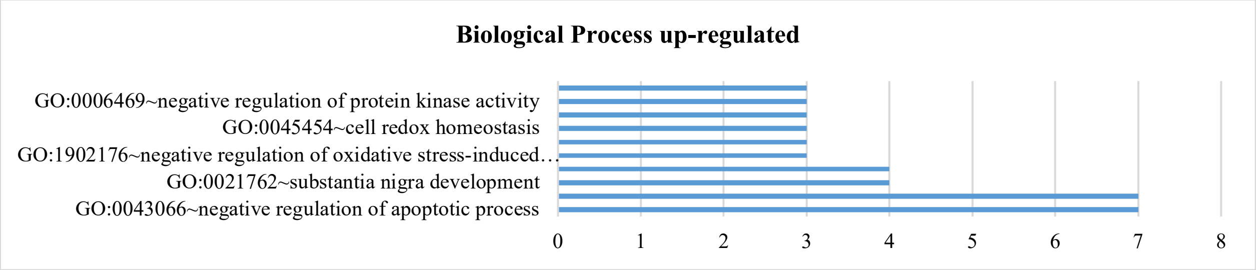
Biological Process up-regulated
GO:0006469~negative regulation of protein kinase activity
GO:0045454~cell redox homeostasis
GO:1902176~negative regulation of oxidative stress-induced…
GO:0021762~substantia nigra development
GO:0043066~negative regulation of apoptotic process
0 1 2 3 4 5 6 7 8

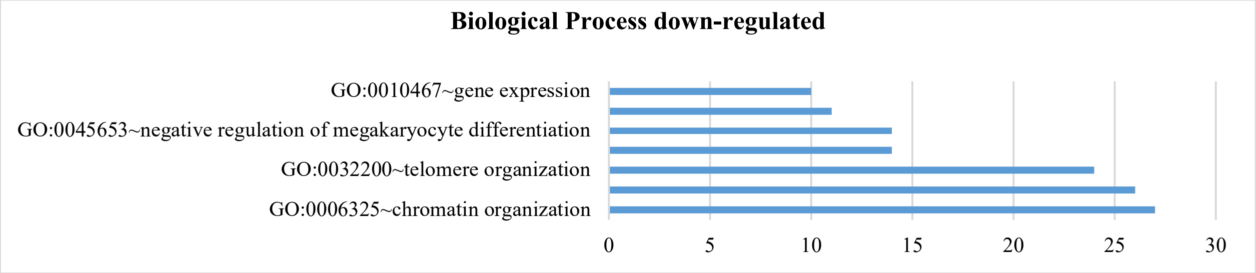
Biological Process down-regulated
GO:0010467~gene expression
GO:0045653~negative regulation of megakaryocyte differentiation
GO:0032200~telomere organization
GO:0006325~chromatin organization
0 5 10 15 20 25 30

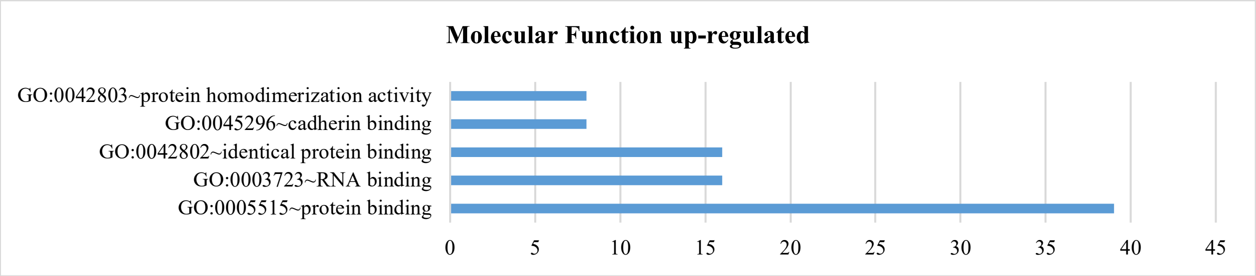
Molecular Function up-regulated
GO:0042803~protein homodimerization activity
GO:0045296~cadherin binding
GO:0042802~identical protein binding
GO:0003723~RNA binding
GO:0005515~protein binding
0 5 10 15 20 25 30 35 40 45

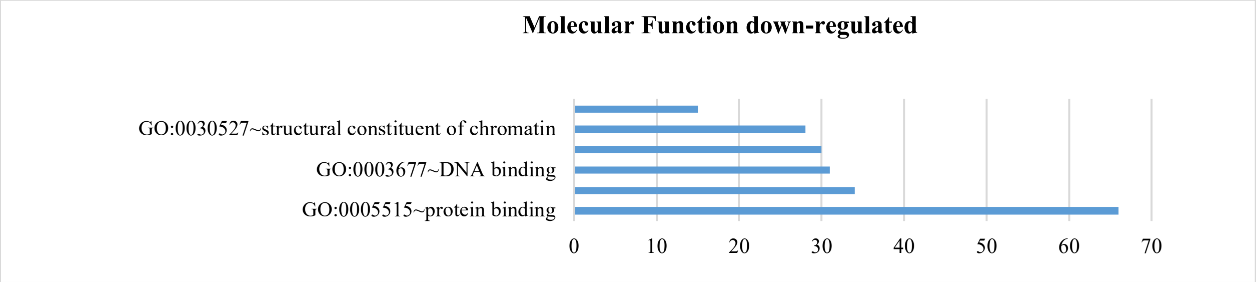
Molecular Function down-regulated
GO:0030527~structural constituent of chromatin
GO:0003677~DNA binding
GO:0005515~protein binding
0 10 20 30 40 50 60 70

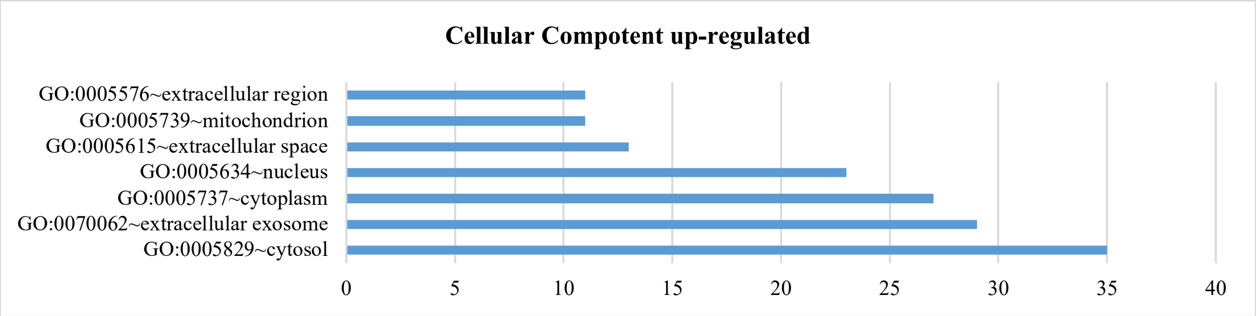
Cellular Compotent up-regulated
GO:0005576~extracellular region
GO:0005739~mitochondrion
GO:0005615~extracellular space
GO:0005634~nucleus
GO:0005737~cytoplasm
GO:0070062~extracellular exosome
GO:0005829~cytosol
0 5 10 15 20 25 30 35 40

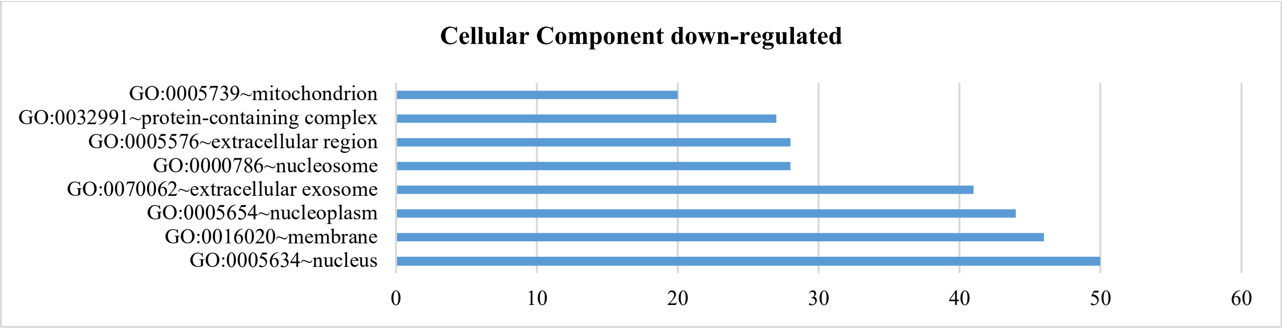
Cellular Component down-regulated
GO:0005739~mitochondrion
GO:0032991~protein-containing complex
GO:0005576~extracellular region
GO:0000786~nucleosome
GO:0070062~extracellular exosome
GO:0005654~nucleoplasm
GO:0016020~membrane
GO:0005634~nucleus
0 10 20 30 40 50 60

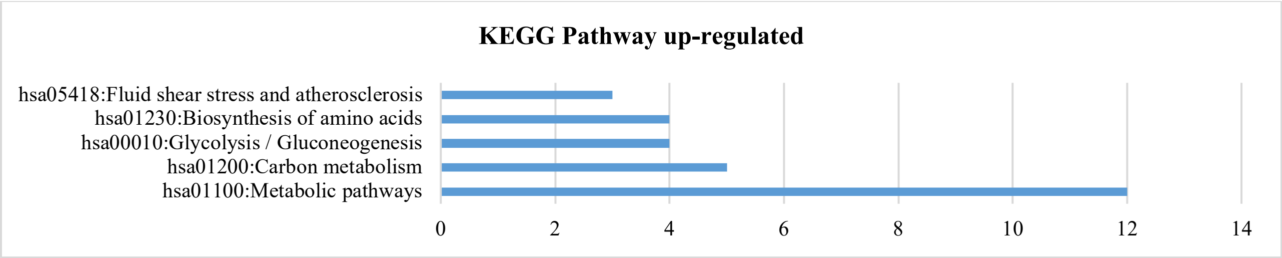
KEGG Pathway up-regulated
hsa05418:Fluid shear stress and atherosclerosis
hsa01230:Biosynthesis of amino acids
hsa00010:Glycolysis / Gluconeogenesis
hsa01200:Carbon metabolism
hsa01100:Metabolic pathways
0 2 4 6 8 10 12 14

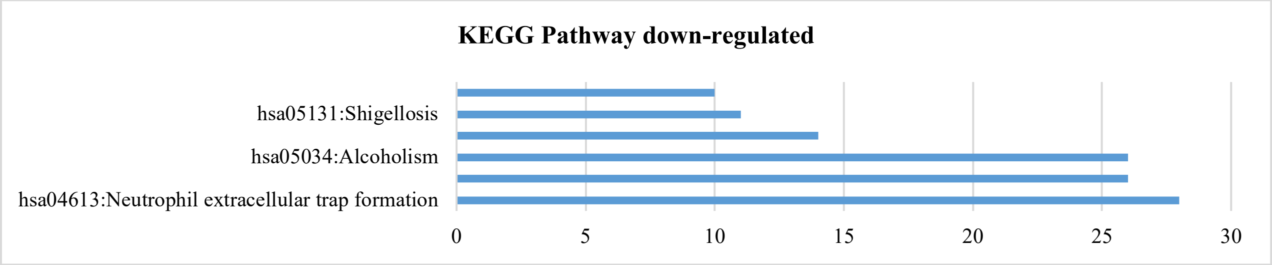
KEGG Pathway down-regulated
hsa05131:Shigellosis
hsa05034:Alcoholism
hsa04613:Neutrophil extracellular trap formation
0 5 10 15 20 25 30

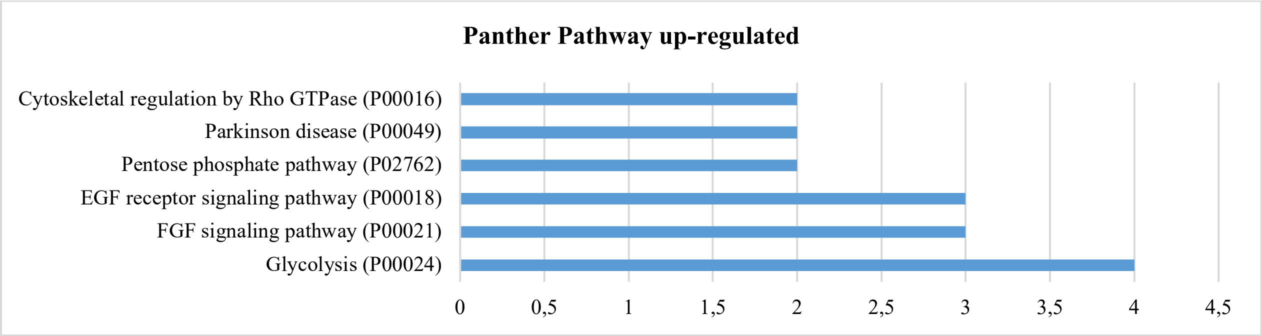
Panther Pathway up-regulated
Cytoskeletal regulation by Rho GTPase (P00016)
Parkinson disease (P00049)
Pentose phosphate pathway (P02762)
EGF receptor signaling pathway (P00018)
FGF signaling pathway (P00021)
Glycolysis (P00024)
0 0,5 1 1,5 2 2,5 3 3,5 4 4,5

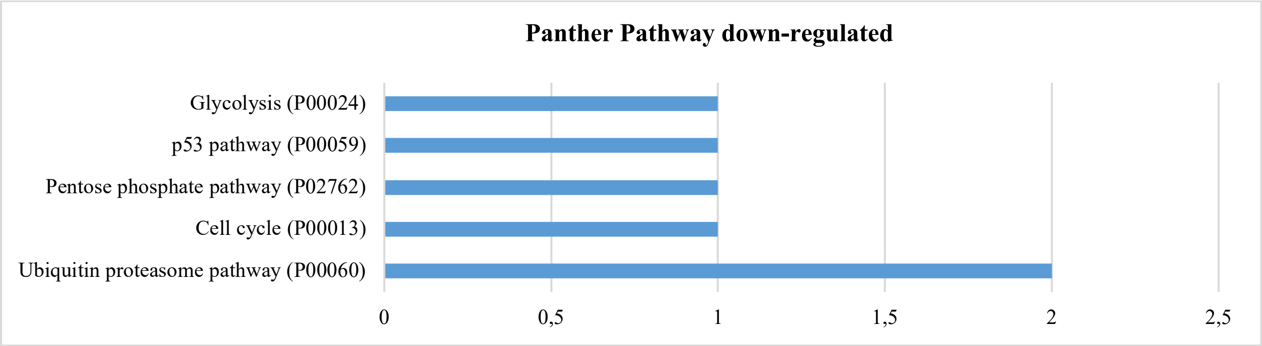
Panther Pathway down-regulated
Glycolysis (P00024)
p53 pathway (P00059)
Pentose phosphate pathway (P02762)
Cell cycle (P00013)
Ubiquitin proteasome pathway (P00060)
0 0,5 1 1,5 2 2,5

Figure S7.

A

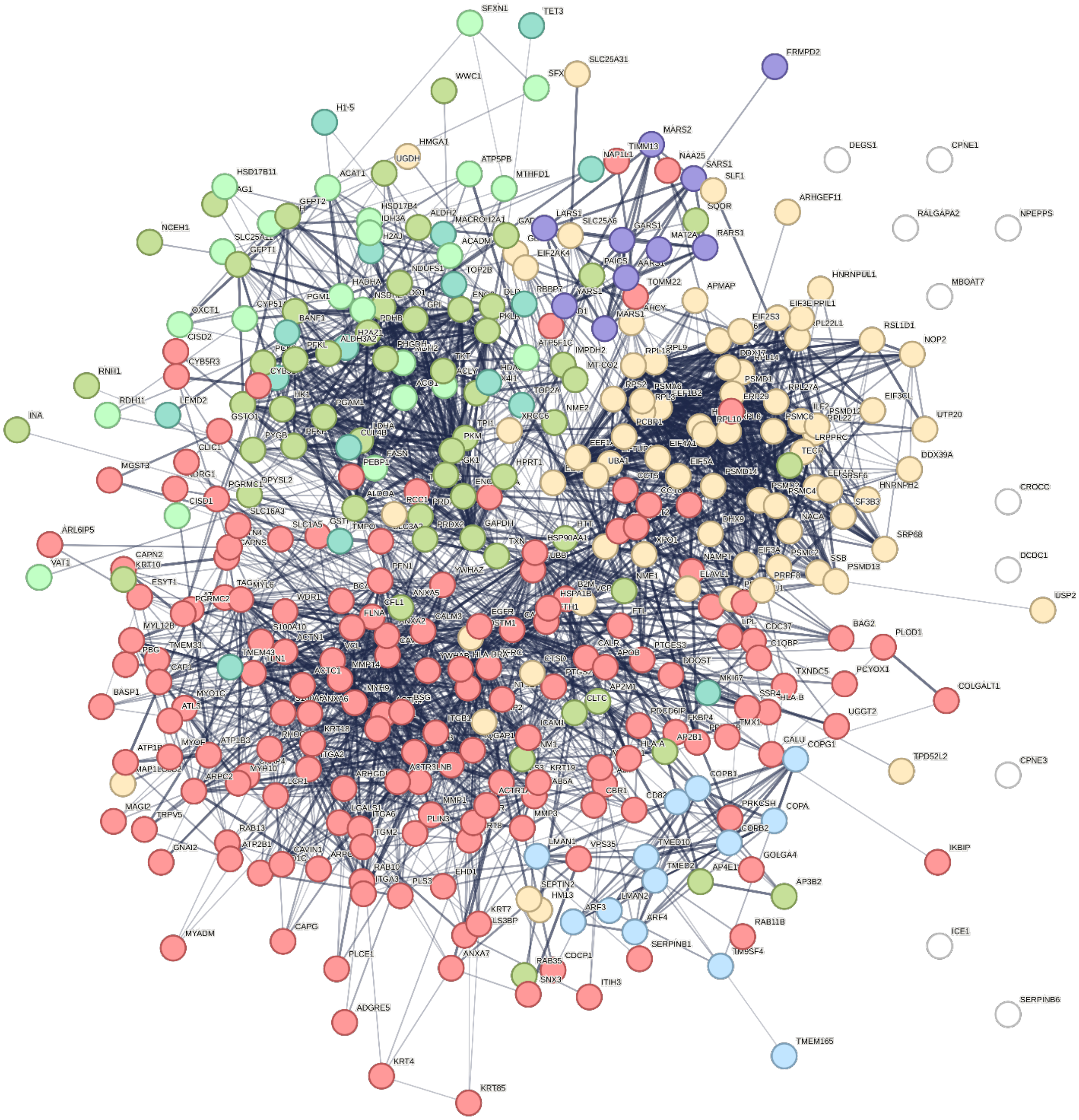
SFXN1
TET3
SLC25A31
FRMPD2
WWC1
H1-5
HMGA1
UGDH
ATP5PB
MTHFD1
MARS2
TIMM13
NAP1L1
NAA25
SARS1
SLF1
DEGS1
CPNE1
HSD17B11
ACAT1
GFPT2
HSD17B4
ALDH2
MACROH2A1
SQOR
ARHGEF11
RALGAPA2
NPEPPS
NCEH1
SLC25A1
GFPT1
LARS1
SLC25A6
GARS1
MAT2A
RARS1
EIF2AK4
PAICS
HNRNPUL1
MBOAT7
TOMM22
APMAP
OXCT1
CYP51
PGM1
HADHA
RBBP7
YARS1
EIF3E
PPIL1
RPL22L1
RSL1D1
NOP2
CISD2
CYB5R3
RNH1
RDH11
LEMD2
INA
GSTO1
CLIC1
MGST3
DRG1
CISD1
PGRMC1
DPYSL2
SLC16A3
SLC1A5
ARL6IP5
VAT1
CAPN2
KRT10
ESYT1
PGRMC2
MYL12B
TMEM33
TMEM43
BASP1
MYO1C
ATL3
ATP1B3
MAGI2
TRPV5
GNAI2
RAB13
ATP2B1
CAVIN1
MYADM
CAPG
PLCE1
ADGRE5
KRT4
KRT85
ANXA7
KRT7
LS3BP
RAB35
SNX3
CDCP1
ITIH3
SERPINB1
TM9SF4
RAB11B
TMEM165
ICE1
SERPINB6
CPNE3
IKBIP
AP3B2
GOLGA4
COPA
COPB2
PRKCSH
COPG1
CALU
UGGT2
TPD52L2
COLGALT1
PCYOX1
PLOD1
BAG2
USP2
DCDC1
CROCC
PSMD13
SRP68
DDX39A
HNRNPH2
SF3B3
UTP20
EIF3CL
EEF1B2
GAPDH
HSP90AA1
PKM
EGFR
ANXA5
CALM3
VCL
ACTN1
MMP14
MYH9
ITGB1
ICAM1
KRT19
CBR1
CD82
LMAN1
LMAN2
VPS35
TMED10
TMED2
AP4E1
ARF3
ARF4
SEPTIN2
HM13
MMP3
PLIN3
EHD1
PLS3
ITGA3
ITGA6
TGM2
LGALS1
RAB10
MYH10
ARPC2
ACTR3
ACTC1
ANXA6
KRT18
ITGA2
ANXA2
FLNA
CFL1
S100A10
MYL6
WDR1
PRDX2
ALDOA
FASN
PEBP1
CCT5
UBA1
EIF4A1
PSMD14
PSMC6
PSMC4
PSMC2
NACA
SSB
PRPF8
ELAVL1
NAMPT
XPO1
HLA-A
HLA-B
TMX1
FKBP4
SSR4
MKI67
TXNDC5
C1QBP
CDC37
DDOST
PTGES3
APOB
CALR
AP2M1
CLTC
HSPA1B
B2M
FTH1
FTL
NME1
NME2
TOP2A
TOP2B
HPRT1
XRCC6
IMPDH2
RPL18
PCBP1
TECR
LRPPRC
SRSF6
EIF5A
EIF2S3
DDX17
PSMD1
RPL27A
ERP29
RPL10
RPL22
PSMD12

Figure S7.

B

Cluster color	Gene count	Primary description
**Red**	164	Focal adhesion
**Brown**	75	Regulation of expression of SLITs and ROBOs
**Olive**	58	Glycolysis / Gluconeogenesis
**Green**	23	Oxidoreductase activity, acting on the CH-OH group of donors, NAD or NADP as acceptor
**Blue**	17	-
**Light Sky Blue**	13	ER to Golgi Anterograde Transport
**Medium Blue**	10	tRNA aminoacylation for protein translation

Figure S8.

A

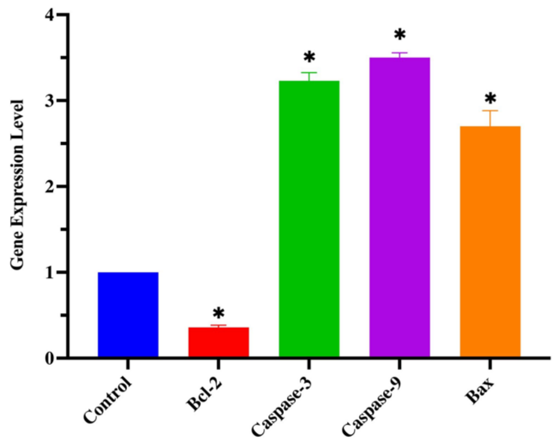

Gene Expression Level
0
1
2
3
4
*
*
*
*
Control
Bcl-2
Caspase-3
Caspase-9
Bax

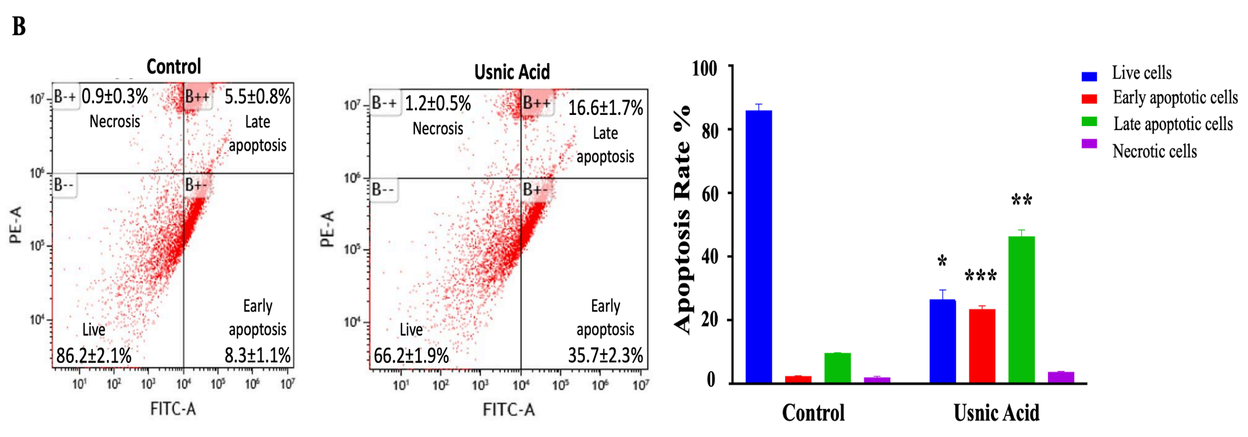

B
Control
B-+ 0.9±0.3%
Necrosis
B++ 5.5±0.8%
Late apoptosis
B--
B+-
Live 86.2±2.1%
Early apoptosis 8.3±1.1%
PE-A
FITC-A
Usnic Acid
B-+ 1.2±0.5%
Necrosis
B++ 16.6±1.7%
Late apoptosis
B--
B+-
Live 66.2±1.9%
Early apoptosis 35.7±2.3%
PE-A
FITC-A
Apoptosis Rate %
0
20
40
60
80
100
Live cells
Early apoptotic cells
Late apoptotic cells
Necrotic cells
*
***
**
Control
Usnic Acid

Figure S9.

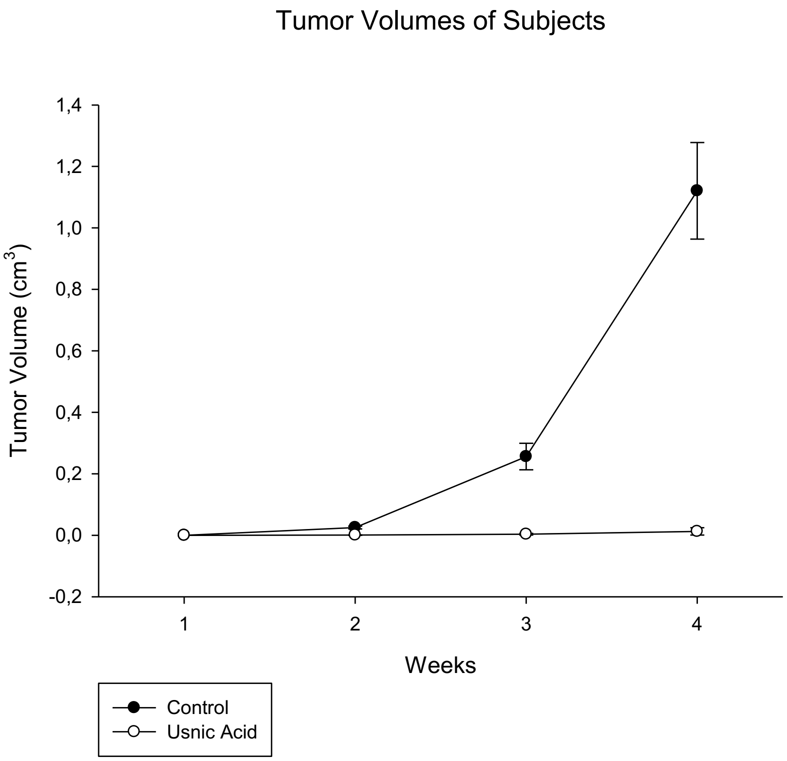
Tumor Volumes of Subjects
1,4
1,2
1,0
0,8
0,6
0,4
0,2
0,0
-0,2
Tumor Volume (cm3)
1
2
3
4
Weeks
Control
Usnic Acid

Figure S10.

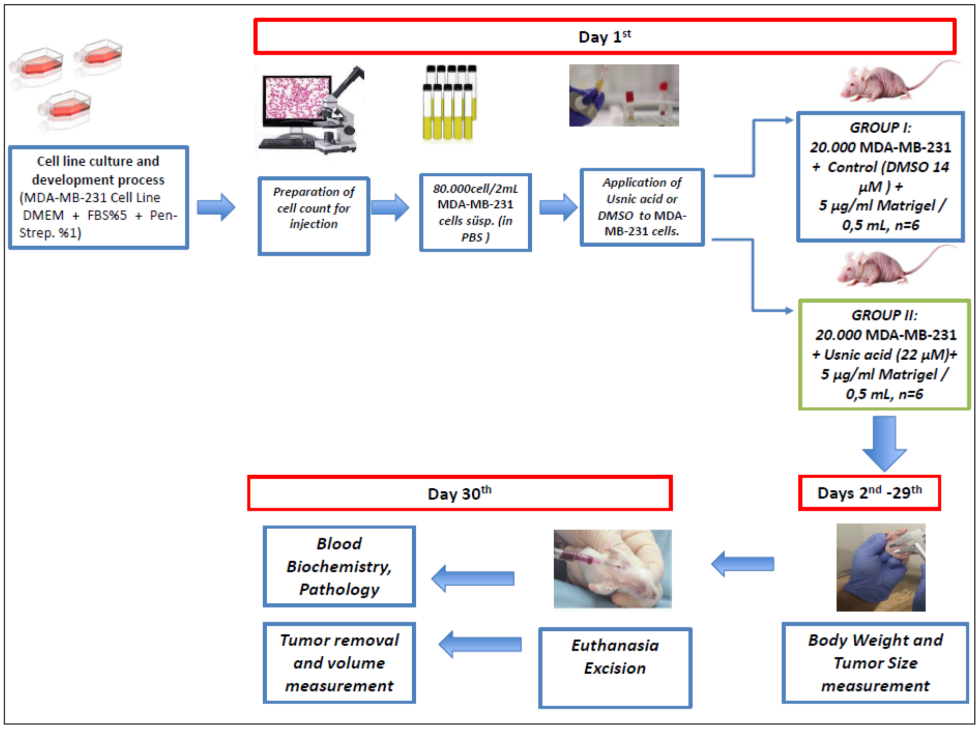
Day 1st
Cell line culture and development process (MDA-MB-231 Cell Line DMEM + FBS%5 + Pen-Strep. %1)
Preparation of cell count for injection
80.000cell/2mL MDA-MB-231 cells süsp. (in PBS)
Application of Usnic acid or DMSO to MDA-MB-231 cells.
GROUP I: 20.000 MDA-MB-231 + Control (DMSO 14 µM) + 5 µg/ml Matrigel / 0,5 mL, n=6
GROUP II: 20.000 MDA-MB-231 + Usnic acid (22 µM)+ 5 µg/ml Matrigel / 0,5 mL, n=6
Days 2nd -29th
Body Weight and Tumor Size measurement
Day 30th
Euthanasia Excision
Blood Biochemistry, Pathology
Tumor removal and volume measurement

Supplementary Tables

Table S1.

Sample	Base Count	Transcript Count	GC (%)	Q20 ((%)	Q30 (%)	Coverage of Expressed Transcriptome	Mapping Percentage
MCF-12A	6,084,810,458	40,296,758	50.11	97.6	93.3	100X	99.92
MCF-12A_UA	5,695,738,724	37,720,124	50.22	97.5	93.1	95X	99.9
MDA-MB-231	5,333,621,094	35,321,994	50.00	97.4	93.0	89X	88.17
MDA-MB-231 UA	5,660,695,852	37,488,052	49.24	97.5	93.1	94X	88.33

Table S2.

					Normalized abundances					
ID	Peptide count	Unique peptides	q Value	Mass	MCF_12A	MCF_12A	MCF_12A	MCF_12A_ UA	MCF_12A_ UA	MCF_12A_ UA
Q01082	23	21	0,02	275408,117	11797,59897	10044,94429	34611,19999	2134,289292	1420,938145	4000,33289
P02794	5	5	0,01	21396,7599	1334,890433	774,6111252	721,0777703	232,3919582	143,7394462	83,97680624
P29401	33	29	0,02	68562,0705	26292,19911	24236,49275	21632,07134	5749,195897	5739,827669	1421,562612
B5ME19; Q99613	9	8	0,01	106157,455	3535,902914	3546,144909	10049,07994	1338,685359	1031,202393	771,1767441
Q15046	4	3	0,04	68504,4373	7087,938749	5255,555717	25695,44316	2901,322869	2141,031137	2023,229051
Q14914	5	5	0,02	36098,0298	2669,513292	6203,79032	2275,448586	917,5354221	642,3571162	1024,59426
Q9Y3F4	4	3	0,04	38780,5362	1150,167009	1169,270825	2339,789963	554,9932025	420,2350878	119,7931454
P08238; Q58FF7; Q14568	39	13	0,00	83606,5094	16950,14904	14246,31552	11937,4922	3471,148223	3871,663558	3101,343461
Q15691	4	4	0,03	30170,211	2804,109405	2240,636335	1140,102229	607,1201868	685,5705616	227,3229767
P06744	16	15	0,05	63375,3545	6097,932826	5285,762727	3034,663341	1771,246828	1571,915462	373,5474753
P30086	5	4	0,02	21170,8601	5786,444373	2230,628218	4975,430844	1313,80089	1265,895752	805,9484175
P62258	17	9	0,00	29345,0493	12295,65539	13039,43402	11655,96317	4313,589867	3452,071254	2868,52446
Q9NYU2	14	13	0,01	177931,219	4434,086444	6194,049166	9026,490815	2349,377327	2086,084424	1502,924336
P04792	15	14	0,01	22839,5719	42771,19031	33862,94768	36524,25796	14148,69029	13844,95788	7545,465059
P62937; Q9Y536; A0A075 B759;A0 A075B76 7;A0A0B 4J2A2;F5 H284;P0 DN26;P0 DN37	17	17	0,00	18240,6427	28985,75914	24968,71536	35587,78411	11196,7881	8942,295988	8166,620052
P60174	21	19	0,00	26954,6554	20441,80535	17809,60609	24931,85203	6521,237178	6213,246361	8308,005554
Q5TCY1	3	3	0,00	143079,49	147,6241183	154,4832853	129,5650952	52,70229111	44,81767756	48,29078928
P07108	4	4	0,01	10044,4483	2387,853022	3507,200726	1779,893968	1071,030738	756,0354017	767,7701915

P52565	5	5	0,03	23264,1679	5004,238607	3981,80387	6603,020696	2328,249604	2107,502528	892,4556624
Q01469; A8MUU 1	9	8	0,02	15506,6309	3001,420643	2170,198859	3410,949423	1353,545001	1065,339641	595,6926035
P12277	6	6	0,02	42929,5023	2051,095048	1427,827014	1036,924775	569,8567031	653,5775871	384,2634404
Q9UKK9	5	4	0,02	24612,7881	944,3410762	792,1851266	975,7853762	448,163096	351,1442045	173,4147275
Q9UMX 0;Q9NR R5;Q9U HD9	4	4	0,04	62519,1205	1440,006324	1015,363573	2657,195149	469,9593432	588,4828882	778,9254753
P09211	13	12	0,00	23583,9696	20187,36341	12807,64806	15958,54142	6846,515146	6004,052442	4963,409235
P09382	9	9	0,01	15057,8861	22697,45544	16315,15164	19594,69824	6020,88102	5683,955959	10629,55832
Q5T9A4	10	4	0,05	73143,3439	15637,10222	14083,67754	23501,5598	8795,645953	8968,402299	3630,35463
P06733	43	32	0,04	47511,201	13302,3092	28865,48691	15910,53541	9071,388262	9558,132304	5513,061358
Q16643	4	4	0,04	71885,587	1215,599934	886,4944342	2031,585591	661,7283006	643,9023351	428,1228562
Q16881	11	10	0,04	71725,5978	4115,606567	2605,063447	3032,892712	2210,407633	1035,167065	850,7928117
P46778	7	5	0,05	18621,9087	7041,246216	8120,481709	3528,575837	2633,977824	2142,629435	3197,631358
P14618	38	34	0,02	58507,2826	20528,06384	27270,78232	19621,66323	12600,94478	10936,65458	6116,856592
P07437	31	4	0,04	50127,1555	24019,36538	14167,98398	30968,69717	11747,97802	10244,87838	8591,727608
P23526	12	7	0,00	48286,4702	2639,374108	3410,93959	3950,590067	1639,895191	1426,507681	1459,886538
O60936	3	3	0,04	22857,5821	1596,120396	1079,238532	1021,017563	712,9544659	637,949195	335,7067491
P43490	6	6	0,00	55806,3386	2066,606199	1515,435437	1819,001331	756,9530326	917,9334061	791,8028096
P15559	7	5	0,00	30924,7316	2581,401642	2452,003886	2458,233363	1149,323863	1089,961538	1238,929431
Q9BT78	7	6	0,05	46554,0352	902,9328382	707,7549335	803,0526327	554,1853515	366,64083	201,5602472
Q13442	5	4	0,02	20630,0464	1772,600905	1405,700612	2254,102266	973,2137674	943,8702666	611,5307443
P30041	16	16	0,00	25149,0747	5987,656507	7728,005253	7364,658629	2850,48091	2967,756186	4030,486962
P27348	14	9	0,01	28049,4331	3004,900667	3831,494706	2776,378634	1694,380049	1666,776022	1228,448295
O00151	11	11	0,02	36527,9836	5889,649083	9321,438965	6838,89606	3336,686288	2950,566616	4424,685427
P23528	14	6	0,02	18730,6344	18375,66622	13543,3826	19599,65148	6878,277556	6929,072138	11442,41201
Q00325	19	19	0,01	40551,166	11085,93862	12123,21721	8140,562228	20342,22999	19233,25487	23626,98141
Q969X5	3	3	0,03	32991,5365	543,8936059	756,7620829	545,0879882	1353,249691	1580,391003	860,2974999
P0C0S5; Q71UI9	5	4	0,02	13552,7525	3510,253322	3610,717265	4312,888936	5992,116215	6691,451163	10877,04009
P27824	18	16	0,02	68024,6172	17062,17359	13467,99752	19172,97833	29120,51944	27728,34359	47151,09796
Q15233	13	12	0,00	54345,7596	4390,177457	4407,405008	3217,8848	7958,67255	7830,875707	9367,864527
P35232	10	9	0,01	29861,1489	6440,456358	6998,528094	4417,780595	12576,86894	13885,46094	10928,27925
P09669	4	4	0,04	8781,4693	2404,794771	1088,541036	1570,213552	2916,205561	3593,567598	4116,60956
P82933	5	5	0,01	46063,0062	1778,289543	1250,408217	1942,896231	3137,722856	2963,925815	4351,631987
P42126	7	6	0,01	33101,1723	5651,785093	6878,577867	4125,828008	11582,11376	11532,92353	12092,04151
Q6WCQ 1	4	3	0,02	117331,714	4060,703378	3376,442878	5356,277765	7110,697352	9043,285407	11112,50986
Q9Y6C9	3	3	0,01	33958,2571	1155,741339	681,8518966	628,6879021	1839,73901	1741,055322	1854,412265
Q04837	7	7	0,04	17259,6938	4135,903562	4514,318601	4456,740112	7114,114605	7090,694993	14891,62902
P84103	6	5	0,05	19557,7197	7936,181609	4975,378252	3331,930344	13477,02893	14259,24541	8446,649779
P52272	30	30	0,04	77801,0794	4023,635699	3394,670306	5229,610301	7023,233082	7459,402924	14564,37901
Q10471	3	3	0,00	65474,241	1069,625961	1275,07634	1017,073917	2139,73403	3214,926861	2451,772101
O76003	3	3	0,01	37717,2319	1753,676013	1818,641542	2677,126882	4540,305788	4227,208815	5798,069324

Q12873	4	3	0,03	228131,805	151,219813	252,9244321	344,2774541	458,3803519	539,1921924	760,7280118
P07910; B2RXH8 ;B7ZW3 8;O6081 2;P0DM R1	11	10	0,03	33727,0878	9468,844268	9403,209953	4880,361284	15616,85374	15896,94357	25441,53487
O95292	4	3	0,01	27456,5596	2014,252586	1323,065144	1625,431607	2920,300133	3758,82817	5288,281113
P33121	19	15	0,03	78969,9856	1444,393403	2135,380878	2824,627026	3814,795389	4373,880291	7320,249295
P38159; Q96E39; O75526	11	9	0,01	42331,9107	3349,84594	4143,903581	5856,64196	9370,797513	9991,965155	13985,36717
Q15424	8	7	0,01	103097,959	617,7255547	1224,749839	618,0337794	1936,283925	1934,35899	2277,801006
P56385	3	3	0,04	7933,2197	408,5815847	1030,851949	364,800054	1794,548671	1574,753991	1238,923861
P24539	11	11	0,04	28965,7127	2124,72543	3437,227563	1988,491805	5667,130734	4211,826494	10020,78658
Q13423	35	34	0,01	114637,21	4200,05634	3743,042339	4897,127957	10532,3607	15378,19567	8515,275995
P62805	15	14	0,01	11367,3627	91006,34923	88138,27503	65279,71507	163383,8903	195644,4761	320274,7244
Q12907	6	5	0,00	40570,8994	1152,513595	1608,585697	1320,352722	3737,128874	3560,410759	4100,779445
Q16654	6	5	0,04	46811,4921	690,2362319	642,1675015	517,8212669	1167,338683	1171,563668	2962,012688
P05141	12	4	0,00	33080,4023	3118,938793	3240,089264	4322,068603	9612,126479	8864,209805	12331,83917
P02545	62	53	0,03	74424,718	19815,3798	26939,57858	12541,88583	39843,15742	46348,39976	85268,59823
P16401	8	8	0,01	22580,1909	7151,312083	5670,904561	4509,259261	13561,56568	15369,92329	22255,70736
P14927	6	6	0,05	13530,4773	3634,33768	2936,466334	4033,722809	7830,930787	5855,123712	17706,69852
P08195	11	11	0,03	68222,2132	1240,339122	1564,419115	2309,900105	4187,400863	3938,477132	10023,91536
O00231	8	7	0,03	47748,9824	2454,194144	8093,839674	3038,731016	17715,43937	19678,93675	10842,14687
P12236;P 12235	10	3	0,01	33094,4353	1299,532186	1227,050659	1713,244794	3954,681263	3362,697092	7928,69745
O14776	6	5	0,04	124129,413	676,6798282	848,5044997	390,8567647	3133,63002	3078,190843	1054,868727
Q12906	25	21	0,00	95737,7497	3901,697049	4393,220481	5675,604953	21040,08354	20814,54272	12009,8234
P18754	11	11	0,00	45425,3299	990,3873917	1594,817889	1065,586742	3666,303929	4130,849576	6921,886997
P04179	15	14	0,01	24921,2554	5081,11247	9649,232388	6778,972118	29579,59372	38230,76093	20620,61819
P25705	29	28	0,01	59864,7823	6784,572186	12381,90945	7683,452602	28602,84632	28292,01788	54186,87959
Q92522	3	3	0,04	22487,14	564,1181304	1708,032759	623,4480316	2576,511054	2444,412369	7051,682176
Q8TCS8	14	13	0,03	86578,064	2135,61236	2107,060832	2059,561236	5326,260985	5066,037243	16532,86346
O94826	8	7	0,02	68139,2703	638,3649001	1791,813141	939,5278499	3439,823401	3517,091483	7973,975952
P62195	10	7	0,04	45797,2139	4062,846187	4637,999384	4052,339779	30073,33911	20308,69792	6942,221072
Q2TB90	6	3	0,03	103856,665	1875,400762	2131,242017	1981,162239	14202,21272	14812,21544	3551,293986
P62316	8	5	0,05	13640,9634	625,0286474	652,5677851	555,6887123	1762,245799	1472,708722	8035,567344
Q8WXH 0	20	9	0,02	802317,599	450,8735585	871,5962901	1206,377426	7206,593761	2126,541292	7079,137249
P31947	8	4	0,00	27888,1757	1236,111675	1330,785202	830,2365181	5613,308202	6975,463196	11612,09933
P68431;P 84243;Q 16695;Q 71DI3;Q 6NXT2; Q5TEC6	7	5	0,01	15518,1658	8625,889264	6166,469414	2890,482845	32464,3208	43057,26035	108958,1894
P04844	18	16	0,00	69398,1357	1563,034526	2935,424055	1855,438864	17781,32256	43170,45106	14005,26768

Q9BTM1;P0C0S8;P20671;Q16777;Q6FI13;Q96KK5;Q99878;P04908;Q7L7L0;Q93077	11	8	0,04	14019,4234	46351,60964	366,0732905	3336,072267	172824,5163	221178,9841	448172,3348

Table S3.

					Normalized abundances					
**ID**	**Peptide count**	**Unique peptides**	**q Value**	**Mass**	**MDA-MB-231**	**MDA-MB-231**	**MDA-MB-231**	**MDA-MB-231_UA**	**MDA-MB-231_UA**	**MDA-MB-231_UA**
Q92769	5	3	0,00	55934,59	1050,183799	1568,653603	3069,247326	88,0492758	152,4205069	56,38540138
Q14CX7	4	4	0,00	113205	986,6091824	1327,635865	1287,928725	25,28946117	47,16616698	138,0004509
Q8NBU5	5	4	0,00	41086,29	16305,44505	23418,04261	24982,92475	1486,863245	1908,93168	2051,893569
Q9NQA5	6	4	0,01	83349,24	535,0863128	1722,584606	422,132095	105,4974265	104,178177	80,05102863
O75691	4	3	0,00	321009,2	18576,00305	12999,82769	10294,22159	1624,795933	1712,07388	1693,328857
Q06033	7	7	0,00	100134,5	117627,0629	111861,394	64355,24633	14533,02026	11018,937	12390,62329
Q9BQI6	4	3	0,01	122533	1859,113727	3030,951991	4802,042844	328,2656955	198,4659686	738,5174253
Q16576	8	3	0,01	48162,31	8790,501034	8680,578722	2027,894497	784,0714649	915,270116	922,957271
Q02880	6	5	0,00	184236,9	44419,99472	44360,41299	25369,35336	6116,318239	4866,951265	4687,901412
Q2PPJ7	5	5	0,00	213222,4	2262,208389	3250,443106	3921,844907	415,5119171	420,2137075	474,6069559
P06858	3	3	0,00	53732,84	5523,234926	5337,340148	5766,803869	891,3355679	746,1599609	846,0087589
Q86UL8	4	4	0,00	159553	1208,946867	1535,399621	1152,328178	143,3056122	198,5249934	245,937191
Q16850	5	5	0,00	57205,09	30304,62643	24205,52992	22582,84862	5011,040602	4785,071016	5576,330505
Q99259	3	3	0,00	67638,07	2138,771997	1731,235779	1846,422932	358,3875637	381,8586316	600,2780884
P11586	12	11	0,01	102243,7	107709,9131	64389,93564	33496,90745	22099,13538	12393,14754	14140,41002
Q8IX03	3	3	0,01	126099,4	1315,648115	3062,128464	2453,324527	597,572448	636,7431948	444,1489719
O14980	9	9	0,00	124526,7	31627,48406	27539,82634	20230,79231	8525,023558	6007,591221	6114,942071
Q9P2K8	4	3	0,00	188393,5	17520,6627	22583,03637	14985,73054	5661,055089	4665,046909	4002,48574
Q9UPU5	3	3	0,01	297388,2	925,4767275	1669,691925	1627,308702	190,8702239	475,6424723	509,9746485
P01023	10	10	0,00	164716,9	110599,2382	90031,98116	70937,31758	29443,68912	24735,11253	21488,20736
A8MWD9;P62308	3	3	0,00	8601,11	37798,74358	29946,86846	20561,05208	9786,322411	8007,59595	7363,915077
Q96GW9	3	3	0,02	67503,41	30705,76576	12623,15548	9386,871576	4445,381435	5350,053085	5298,180026
P05091;P00352	15	13	0,00	56894,65	43171,51487	40274,87713	38025,62314	12890,57475	11215,36926	11979,2711
P28331	7	4	0,00	80494,17	1986,082638	1318,465107	1710,588943	316,9379388	569,1254382	605,435147
P55809	7	5	0,00	56613,95	9073,961452	9794,212541	7790,830312	2521,356682	2593,856021	2873,045795
P16401	9	8	0,00	22580,19	74730,5861	76558,54852	51387,23384	21481,19881	22179,7078	17700,41994
Q05193	3	3	0,00	97807,65	4343,934556	2678,518012	3560,323669	1167,000309	1192,97883	890,6583791
M0R2J8	8	8	0,00	202359,6	9622,791731	8296,097271	7001,82079	2476,481466	2989,32681	2226,590759
Q6P5R6	3	3	0,00	14606,52	4506,699862	4388,429696	6033,623227	1336,943145	1618,836304	1762,405518

P55072; Q8IYT4	31	30	0,01	90006,29	274798,9505	330297,4378	187893,2861	108466,6087	66225,13648	81085,03303
P0C0S5; Q71UI9	6	4	0,01	13552,75	23971,36885	36718,64007	35324,35484	14268,29412	8012,940895	8721,24595
O43151	6	6	0,00	195928,8	5097,113765	3915,462362	4019,602509	1349,523062	1438,271318	1426,329135
O15085	7	4	0,00	168560,1	13757,18365	15767,84018	11894,48987	4601,021619	5186,999617	3771,592722
P13929	9	4	0,00	47329,12	10116,08428	7079,700528	10368,34614	3099,161591	3161,121577	2892,614347
P24539	5	5	0,00	28965,71	29319,87131	40761,6043	33038,52922	10296,72818	13794,2595	10263,32635
Q15029	15	14	0,00	110405,4	35526,48472	32685,45876	24015,67538	9085,452152	11785,21922	10977,05006
P04114	6	6	0,00	516974,1	7347,126215	13611,16451	11276,90124	4226,475514	3765,603579	3180,453564
Q9NYU1	5	4	0,02	175362,9	1721,8794	896,0062402	1625,284165	755,2092445	262,1180136	461,8226546
P40926	16	14	0,00	35959,55	165348,2039	157018,8907	145756,5248	72828,3214	41411,36157	59516,3301
P17931	4	4	0,00	26209,39	5781,537533	6703,396959	7847,566557	3185,317562	1933,603073	2471,022039
P46013	6	5	0,01	360918,2	2195,610127	1422,306766	1158,301442	474,6756221	886,2652981	435,3236059
E9PAV3; Q13765; Q9BZK3	8	8	0,01	206106	29110,891	51225,21209	43513,08575	19670,73439	12519,74994	15493,21483
B5ME19; Q99613	9	9	0,00	106157,5	51553,11064	55533,2794	34701,65187	21589,41503	19717,89178	18783,7231
O43175	10	10	0,00	57391,96	12971,98984	16077,3526	17670,04114	8284,182013	5135,030122	6650,946297
Q9NZ45	4	3	0,01	12370,16	26372,50594	16295,71199	12591,79241	8665,251341	7436,604778	8451,992948
Q15717	6	5	0,00	36263,04	6167,73371	4857,278693	5976,853381	2888,232308	2090,35536	2744,736104
P50213	9	8	0,00	40048,02	26565,37406	22379,71852	17520,21464	9009,990942	10781,4196	10524,70147
Q9Y5L4	4	3	0,01	10728,18	15801,94174	13410,54536	11262,17904	8197,262299	4832,654418	5493,767274
P49591	10	10	0,00	59290,62	16305,17209	19014,50372	16887,83946	7525,447529	8527,406147	8768,575749
Q9Y2F5	8	8	0,01	250913,5	7483,995783	5687,306976	5391,7455	2603,36657	3550,646058	3122,650351
Q92544	3	3	0,00	75260,28	2605,595335	2908,646301	2648,783152	5028,970717	5960,074312	5349,382154
Q9BUJ2	4	3	0,01	96308,92	1682,595678	2420,848519	2772,061428	3737,897866	5541,174214	4517,638651
O00264	4	3	0,00	21785,23	3245,633511	3236,6305	3171,182945	6262,012606	7516,271893	5671,657014
P39656	10	8	0,00	50971,97	21837,25679	23042,68264	20241,17654	44040,01407	51450,34221	36006,03491
P11177	6	4	0,01	39575,66	13574,94484	12745,71505	16517,3266	35851,71196	25133,15654	25528,90324
Q16543	7	6	0,00	44981,65	5332,041099	3871,622493	4381,155124	10946,80875	8647,150265	7899,010624
O94808	4	4	0,01	77729,12	2828,774042	2660,281039	3063,92679	7953,504358	5468,802854	4032,120244
Q9P2J5	9	9	0,00	135664	6836,02626	7482,864507	6870,846278	16483,1674	13143,75564	13890,51812
P47756	3	3	0,00	31635,68	2366,749817	2557,024457	2507,932777	5629,4314	4463,090732	5175,295576
P61026	12	7	0,02	22769,08	5809,598001	4755,434006	3750,288277	13985,88041	7920,77686	7547,248193
O43681	3	3	0,00	39249,11	1517,078795	1082,460233	1290,254099	2472,578657	2704,734931	2827,081449
P50990	20	17	0,00	60190,99	33170,0694	34724,48408	31165,48146	76257,59903	64855,92416	63128,4692
P50991	15	14	0,00	58437,6	19469,9737	15855,83183	18493,80406	45475,8447	32826,50469	32982,84652
P23526	13	11	0,00	48286,47	14744,96176	10930,08822	10561,16742	27228,2074	23210,02643	24573,88622
Q15907; P62491	7	5	0,01	24602,58	7519,826404	7272,969094	8585,91016	21043,36741	14694,55581	12932,40059
Q9Y4L1	30	26	0,00	111563,7	40069,73168	33408,40607	36167,98775	85776,2083	72707,50501	70282,53583

O75369	33	23	0,01	280331,4	17189,18742	15069,30977	19217,00309	44937,46581	29338,56916	33280,81382
P09622	10	10	0,00	54747,62	12186,13161	11587,72904	13997,9949	27849,13549	25678,68486	25461,8756
Q9UPM8	3	3	0,01	128485,2	3276,168787	2952,760092	1869,771054	5938,626738	6008,943738	4992,017441
Q12907	9	8	0,01	40570,9	6374,817858	9273,99527	11898,82189	22220,05554	19199,53551	16514,70342
P15880	14	13	0,00	31609,62	48604,90949	52388,79341	50909,91995	137239,7752	95982,55623	91255,82341
P27797	20	19	0,00	48312,69	169560,4211	171889,5474	189759,7726	453130,2346	359503,4542	324073,9459
Q6NZI2	8	6	0,00	43476,19	7176,719226	8391,962312	7656,385466	19328,53669	15886,56414	14512,11718
Q5TZA2	8	7	0,01	228922,7	1171,961582	2412,528278	1856,158177	3869,90371	3705,653066	4087,460831
P63010	5	4	0,02	105465,2	2605,510432	1362,790267	1028,907483	4206,200781	3016,529008	3493,238824
P27635; Q96L21	10	8	0,00	25060,16	7684,451278	7303,299141	10271,23578	17078,80031	21682,68546	15668,68988
Q6P2Q9	23	21	0,00	274912,5	8845,005818	7963,856723	8362,580888	17060,6952	20513,04238	16865,38695
P50914	4	4	0,00	23545,95	9402,195493	9504,399307	9527,603441	21908,70693	22111,75431	17505,69807
O15173	3	3	0,01	23875,52	2632,29527	3093,62097	2315,907852	7951,486187	5584,538289	3865,012576
P51659	8	8	0,00	80142,69	2945,806086	3120,456708	2713,463718	6084,369202	7081,248329	5903,519046
P51153	6	3	0,01	23002,27	1380,564127	932,6163371	724,5848131	1629,476477	2623,930455	2349,689738
Q8NBJ5	7	7	0,01	71978,43	2527,844112	1656,346679	1638,996186	4034,981007	4814,20298	3829,806809
Q15185	5	5	0,00	18982,56	3934,633516	3577,510538	3594,140515	9244,1885	8711,301754	6339,872559
P11388	8	6	0,00	175126,8	16396,24006	17069,32461	15858,44793	41756,28806	35479,18999	31056,02974
Q02809	5	5	0,00	84120,58	2327,755639	2611,151043	3125,754007	6359,093927	5480,798305	5968,040183
P46087	3	3	0,01	89644,08	10202,04812	11068,04761	14761,31345	34273,25364	23284,94235	22041,33869
O00232	7	7	0,01	53303,72	5583,350248	6200,591536	3561,409048	12902,01616	11053,20805	9951,394128
P11216	8	7	0,00	97380,45	1589,252971	1369,391455	1171,080658	2710,286984	3170,872197	3274,750239
P46940	31	28	0,01	189879,6	19160,71914	17670,24812	14860,09682	50032,04272	31497,33126	33721,3909
Q13439	6	5	0,01	262053	8388,201931	7749,567955	9140,552186	24014,82351	13030,91267	19463,41471
P54136	11	10	0,00	76177,51	10186,88991	6973,511487	7092,373026	20161,50427	17597,55395	16701,75022
P78371	15	14	0,01	57830,49	15653,14664	10805,4336	11398,19069	35595,53861	25760,21296	23736,77671
P62333	8	8	0,01	44458,22	5710,751636	5391,246587	8721,755647	17286,93779	14490,29845	13025,11242
P49755	6	5	0,01	25147,1	6906,091872	5125,762349	7475,599443	18528,75152	13726,89641	11891,9576
Q8WUM 4	13	12	0,00	96650,59	6314,367832	5970,974525	5294,208225	15168,92887	11938,94232	12719,99553
Q9NZM1	56	52	0,00	236248,8	59678,94345	56579,97465	56484,90652	140021,8464	140457,3559	112198,9922
P13489	8	8	0,01	51798,39	3458,714841	3224,965419	5139,754122	11329,2675	7929,54087	7656,751755
Q9Y678	9	9	0,01	99030,07	3842,668282	4626,678767	4926,572167	13226,27189	7975,576225	9393,183459
P08727	13	10	0,00	44106,07	8187,3244	6594,921819	5466,719511	15291,37814	16743,80082	14540,16418
P31689	3	3	0,02	45609,63	3736,101103	1789,611275	2311,112284	8037,570997	5425,223231	4647,289315
Q13367	3	3	0,01	119686,8	2849,174271	1792,600256	1415,335374	5563,767967	5015,236737	3510,077284
P39023	20	17	0,00	46394,11	18338,94199	18371,44454	18267,15572	37046,45252	50586,80949	40800,08209
Q9Y3C6	3	3	0,00	18350,93	3268,316023	4569,904164	4413,897993	10790,94309	10098,63015	7741,077448
Q9Y6N5	7	5	0,01	50245,94	3218,728487	4673,018412	3234,427456	7764,307612	10942,27302	7346,914398
P0DP23; P0DP24; P0DP25	7	7	0,02	16837,65	22585,71875	26174,88075	31952,39085	91391,09743	39281,32349	58675,43573

O75131	4	3	0,00	60986,14	1557,295951	1430,768435	1676,306917	3769,935598	3694,750647	3484,848597
Q9H4M9	12	11	0,00	60684,01	8594,032254	6908,787531	9245,163295	22359,97541	18021,5752	17737,26253
P28072	3	3	0,01	25585,89	1880,072266	1618,289428	1056,843581	4437,179623	2975,902384	3324,433901
Q08211	29	26	0,00	142270,4	20426,47933	28519,06675	23215,37485	59917,66989	64360,44395	45926,44002
P41091; Q2VIR3	5	4	0,00	51679,84	2358,857473	1862,415147	2262,806264	6488,522521	4151,190133	4911,117829
P0CG48; P0CG47; P62979;P 62987	14	14	0,02	77038,71	62685,15977	46551,03265	100244,3548	229070,1857	134427,9156	140097,2255
P05783	15	13	0,00	48057,89	9556,333514	10415,62661	12075,67883	32643,2149	24046,4932	20886,51696
P20020	10	9	0,00	135654,5	4762,062506	4663,935843	4926,346761	15265,66926	10094,72482	9458,205264
O00159	15	13	0,00	122537,2	6165,358	5344,440211	8899,174748	18233,5081	16235,9977	15128,07024
P0DMV8 ;P0DMV 9	12	6	0,01	70337,46	5421,569282	6738,753078	6638,964267	20907,88369	11752,01502	13043,58835
P78386; O43790; P78385; Q14533	5	4	0,01	57341,92	2600,847314	2256,87876	1540,961817	5642,710607	6193,896554	3774,046286
P61204;P 84077	14	7	0,01	20657,84	10614,00251	8507,994804	9925,309557	32099,95939	19767,62018	19002,80593
Q8NBS9	9	9	0,00	48313,24	16996,46476	14240,27313	17265,7414	44904,20088	39943,95634	34714,30278
P13639	32	30	0,00	96307,82	40309,98375	25526,98273	31135,03584	92583,17706	74917,73949	73005,96586
P55786	10	7	0,00	103960,8	5558,441141	6414,552376	5622,73782	17405,18223	13704,86835	12936,08931
P84095	5	4	0,00	21764,74	886,711432	1003,802084	982,1322607	2782,715938	2458,78144	1954,137815
Q9BWM 7	8	7	0,00	35845,57	5983,304939	4258,283813	4816,479965	11245,67035	15855,35496	10688,28168
P60842	22	10	0,00	46382,16	16853,00751	14003,22217	15467,35503	42650,18897	34884,4396	39134,24068
Q9NS69	3	3	0,00	15521,66	1916,169634	2519,403364	3023,918154	5696,567158	7725,274584	5433,253862
Q12905	11	11	0,01	43290,31	7274,355612	6001,567438	6385,319201	23303,63444	13419,529	13363,4976
P31153	5	5	0,00	44002,9	2641,000163	3184,360178	2945,377565	6408,421268	6443,944219	9558,641721
P04899;P 63096	9	3	0,00	41021,25	3516,78459	4073,028569	3417,807836	10636,31319	8539,374379	9075,000184
P22314	8	7	0,00	118932,7	3530,438775	4890,096652	4711,709579	10457,13254	12620,53174	10650,38083
Q14152	10	10	0,00	166968,8	3668,14567	4170,728813	4332,455975	9909,331113	10781,45479	10685,0976
P53396	10	10	0,00	121751,9	4018,854075	5769,29729	5263,224198	14881,23133	12112,79242	12096,28736
P23229	11	8	0,00	127803,2	4912,913082	4004,938055	4325,9669	12179,11527	10614,54291	11637,44132
Q8TCT9	6	5	0,00	41773,55	12322,03158	9643,961949	8415,994998	29745,90736	28850,74417	20553,86999
P16152; O75828	7	7	0,01	30660,12	4828,046947	9720,499361	6757,707359	21798,41677	16458,67222	17436,42938
Q9Y490	23	22	0,01	271934,6	12995,85963	8069,106606	9279,994202	34750,6619	21665,79718	22929,36401
P04439;P 13747;P1 7693	7	3	0,01	41125,93	10603,84323	5154,963265	10995,87005	28578,8269	22223,52791	19323,22588

P35606	9	7	0,00	103342,8	2697,080993	2009,655604	3198,761342	8160,058215	6217,200951	6518,717628
P04075	18	14	0,01	39876,28	17311,55101	22812,97012	26680,31265	88411,02646	38885,51938	50044,31756
P61163	8	5	0,00	42727,87	2282,68103	2647,217054	2892,474084	9123,381258	6134,63806	5545,067118
Q00610	56	44	0,00	193382,8	39605,70095	46117,9185	48229,08774	130087,4529	120920,0859	105430,4423
O43707	47	28	0,00	105310,4	35969,60804	35435,57593	33956,53326	126999,4306	69813,39431	84587,3786
P08254;P09238	10	10	0,00	54262,54	10288,94746	8422,826145	8714,109275	31635,61563	19724,85685	21894,32301
P06213	5	4	0,01	159013,4	909,139993	1330,098604	1953,639528	3836,23149	4208,538947	3170,061568
Q15393	12	9	0,00	136661,1	22119,9266	24372,88014	23338,74459	79264,46284	55133,32028	52484,52078
P68032;P68133;P62736;P63267	22	3	0,00	42361,25	97031,98816	90687,70278	94027,70835	298394,1388	235043,3957	220693,6671
P63241; Q9GZV4;Q6IS14	8	7	0,00	17060,4	4377,160859	3690,531556	5037,709007	13946,51344	9565,60563	11793,73312
P29692	10	7	0,01	31235,93	9171,645208	9782,174075	9496,947303	38059,21259	17058,32333	21654,82551
Q13200	11	10	0,00	100941,3	4093,152157	4625,897558	4528,58977	13635,65156	10438,40444	11682,91697
Q15738	4	4	0,00	42185,5	1492,85668	1205,775121	983,8329602	2955,361495	3679,089772	3323,099907
P24534	3	3	0,00	24934,84	7590,530304	6631,65103	7626,292518	24729,18572	15116,25142	19333,50032
Q15019	8	7	0,00	41715,67	7247,848419	7076,040017	5523,901663	23102,16163	15262,23845	15774,15376
Q02790	5	3	0,01	52089,79	207,9025617	190,8926277	205,0009361	858,6899691	361,6631804	428,7381193
Q15365	7	5	0,00	38011,15	3987,251749	3937,043657	5386,173661	16265,96503	10185,06212	10013,52559
P62937; A0A075B759;A0A075B767;A0A0B4J2A2;F5H284;P0DN26;P0DN37	12	9	0,01	18240,64	50740,35967	54632,85216	65304,51292	238068,5393	96034,82436	134501,5293
P19367	13	11	0,00	103626,7	4695,156773	5549,405525	5039,627756	16200,7167	13389,5117	12417,45237
Q02878	10	10	0,00	32784,99	14172,49052	10399,52268	10711,222	27777,35763	41506,09274	28028,82464
P32969	5	5	0,00	21977,5	6228,232431	6961,409475	7337,175309	19014,02604	21916,10032	16111,34318
P08729	10	5	0,00	51442,77	4498,540049	4527,907327	3523,7128	14076,41236	10647,55582	10204,36528
Q06830	11	9	0,00	22338,49	28822,63245	26915,16666	24546,2636	97828,398	59553,14584	67269,83781
O15144	7	7	0,00	34447,13	2282,0465	2841,389243	2910,494693	10145,06835	5706,130892	6665,746267
P37802	9	7	0,00	22562,59	12526,64628	12431,11272	13042,29973	44413,50024	30293,53264	31863,22291
Q13247; Q08170	4	3	0,00	39700,89	4401,56737	5704,437563	4665,827201	14817,41999	15697,25627	11067,93968
Q8WVM7	4	3	0,00	145397,1	6892,899116	5863,615694	7061,302616	19651,93022	16964,28985	19267,24508
Q9ULV4	8	8	0,01	53933,47	3908,985856	3307,126051	3701,426262	14348,54298	7130,860028	9327,985098
P35998	9	8	0,00	49033,13	2222,830827	2634,844511	4013,717913	9739,512677	7931,91074	7424,500773

Q07065	24	18	0,01	66136,59	16738,94972	28857,62476	33283,37522	77177,33679	79309,08555	67016,53539
P62714;P 67775	3	3	0,00	36145,43	1713,124737	2069,601435	1863,602703	5981,716131	4467,898966	5603,833961
O00148	10	3	0,01	49642,87	1091,116636	750,6119119	1445,604234	2797,525428	3764,640348	2810,154427
P60660	10	7	0,00	17101,18	11030,52072	15091,89354	14207,85859	42199,10531	37928,40069	35199,8065
P05026	5	5	0,00	35460,54	5116,436637	3335,272728	3382,491543	14667,32339	10127,37913	9100,612997
Q15758	8	8	0,00	57054,65	13305,60662	12551,16924	11964,01069	43893,62326	32380,04265	32676,30386
P54920	5	5	0,01	33689,02	1723,657441	2182,194533	1858,643112	7841,874555	4131,828043	4736,766768
Q07020	5	5	0,00	21748,58	8579,517578	10181,73362	8943,80629	22547,63641	33004,76599	24822,29852
P22234	7	5	0,01	47820,64	1662,908791	2266,48736	2077,881421	8154,284913	4064,201836	5224,037582
P13073	4	4	0,01	19633,79	4222,088696	4094,910344	1513,252134	8722,786057	11851,39487	8188,600019
O75915	3	3	0,00	21614,79	2639,203756	1928,971333	1388,509528	5983,113791	6506,211228	4964,170639
P42704	21	18	0,00	159102,9	8074,023875	8536,103445	8447,531231	27924,86061	24737,09525	21054,36014
P12814	38	17	0,01	103628	18481,94995	19067,99982	18823,89984	82028,05151	35041,11737	49311,64701
P60228	11	10	0,00	52620,11	4832,752106	3220,257555	4005,413073	13010,85084	11867,49842	10788,43478
P53621	12	10	0,00	139885,7	3812,143639	3620,843055	3681,076265	12518,28889	10696,69902	9752,765622
O76021	5	4	0,01	55200,67	1215,503152	1232,842107	1120,877863	2824,661366	5090,993476	2690,203379
P60900	5	5	0,02	27855,72	3506,046769	3124,307721	3575,564513	16646,38308	5818,202883	7929,330043
P07900	38	13	0,00	85059,06	20889,55247	21737,42013	17908,86416	83045,2096	45054,82217	52930,41869
P59998	4	3	0,00	19781,12	1325,119933	1308,812706	1334,810377	4938,194813	3310,443469	3718,938756
P54577	8	5	0,00	59485,73	679,0713944	1101,646086	752,2845168	2877,695029	2215,908182	2549,000476
Q01813	17	14	0,00	86508,68	15226,57001	11137,68021	9616,431443	41188,5139	34263,71104	33129,1587
P35613	7	7	0,00	42599,61	6057,242444	8550,267801	5423,105056	25758,02074	18142,45079	16623,54115
P06744	10	9	0,01	63375,35	4095,242832	3184,336803	4312,692974	16014,79777	7946,130287	11105,48129
Q96QK1	8	8	0,00	92505,57	2627,35898	2508,097104	2535,544702	8984,155633	7480,653588	6851,961341
P17858	4	3	0,00	85817	2315,80533	1799,875401	1497,840073	5605,604566	5207,693905	6255,780131
Q9UHB9	5	4	0,00	71242,96	1230,7751	1130,091554	1818,897368	5301,837674	4260,375268	3175,565169
P57088	3	3	0,00	28320,38	917,2791293	1013,907837	588,7195916	2657,792865	2548,838979	2473,580676
P13473	4	4	0,00	45531,05	5342,374208	3886,72667	3227,022407	15449,65835	13193,57761	9385,879142
Q16822	3	3	0,00	71497,49	3784,558355	4661,872164	4366,536419	12105,88398	15542,44954	11541,1362
Q9UNM 6	13	13	0,00	43230,71	6702,862182	4623,698793	4288,459143	18289,57514	14978,8433	14711,12065
Q96CW1	4	3	0,00	49996,94	2960,829374	2925,346425	2420,786629	10349,88452	7703,059333	7518,252398
P13797	12	7	0,01	71324,4	2847,307836	3353,089849	4225,242345	15008,2486	8225,62088	9036,811859
P27701	3	3	0,00	30252,88	1308,734979	1070,78518	1322,552221	3949,23174	4362,99567	3146,942238
P63104	18	14	0,00	27916,24	28236,87877	23188,48315	23809,43193	108184,6117	57738,77583	66967,98554
P05362	7	7	0,01	58623,66	3825,975237	5695,303035	3773,230996	21138,60182	9752,005614	10352,68092
P50281;P 51511;Q 9Y5R2	13	11	0,00	66236,21	9282,273742	9666,148249	8797,8382	36744,60199	23886,13315	25497,19387
P46776	3	3	0,01	16675,46	6130,418581	4525,772774	1962,910017	10748,90543	16498,72058	11951,53722
P60903	4	4	0,01	11317,21	9629,377413	8350,540262	11913,39472	45686,95447	21935,85256	25803,58645
Q9NZ01	5	5	0,00	36433,64	2654,018155	3783,589416	4266,196948	10093,0665	13225,85523	10157,51407
P20339	6	4	0,00	23886,83	2396,529874	2785,959403	2090,820938	8413,746446	7485,366107	7038,318363

P24752	8	7	0,00	45484,81	2723,535528	3072,735961	2944,581227	10352,70046	7846,616197	9467,409794
P18206	7	6	0,01	124369,9	1206,505352	1693,001369	2304,060083	7354,007472	4256,501625	4865,045845
P31946	9	3	0,00	28196,51	2583,379948	2637,906639	3171,104292	10223,11271	8491,847916	7868,379226
P20073	3	3	0,00	53024,5	431,731814	641,1791459	490,5170245	1543,960551	1909,846149	1504,390693
P21333	43	35	0,00	283476,6	27423,43381	30964,39477	32084,99342	130071,2842	73776,5702	89376,27162
Q8N5K1	3	3	0,00	15506,32	1309,048616	2053,398693	1482,433336	5738,12534	4898,369941	5412,418422
O43852	10	10	0,00	37220,91	9775,48823	11218,47165	13263,26204	46009,49216	34996,31768	33295,22312
Q06210	10	10	0,00	79604,95	3427,6808	5636,62559	2919,910926	15763,17905	12106,34088	12126,88537
Q9BSJ8	11	10	0,00	123369,8	6432,354609	5038,023093	4759,778464	18160,45614	20165,82668	16134,13761
P09211	5	5	0,00	23583,97	3649,889668	5831,169921	4912,994077	18288,87058	14785,14374	15312,49756
Q13620	6	5	0,00	104552,1	2025,925801	1830,643903	1892,125884	5970,62567	7849,629575	5530,744569
Q9H3N1	5	5	0,00	32190,46	4091,412501	4415,148694	4283,930679	16144,08044	14477,85293	12710,60803
Q15286	5	3	0,00	23310,39	1452,98522	1252,207185	1666,979646	5681,043172	5035,031728	4097,476267
P05023	30	18	0,00	114207,9	25579,92991	27867,11974	25570,50774	106215,9204	89080,31168	74184,10447
P13796	6	3	0,02	70858,8	1070,393849	293,8213765	1107,66858	2110,23194	3449,340587	2902,347895
P00387	7	7	0,00	34463,05	7223,477372	8192,008639	8227,594949	28867,69199	29267,08774	23145,30786
P60174	16	16	0,01	26954,66	15872,64486	18115,23981	19675,88188	91807,54234	38034,87303	55260,36893
Q9BTM1;P0C0S8;P20671;Q16777;Q6FI13;Q96KK5;Q99878;P04908;Q7L7L0;Q93077	9	6	0,01	14019,42	48489,71647	35587,05937	62647,7265	256137,351	98412,78873	153996,8978
P13645	5	5	0,00	59055,27	1229,124216	1457,811341	2236,229838	7279,794145	4841,519432	5067,099771
Q9UHG3	6	5	0,00	57039,45	2558,0593	1804,224141	1650,959071	6678,365904	7451,726463	6870,03487
Q8NC56	3	3	0,00	57374,44	493,7337888	448,305108	488,3118166	1839,597181	1782,683442	1391,882358
P18669;P15259;Q8N0Y7	13	12	0,00	28918,04	14942,22297	13390,77958	19026,49458	78649,9175	39673,65411	47904,45679
Q99536	8	7	0,00	42148,46	14421,28159	17101,73809	12514,72843	61919,61615	47489,85859	45706,84017
P04632;Q96L46	8	8	0,00	28486,87	5701,907274	7598,82921	6249,100409	28537,05355	18353,72513	22177,68634
P40939	16	12	0,00	83741,15	12980,76879	9600,6076	7694,005248	41697,53695	33920,50265	31622,88649
P35579	96	73	0,00	227787,2	90588,46316	102310,0299	89399,46897	352045,8255	368269,28	280976,381
P36871	6	6	0,00	61734,36	1771,231954	1387,420978	2707,081949	8057,914362	7068,511853	5761,265494
Q15363	6	5	0,00	22875,35	8198,774158	7255,83221	8586,266568	31240,15624	37072,70437	17362,95939
O60493	3	3	0,00	18819,4	319,5733056	331,2412576	273,2966322	816,2815287	1415,444445	1066,47588
P05787;Q86Y46	22	15	0,00	53704,36	17822,42557	15649,27621	13220,75966	70251,14289	49581,10586	47809,02246
Q13641	4	3	0,01	46602,2	1562,245303	1205,169095	817,6313534	6509,402942	2576,470299	3817,191909

P26006	12	12	0,00	117810	4399,532934	4318,978368	4141,825135	17153,93915	17607,82079	11571,55999
P04233	3	3	0,00	33971,91	2602,366368	3634,358307	3095,750049	11227,67159	12774,66331	9652,515621
Q99460	10	10	0,00	106863	3622,724221	3399,676028	4101,673682	16478,6766	11787,79502	12070,36686
P07339	13	12	0,00	45065,55	16298,26217	12260,78005	11978,76196	65600,34293	36506,54141	45791,08691
P09382	7	7	0,01	15057,89	9914,058465	9169,630034	10440,78117	57210,8156	21808,82521	29318,8697
Q9H0C2	6	4	0,01	35307,07	848,9024983	2398,874991	1113,477931	5887,735388	4717,873084	5428,494162
Q03135; P56539	6	6	0,00	20642,73	8433,29539	8162,638341	10410,05115	31248,17977	43600,63682	24539,10798
P08133	24	24	0,00	76215,59	19615,86098	21166,76373	21478,95367	85543,04192	80275,9623	66144,70943
P51572	3	3	0,00	28048,68	2944,770412	3107,80208	3437,393577	11017,67781	13755,03167	10623,48293
O60701	3	3	0,01	55708,49	638,9777401	905,1878856	1566,615623	5044,813915	2889,883034	3675,932216
P48960	11	10	0,00	94663,74	2974,779983	3166,139534	3071,407529	12818,3258	11498,66733	10379,43957
O75367; Q9P0M6	8	8	0,00	39788,19	15823,77648	17291,06042	18381,41877	78615,67588	53501,94721	63446,2834
P43686	3	3	0,00	47480,4	963,8386999	1297,287482	1889,830989	7188,676235	4240,482665	4350,02816
P12956	16	13	0,00	70128,33	9772,344413	10420,4205	11811,54683	48678,2214	34674,93112	38338,19197
P35580	25	9	0,00	229968,9	11336,34561	8415,240082	7912,919726	34604,60715	39990,82862	31920,29711
Q14103	9	6	0,00	38605,31	1942,061559	2817,941629	2482,565591	11397,94226	7746,41797	9008,927128
P61158	7	6	0,00	47827,47	4336,589477	5513,022455	5189,474614	24940,88204	17001,4934	16525,06393
Q9H5V8	7	6	0,00	94357,55	2026,060688	1144,147196	1594,481524	8439,654883	5272,801305	4834,062465
P53618	19	19	0,00	108283	3537,409791	4234,513146	6643,859223	24955,21761	15527,98004	15774,2502
O14950; P19105;P 24844	7	7	0,00	19836,25	11533,11084	9586,920075	9569,734305	52180,41373	33759,70109	33830,4183
P30086	3	3	0,00	21170,86	816,1648943	819,2541516	1172,049441	4945,436572	2503,374147	3587,631673
Q16555	8	7	0,00	62749,94	1590,613384	1472,249845	1510,353651	8050,941789	4201,518445	5809,489612
P21980	12	11	0,00	78469,48	10094,24589	7939,0351	5163,99579	31616,05796	33088,81247	26949,63327
P54709	6	6	0,00	31854,81	2957,838752	3557,132561	3779,357302	15138,3507	13600,64917	12398,82958
P02792	5	5	0,00	20076,74	3986,075107	6517,197195	4102,399366	25499,59585	16749,20056	16137,65522
P07437; A6NNZ2 ;Q9H4B7	32	5	0,00	50127,16	21216,68922	20576,64693	23211,38079	89205,2089	85946,65889	84829,86258
P30040	9	7	0,00	29050,51	10901,21168	7115,634916	7476,84431	42878,41675	30593,06091	29127,4884
P11310	5	3	0,00	47044,65	1149,801106	987,5087268	843,298311	5143,56392	4207,096586	2651,899342
P49257	9	9	0,00	57834,17	8390,391393	7555,99373	6842,26484	33433,00618	35337,77796	23304,81194
P07355; A6NMY 6	31	29	0,00	38832,22	100347,7675	82313,94388	85896,72609	437364,7856	336055,2272	313972,3152
P36542	3	3	0,00	33053,15	2800,247978	6157,597126	3522,64774	19452,07084	15331,76159	15890,11709
Q9HC07	3	3	0,01	35076,89	520,9652179	461,3138708	986,5868434	1708,606327	4251,27344	2041,783151
P32119	5	4	0,01	22063,02	3594,940134	2139,748704	1351,61364	13973,09905	6571,314169	8319,467056
P49327	15	15	0,00	276050,2	6200,431755	7790,06165	8718,937911	35205,38326	30215,65083	27449,48486
P00492	4	4	0,00	24807,54	7572,286651	9016,617237	6692,140575	34801,07539	33662,60266	26749,31321
P18754	4	4	0,01	45425,33	533,0149955	674,6936363	933,3506317	4876,848855	1894,045074	2040,45696

P42166	11	3	0,00	76062,4	372,8397237	473,0807914	387,4940105	1522,544	1936,709406	1669,274304
P55795	9	3	0,00	49548,84	541,2774964	453,5004527	399,2302999	2342,692339	1787,523321	1689,651236
Q06323	6	6	0,00	28894,23	2180,47294	2412,983448	1499,194138	11234,69087	6961,06182	7522,670865
O75083	10	9	0,00	66877,93	5699,292444	5944,22157	6469,760849	28096,4949	23282,40696	25475,79262
Q08380	8	7	0,00	66243,52	3893,94239	3686,546362	4941,076649	24444,70418	13805,1648	14929,21715
P27824	23	22	0,00	68024,62	30138,57317	37654,6215	41392,75162	166447,4289	179027,1936	119224,4811
P02794	7	7	0,00	21396,76	9258,535587	6545,005185	5646,289062	41492,09952	27478,33677	23474,54998
Q16352	7	3	0,01	55561,77	907,9677304	353,0101365	373,4147407	2974,444951	2304,844061	1781,59437
P15531	9	3	0,00	17319,84	7350,833362	8051,002792	7180,704595	37131,10869	31278,60368	29894,92676
P35237	3	3	0,00	42964,19	3767,495114	3003,212757	2461,391669	18742,98311	10794,38991	10685,18701
P22392; O60361	12	6	0,00	17412,13	2284,613932	2006,642453	2025,783596	13350,02369	6277,328853	7979,516917
P00558	23	17	0,00	45013,98	33153,08588	43414,00205	43573,00324	258554,0107	125571,7167	141413,0481
P21399	8	7	0,00	98912,29	2208,70041	1997,767689	2113,223475	10224,65185	9522,986995	7934,684404
P18085	7	3	0,00	20624,88	3682,686796	3679,684695	5585,323652	22678,38928	16416,37491	17810,37201
P48643	14	14	0,00	60127,41	5281,733547	6441,276325	7614,544815	37285,41681	23884,06526	23922,08228
P51571	5	5	0,00	19169,72	9693,475201	10036,69605	8018,853691	45527,24862	43567,49752	33180,3548
O75531	4	4	0,00	10286,7	4370,700056	4846,512746	5063,171222	19888,90336	23566,35268	20785,22832
Q02978	5	5	0,00	34232,87	1094,799161	950,5604676	1813,362461	5075,540619	7269,633073	5054,581074
P30740	5	5	0,01	42855,95	2347,776715	2470,083237	1268,201082	13668,38012	6033,550358	7858,31426
O15121	3	3	0,01	38037,21	960,7327635	231,3688671	219,167411	2186,205036	2205,437789	2009,702126
P78417	6	5	0,00	27851,05	4473,945769	2223,188596	2509,928433	18280,8079	11532,59586	12283,3968
Q9BTV4	11	10	0,00	44932,66	4325,417899	2306,039183	2991,075541	17057,18465	14048,68357	12912,00998
P35968	4	4	0,00	153408,9	3390,776591	3454,135849	2791,411278	17081,37992	13417,67056	13764,63362
P05556	18	16	0,00	91722,99	15035,16328	19649,18995	14566,8698	106092,5857	62689,67781	62115,3181
Q70UQ0	5	4	0,00	39423,1	2837,085922	1350,944348	2140,303932	10144,45949	11215,36995	8383,020776
P08195	19	18	0,00	68222,21	12507,00398	12874,61136	13146,26045	80590,09023	50943,97758	49951,05523
Q8NBQ5	5	4	0,00	33278,04	1569,288284	1304,301633	1110,279722	5900,504511	7073,612613	5873,359571
Q16836	7	7	0,01	34350,64	1974,726086	1624,863897	2287,151564	13889,9022	3992,044584	10078,87344
P21589	17	15	0,00	63938,12	9805,234996	12423,97643	8289,856147	62056,25795	43984,67038	40075,02535
P52565	4	4	0,00	23264,17	3495,842617	2797,995687	4570,89766	25385,43856	12545,51838	15193,26614
P06703	5	5	0,00	10236,78	19437,67793	9353,218363	12498,0555	83551,20522	58323,72884	60911,90185
P56192	4	4	0,01	102313,5	200,2101148	613,8501887	289,6880348	1426,037789	2185,898198	1831,226516
Q6DD88	7	6	0,00	60998,31	4397,072681	3305,77691	3424,831709	18768,9196	19181,68888	17089,08475
Q9HDC9	8	7	0,00	46651,55	2727,679298	2272,867778	2121,401491	10498,08464	14677,367	10295,58751
P12268	15	13	0,00	56261,31	4452,565112	4791,56586	4511,184561	25506,92684	23627,54398	20516,43513
O00487	5	5	0,00	34748,24	3322,987076	2990,705093	3266,011619	19199,03172	14853,3544	14909,78112
P17301	16	14	0,00	130550,2	7268,141314	9707,355818	8640,22904	58982,58511	35599,47885	36697,03905
P52789	10	9	0,00	103805,8	4763,846718	4810,66841	4660,250718	28603,11975	25166,93455	19553,07038
O95816	4	4	0,00	23943,06	8595,797494	5689,223051	6413,699327	43564,04192	30739,33472	33145,91495
P14314	12	10	0,00	60394,97	7402,553883	6815,215721	8261,014899	41895,39047	43244,2162	31570,56363
P51648	3	3	0,00	55304,14	344,4925438	345,9140523	460,6545998	1953,999844	2432,766594	1595,503647
P04083	22	21	0,00	38942,43	31817,68683	23586,751	26565,70412	166171,1852	140494,4903	119660,526

P17655	13	13	0,00	80850,87	4998,192196	4379,260993	4562,184874	34146,26964	19825,90144	18924,39573
P35354	13	13	0,00	69737,6	5712,961408	6129,790824	6202,581503	51046,56562	20993,19471	22479,18961
Q92841	16	10	0,00	80956,91	8471,127947	7655,121512	7394,827164	50098,75525	31857,22863	42058,22558
Q8TC12	4	3	0,00	35785,48	287,1077599	201,8319242	394,2198714	1789,358669	1363,731011	1504,744135
P10599	5	5	0,00	12022,66	6481,325397	8765,447795	6607,153346	55730,8429	31211,49546	28678,80978
P41250	11	10	0,00	83907,08	1351,857757	2271,552842	2235,641563	11635,18571	8405,274519	11232,2124
Q9P212	3	3	0,00	261908,5	922,4537286	953,9336529	794,1063262	7469,979229	4190,801012	3107,473369
O15427	3	3	0,00	50096,78	1164,987076	891,0380634	941,0113095	5752,191449	5822,578683	5176,361174
P35268	3	3	0,00	14844,04	3703,233227	3316,41243	3688,296435	25271,1599	19041,7304	15919,10682
P55209	10	7	0,00	45659,35	8565,225211	5607,437682	7294,725244	56502,91281	32610,60986	33172,21586
P40121	9	8	0,01	38783,72	2875,406398	5363,828884	5759,921536	39866,57243	15924,45593	24322,16802
Q96N66	3	3	0,00	53449,38	1172,965227	1041,217318	772,8866477	5154,720649	7055,624708	5101,149346
P08758	12	12	0,00	35993,86	14236,29265	13486,19227	13853,1055	99020,04089	72426,33995	71252,66026
P43490	22	20	0,00	55806,34	23652,07674	17316,71096	15448,18382	170013,1371	69815,4629	89831,07375
Q99829	5	5	0,00	59686,17	1075,623771	1050,203872	934,9262958	4399,590209	8182,127166	5450,535842
O60664	7	7	0,01	47246,13	1569,067434	1082,173945	755,8155553	11346,25648	3669,849776	5245,231943
P06733	31	25	0,00	47511,2	45250,20774	44841,88766	44840,91664	400769,0573	170375,3111	231857,8215
P16070	8	8	0,00	82050,98	11075,74338	9950,303873	11364,24262	81637,07211	57888,80333	53542,19286
Q9H9B4	5	4	0,00	35904,65	564,0335135	638,2549356	1206,309334	6013,236453	5311,487326	3041,430097
Q15691	5	3	0,00	30170,21	659,2182853	923,7893308	534,4252087	5830,456084	3356,314389	3523,837688
P19013	4	3	0,00	56543,31	1023,624267	1186,940646	1976,799519	12724,9551	6960,819445	5652,249498
P12236	14	3	0,00	33094,44	1260,222812	1428,093994	1238,217339	7553,068271	9660,558774	7017,094989
P68104; Q5VTE0	27	16	0,00	50483,12	53840,21337	41679,24447	60978,24542	528834,3268	198999,2877	247469,5588
P37837	5	4	0,00	37711,28	1068,873758	1694,82968	1507,107631	10535,01899	8299,571045	7857,179356
P02786	30	28	0,00	85327,71	28240,31882	29088,54979	26517,13414	213335,5425	158341,2652	158732,4823
O43399	7	5	0,00	22294,78	1098,66995	1107,079737	1109,936164	9946,395976	4673,316333	6550,078589
P00338; Q6ZMR3	20	17	0,00	36973,93	38710,87619	30272,47396	26312,74108	298275,5371	139780,3756	182346,4588
Q13501	11	10	0,00	48485,64	8475,663449	9241,69571	8756,65312	94720,89143	32106,75306	51799,67245
A6NCE7;Q9GZQ8;Q9H492	3	3	0,00	14685,05	1958,509284	1245,314515	1729,09303	16273,80904	7239,704712	10015,04382
P61769	3	3	0,00	13828,64	368,6842283	582,1105258	1035,067488	5152,305627	5264,653357	3636,87283
O14880	3	3	0,00	16744,44	265,9896501	373,1482196	467,4708308	2408,874858	3324,213314	2140,239827
Q92597	10	10	0,00	43291,8	4160,479706	4594,64946	5956,518743	59200,85574	19368,20736	26420,34411
P05455	4	4	0,01	47008,2	239,9929948	561,5066899	657,065699	3881,195091	4632,340258	1992,656731
P29401	14	13	0,00	68562,07	2490,460683	3133,078857	4043,505261	33422,67918	16372,52394	20711,53353
Q9BQE3;Q9H853	25	3	0,00	50579,74	337,9609851	257,0511727	287,308618	3198,045242	1873,354318	1365,473206
P14618	31	28	0,00	58507,28	57455,20899	50240,65161	59784,91815	638904,7371	334145,2789	345819,6565
P01889	7	3	0,00	40802,29	1816,634471	1112,330662	1691,177	8945,157891	16028,19808	11605,89128
Q01518	10	10	0,00	52357,76	2129,971456	2659,868439	1938,334097	27308,59014	11788,58475	14427,80902

P07195	12	9	0,00	36923,7	2696,71343	3375,826844	1564,939166	29384,70856	14811,70595	16773,82299
Q07021	4	4	0,00	31761,46	2527,268955	2774,831664	2508,672201	29612,32359	16890,98866	16011,4614
P07737	11	10	0,00	15225,35	15342,28533	16313,00836	12781,91906	176398,1658	81109,69589	115855,1543
P23528	11	7	0,00	18730,63	6459,21454	7423,004048	7426,453889	102741,8188	36293,26351	48469,88679
P42858	7	5	0,00	351595,8	276,1406177	226,4858282	204,301936	2315,844345	2482,093023	1498,230854
P00403	3	3	0,00	25736,17	682,3397753	650,2361328	1320,421416	7123,34857	10534,66648	7425,337526
P17096	3	3	0,00	11676,03	1051,239877	465,5829667	992,7628914	7936,705165	8513,878237	7787,462698
P03956	30	26	0,00	54178,07	27796,65539	23656,6355	23156,25723	394588,9422	135688,7767	204028,4745
O00299	7	6	0,00	27264,92	1887,195405	2711,448522	2520,014521	23855,56959	22370,93406	23875,96623
Q6PIU2	7	7	0,00	46093,05	1103,032703	1236,071714	780,0402075	10280,32888	12698,43205	9074,658537
Q68DX3	4	4	0,00	145935,7	110,1621138	86,7295467	102,6848859	1251,818265	1136,918608	845,7002548
P04406; O14556	27	23	0,00	36224,37	32694,32801	32726,70642	31273,61822	493886,1087	298586,047	369969,6327
P00533	8	5	0,00	137699,3	833,9285823	640,3252808	589,334905	12400,04944	7757,993908	6749,417837
P80723	3	3	0,00	22693,42	69,39720211	153,6723104	457,4560506	3586,778419	2489,105881	3290,752623
Q9NQC3	12	11	0,00	130330,8	2106,98233	1982,658179	2193,765573	40316,00463	25343,22344	24171,68029
P01903	7	5	0,00	28778,05	690,273066	1280,168515	1118,041404	20825,7156	13492,67734	10095,18914
P49588	3	3	0,01	107552	870,0876819	345,4680474	955,4299191	20125,9256	5711,110161	6074,225186
O43169	4	3	0,00	16808,63	485,0364006	418,1294562	310,680366	8433,432198	6436,367881	4486,474813
P30613	5	3	0,00	62229,46	2117,311432	2634,639463	2686,235163	64366,7644	22813,18433	33187,30611
P46100	3	3	0,00	285038,4	675,172853	486,151188	518,6539826	10952,27781	11443,01363	9023,884436
Q96S97	3	3	0,00	36072,02	71,91008689	134,7562181	196,6644036	2618,00707	3071,695547	3680,848477

Table S4.

Gene	Forward (5´→3´)	Reverse (5´→3´)
STC2	ACTACTCAACTCTGCCGTCC	ACGCTTGGTTTCTTGGTGTC
MT-CO3	CCTAATGACCTCCGGCCTAG	GGCTAGGCTGGAGTGGTAAA
ANPEP	ACCTGAGCTACACCCTGAAC	GAGAATCGTCGTGTCACTGC
CASP3	ATGGAAGCGAATCAATGGACT	TGCATACTGTTTCAGCATGGC
CASP9	CTCAGACCAGAGATTCGCAA	GCCATGGTCTTTCTGAAGAC
BAX	TTGCTTCAGGGTTTCATCC	CAGCCTTGAGCACCAGTTT
BCL-2	GGTGGGGTCATGTGTGTGG	CGGTTCAGGTACTCAGTCA

Table S5.

**Groups**	**Tumor Sizes (cm)**			**Tumor Volumes (cm3) (WxWxL)x0,5**	***Std. Err.***	***P* *(vs Control)***
	**Width**	**Length**	**Equation Constant (0,5)**			
**C1**	1,3	0,9	0,5	0,7605		***0,002****
**C 2**	1,5	1,25	0,5	1,40625		
**C 3**	1,2	1,2	0,5	0,864		
**C4**	1	1,4	0,5	0,7		
**C5**	1,8	0,9	0,5	1,458		
**C6**	1,6	1,2	0,5	1,536		
**Mean**				**1,1208**	0,1571	
**U1**	0	0	0,5	0		
**U2**	0,6	0,4	0,5	0,072		
**U3**	0	0	0,5	0		
**U4**	0	0	0,5	0		
**U5**	0,2	0,2	0,5	0,004		
**U6**	0	0	0,5	0		
**Mean**				**0,0127**	0,0119	

Table S6.

Weeks								
	1		2		3		4	
Group 1	W (cm)	L (cm)	W (cm)	L (cm)	W (cm)	L (cm)	W (cm)	L (cm)
C1	0	0	0,4	0,3	0,8	0,6	1,3	0,9
C 2	0	0	0,3	0,4	0,9	0,7	1,5	1,25
C 3	0	0	0,3	0,4	0,8	0,7	1,2	1,2
C4	0	0	0,3	0,3	0,6	0,8	1	1,4
C5	0	0	0,5	0,3	0,9	0,6	1,8	0,9
C6	0	0	0,4	0,5	1	0,9	1,6	1,2
Group 2								
U1	0	0	0,2	0,2	0,4	0,2	0,6	0,4
U2	0	0	0	0	0	0	0	0
U3	0	0	0	0	0	0	0	0
U4	0	0	0	0	0,2	0,2	0,2	0,2
U5	0	0	0	0	0	0	0	0
U6	0	0	0	0	0	0	0	0

Table S7.

	Weeks							
	1		2		3		4	
Group 1	Tumor Volumes (cm3) (WxWxL)x0,5							
C1	0		0,024		0,192		0,7605	
C 2	0		0,018		0,2835		1,40625	
C 3	0		0,018		0,224		0,864	
C4	0		0,0135		0,144		0,7	
C5	0		0,0375		0,243		1,458	
C6	0		0,04		0,45		1,536	
	**Mean**	0	**Mean**	0,025167	**Mean**	0,256083	**Mean**	1,120792
Group 2								
U1	0		0,004		0,016		0,072	
U2	0		0		0		0	
U3	0		0		0		0	
U4	0		0		0,004		0,004	
U5	0		0		0		0	
U6	0		0		0		0	
	**Mean**	0	**Mean**	0,000667	**Mean**	0,003333	**Mean**	0,012667
